# Optimizing the Harms and Benefits of Cervical Screening in a Partially Vaccinated Population in Ontario, Canada: A Modeling Study

**DOI:** 10.1177/0272989X251332597

**Published:** 2025-04-22

**Authors:** Daniël D. de Bondt, Erik E. L. Jansen, Christine Stogios, Bronwen R. McCurdy, Rachel Kupets, Joan Murphy, Dustin Costescu, Linda Rabeneck, Rebecca Truscott, Jan A. C. Hontelez, Inge M. C. M. de Kok

**Affiliations:** Department of Public Health, Erasmus Medical Center Rotterdam, Rotterdam, The Netherlands; Department of Public Health, Erasmus Medical Center Rotterdam, Rotterdam, The Netherlands; Ontario Health (Cancer Care Ontario), Toronto, ON, Canada; Ontario Health (Cancer Care Ontario), Toronto, ON, Canada; Ontario Health (Cancer Care Ontario), Toronto, ON, Canada; Ontario Health (Cancer Care Ontario), Toronto, ON, Canada; Ontario Health (Cancer Care Ontario), Toronto, ON, Canada; Ontario Health (Cancer Care Ontario), Toronto, ON, Canada; Department of Medicine, University of Toronto, Toronto, ON, Canada; Ontario Health (Cancer Care Ontario), Toronto, ON, Canada; Department of Public Health, Erasmus Medical Center Rotterdam, Rotterdam, The Netherlands; Heidelberg Institute of Global Health (HIGH), Heidelberg University Medical Center, Heidelberg, Germany; Department of Public Health, Erasmus Medical Center Rotterdam, Rotterdam, The Netherlands

**Keywords:** cervical cancer, cervical screening, human papillomavirus (HPV) vaccination, microsimulation

## Abstract

**Objectives:**

In Ontario, Canada, the first cohorts who were offered school-based human papillomavirus (HPV) vaccination are now eligible for cervical screening. We determined which screening strategies for these populations would result in optimal harms–benefits ratios of screening.

**Methods:**

We used the hybrid microsimulation model STDSIM- MISCAN-Cervix to determine the harms and cancers prevented of 309 different primary HPV screening strategies, varying by screening ages and triage methods. In addition, we performed an unstratified (i.e., uniform screening protocols) and stratified (i.e., screening protocols by vaccination status) analysis. Harms induced were quantified as a weighted combination of the number of primary HPV-based screens and colposcopy referrals at 1:10. A harms–benefit acceptability threshold of number of harms induced for each cancer prevented was set at the estimated ratio under current screening recommendations in unvaccinated cohorts in Ontario.

**Results:**

For the unstratified scenario, 5 lifetime screens with HPV16/18 genotyping was optimal. For the stratified scenario, the optimal scenario was 3 lifetime screens with HPV16/18/31/33/45/52/58 genotyping for vaccinated individuals versus 6 lifetime screens with HPV16/18 genotyping for unvaccinated individuals.

**Conclusions:**

We determined the optimal cervical screening strategy in Ontario over the next decades. To maintain an optimal harms–benefits balance of screening, the Ontario Cervical Screening Program could adjust screening recommendations in the future to reduce the number of lifetime screens and extend screening intervals to account for vaccinated cohorts. Stratified screening by vaccination status could further improve this balance on an individual level.

**Highlights:**

- People in cohorts who were offered HPV vaccination as part of Ontario’s school-based program may achieve a better harms–benefits balance if cervical screening recommendations are updated to a less intensive protocol in future. This holds for the cohorts as a whole (i.e., unstratified screening) as well as for both vaccinated and unvaccinated individuals in these cohorts.
- Instead of using a cost-effectiveness threshold, it is possible to determine optimal screening protocols by calculating an acceptability threshold using alternative harms–benefits measures based on existing policy.
- Using univariate harms measures such as primary HPV screening tests or colposcopies per 1,000 people can yield biases in optimizing cervical screening programs. Alternatively, combining both primary screens and colposcopy referrals could provide a more accurate harms measure and result in optimal strategies with a better balance between harms and benefits.

Since 2000, the province of Ontario, Canada, organized cervical screening through introduction of the Ontario Cervical Screening Program (OCSP). The program’s initial recommendation for annual screening with the cytology (Pap) test from age 21 to 70 y and was modified to 3-yearly cytology in 2012.^1^ Preparations to implement primary human papillomavirus (HPV) testing in the OCSP are currently ongoing.^2^

HPV vaccination has been widely accepted as an effective preventive measure against HPV infection,^3,4^ the causal agent of cervical cancer (CC), and is thus expected to prevent this cancer and its precursors. Ontario implemented a publicly funded school-based vaccination program for 13- to 14-y-old (grade 8) girls in 2007.^5^ The program currently covers all students (i.e., of any gender) at age 12 to 13 and uses the nonavalent vaccine, which provides protection against high-risk HPV types 16, 18, 6, 11, 31, 33, 45, 52, and 58.

The first vaccinated individuals have recently started to enter age eligibility for cervical screening. Screening at the intensity of current recommendations is expected to be suboptimal for these cohorts in terms of cost-effectiveness^6,7^ and will likely result in an unfavorable harms–benefits balance of screening. This holds for both the vaccinated individuals as well as to a lesser degree their unvaccinated peers, who experience indirect protection through herd immunity.^8,9^ Considerable gains in screening’s harms–benefits balance can therefore be achieved by adjusting screening recommendations for these vaccinated cohorts to more accurately match their underlying CC risk.

Cost-effectiveness analysis is an important tool for decision making but relies heavily on reliable context-specific cost estimates. In addition, policy makers can feel uncomfortable with valuing different lives and health states in monetary terms and may be hesitant with regard to ethical principles of equitable resource distribution.^10^ These complexities are exacerbated when cost data are missing or highly uncertain. For example, the United States Preventive Services Task Force has based its current guidelines for colorectal cancer^11^ and CC^12^ screening programs on analyses focusing on unadjusted life-years and number of screening tests or examination procedures instead of costs. Similarly, programs that are in the planning stage of significant transition, such as is the case with OCSP, may use other measures to determine screening optimization such as feasibility of implementation and provider and public buy-in among other important considerations.

In this study, we aim, using the microsimulation model STDSIM-MISCAN-Cervix, to determine the harms induced per prevented cancer of 309 different primary HPV screening strategies, varying by screening ages and triage methods. Optimal screening strategies will be determined for both a stratified (by vaccination status) and unstratified analysis based on their ratio of harms to benefits.

## Methods

### Models

To evaluate the most effective cervical screening strategy in different subgroups of vaccinated cohorts in Ontario, we used a hybrid modeling approach, combining 2 well-established microsimulation models for HPV transmission (Sexually Transmitted Diseases Simulation)^13–15^ and CC screening (MISCAN-Cervix),^7,16–18^ respectively. First, STDSIM estimates the effect of vaccination on HPV infections and subsequently this vaccination effect is used as input for the MISCAN-Cervix model in determining screen effects under different scenarios.

STDSIM simulates the transmission and control of sexually transmitted infections. The model simulates the life course of individuals in a dynamic network of sexual contacts. The model was used to determine the impact of vaccination against high-risk HPV infections on type-specific HPV incidence in Ontario, Canada, for the 10 most recently vaccinated birth cohorts (i.e., birth years 1998 to 2007). The Ontario school-based HPV vaccination program was reconstructed as model inputs using vaccination uptake and other details from recent Immunization Coverage Reports.^19^ Catch-up HPV vaccination, for which uptake was low and effectiveness limited for older ages, was disregarded, since the potential herd immunity toward younger routine vaccine-eligible cohorts is expected to be minimal. Future vaccine uptake was assumed to be the average of pre-COVID coverage levels. In the base case, we assumed at 95% lifelong vaccine efficacy for both the quadrivalent and nonavalent vaccines, and we assumed a level of cross-protection for the quadrivalent vaccine of 35% for the other 5 high-risk subtypes.^20^ A dynamic representative sample of the full Ontario population was simulated from the year 2000 up to 2150. The final STDSIM output is the vaccination impact in the 10 vaccine-eligible birth cohorts of interest for both vaccinated and unvaccinated individuals. This includes potential herd immunity effects both within and between birth cohorts.

For estimating the potential effects of different screening strategies, we used the MISCAN-Cervix (Microsimulation Screening Analyses-Cervix) model. MISCAN-Cervix simulates the natural history of CC and the effects of cervical screening in a hypothetical population of individuals who are at risk for acquiring a high-risk HPV infection. These high-risk HPV infections can either clear naturally or progress toward cervical intraepithelial neoplasia (CIN) lesions. The infection has a chance to grow to a CIN1, CIN2, or CIN3 grade lesion with a possibility to regress at any point. If a CIN3 lesion does not regress, it advances toward micro-invasive CC. If left undetected by screening, the cancer can progress to more advanced stages: invasive, regional metastasis, and distant metastasis. From any of these 3 cancer stages, an undetected tumor also had a chance to be clinically detected through symptoms before advancing to the next stage. An extensive model description can be found in the Supplemental Material section 6. Ontario demographic characteristics, all-cause mortality, and hysterectomy rates were obtained from Ontario Health and from Statistics Canada and the Ontario Health Insurance Plan (OHIP) claims database. Durations of high-risk HPV infections^21,22^ and precancerous CIN lesions of grade 1^23^ and 2^23,24^ are drawn from Weibull distributions, whose parameters are taken from the literature. The duration of a CIN3 lesion follows a Weibull with parameters informed by a previous calibration.^18^ Stage-specific survival estimates were incorporated directly from survival data provided by Ontario Health and retrieved from the Ontario Cancer Registry (OCR). Other MISCAN-Cervix model inputs were informed by an Ontario-specific model calibration. Test sensitivity for the cytology test ranges from 12% to 85% based on underlying health state and high- or low-grade results with varying levels of specificity. Test sensitivity for high-grade HPV was assumed 69%, 72%, and 94% for HPV infection, HPV+ CIN1 and HPV+ CIN2+, respectively, with perfect specificity. More details on test characteristics can be found in Supplemental Table S7.

### Calibration

Since no reliable Ontario-specific prevaccination HPV prevalence data were available, we considered alternative sources from Montreal^25^ and British Columbia^26^ as a proxy for the Ontario setting (more details in Supplemental Material section 9). Because a previous quantification of STDSIM for the Netherlands/Dutch setting^15^ was found to accurately replicate these HPV prevalence levels, no recalibration of this part of the model was required.

Some MISCAN-Cervix model parameters could not be obtained directly from observed data or literature sources and were calibrated to fit the Ontario epidemiology. Both cancer stage durations and clinical transition probabilities were calibrated to reproduce current Ontario CC incidence, detection rates, and stage distribution data. Age and type (16, 18, other nonavalent vaccine types [9V], other high risk, low risk) specific HPV infection rates, as well as HPV, CIN1, CIN2, and CIN3 regression probabilities were also adjusted to fit HPV prevalence levels as well as CIN detection rates and CC incidence. The full Ontario population between 2010 and 2017 was simulated for the calibration runs using historical screening attendance behavior across these years.

The target for HPV prevalence was constructed from 2 comparable populations from Montreal^25^ and British Columbia.^26^ Data on calibration targets other than HPV prevalence were provided by Ontario Health. More specifically, CC incidence, mortality, stage distribution, and survival were retrieved from the OCR and CIN and cancer detection rates from a combination of Ontario data sources (CytoBase, OHIP, OCR, Registered Persons Database).

The calibration followed a 3-step iterative process of 1) running the model, 2) evaluating the concordance with observed target data, and 3) changing the unknown model input parameters. A genetic algorithm^27^ was used to determine how to change the input parameters through each cycle to reach a set of parameters that best replicated the calibration targets. The genetic algorithm iteratively evaluates many different parameter sets across multiple generations. The best-fitting sets from each generation are replicated with slight alterations into the next generation, helping the algorithm converge to an optimal fit. A perfect fit on all calibration targets may not always be optimal as it could be a sign of overfitting. For this calibration, we gave preference to a strong fit on overall CC incidence at the expense of an underestimation of cancer detection rates. More details on the calibration process and target fits can be found in Supplemental Material sections 6 and 7.

### Scenarios

A list of all 103 different combinations of screening ages can be found in Supplementary Material section 2. We considered scenarios of 1 to 9 lifetime screens and intervals of 5, 7, 8, 10, 15, and 20 y. No intervals shorter than 5 y were initially considered because 5-yearly screening is widely recommended in the general unvaccinated population, and the lower-risk vaccine-eligible cohorts can be expected to require either similar or less frequent screening. Starting ages ranged from 25 y for all combinations with 5 or more lifetime screens up to 45 y for combinations with 3 lifetime screens. Combinations were excluded if the end age of screening was <45 y for up to 3 lifetime screens or <55 y for 4 or more lifetime screens. No screenings were scheduled after age 70 y in all scenarios. We also considered screening strategies that start with a shorter interval at younger ages and switch to longer intervals at older ages. For example, for 5-yearly screening strategies using primary HPV testing, strategies were added in which the interval is extended to 10 y at older ages for persons with a preceding negative HPV test result.

All strategies considered primary HPV testing, and 3 different follow-up strategies were considered, displayed in detail in Supplemental Material section 3. These strategies were selected as most feasible in collaboration with local stakeholders. The main difference between the strategies is which HPV subtypes are referred directly to colposcopy without a triage test or repeat strategy. In strategy 1, only individuals who tested positive for high-risk HPV subtypes 16 and 18 are referred directly to colposcopy (this aligns with the strategy that will be recommended when primary HPV testing is implemented in Ontario). In strategy 2, individuals with high-risk HPV subtypes covered by the nonavalent vaccine (i.e., types HPV-16/18/31/33/45/52/58) are referred directly to colposcopy. Lastly, in strategy 3, all individuals with a positive HPV test of any high-risk subtype are referred to colposcopy. People with HPV-positive results who are not referred directly to colposcopy in strategy 1 and 2 will have a reflex cytology test (i.e., cytology will be performed on the same specimen automatically by the lab without requiring a new order from the provider); if the cytology result is high grade (high grade squamous intraepithelial lesion [HSIL], atypical squamous cells cannot exclude HSIL [ASC-H], atypical glandular cells [AGC], or adenocarcinoma in situ [AIS]) these individuals will be referred to colposcopy. If the cytology result is negative (negative for intraepithelial lesion malignancy [NILM]) or low-grade (atypical squamous cells of undetermined significance [ASC-US] or low-grade squamous epithelial lesion [LSIL]), a repeat HPV test is recommended in 2 y. Individuals with any high-risk HPV type detected at this repeat test are referred to colposcopy, whereas persons with a negative HPV test return to routine screening (e.g., rescreen at 5 y). These combinations of follow-up strategies and screening ages make up a total of 309 different strategies. The 10 most recent cohorts that were eligible for vaccination in Ontario as part of the school-based program (i.e., birth years 1998 to 2007) have been simulated in MISCAN-Cervix using vaccination effects estimated by STDSIM. All of the different scenarios were then evaluated on this multicohort population. In these analysis runs, we assumed perfect compliance to screening guidelines to prevent biasing findings toward overscreening.^28^

### Analytical Approach

We specifically chose to perform a harms–benefits analysis over a cost-effectiveness analysis based on preferences of local policy makers, because no reliable cost or quality-of-life estimates were available for the Ontario context, particularly costs related to colposcopy. Previous studies have examined many different harms–benefits metrics: Kim et al.^12^ considered colposcopies per life-years gained, screens per life-years gained, and colposcopies per cancer averted; Pedersen et al.^29^ investigated colposcopies per CIN2+ detected; Landy et al.^30^ evaluated screens per CC incidence reduction; and Simms et al.^31^ considered precancer treatments per CC incidence reduction. We therefore considered multiple different outcome measures for harms and benefits of screening. Given the uncertainty as to whether CIN treatments should be considered as a harm (e.g., as in Simms et al.^31^) or a benefit (e.g., Pedersen et al.^29^), we chose to not include treatments as either a harm or a benefit. Thus, our harms measure is a combination of screens and colposcopies. To incorporate the harms of primary screens and colposcopy referrals into a single harms measure, we have used a relative weight of 10 primary screens set as equally harmful as 1 colposcopy referral. This weight of 10 is informed by the average relative quality-adjusted days lost between a primary screen and a false-positive colposcopy as found by a review of disutility weights for cost-effectiveness analyses of cervical screening.^32^ With respect to the uncertainty around these utility weights, we also changed this weight as part of the sensitivity analyses. Finally, the weight of 1:10 also corresponds with the weighted average of the Netherlands/Dutch cost data,^32^ in which a negative colposcopy referral costs about 5 times as much, a CIN detection 15 to 30 times as much, and a screen-detected cancer 100 to 200 times as much as a screen. The total combined harms can be computed as the total colposcopy referrals plus 10 times the number of primary screens. Benefits of screening were defined as the number of cancers prevented as compared with a baseline scenario of no screening. We did not discount either benefits or harms at baseline because we refrained from any monetary evaluation, but included 1.5% and 3% discounting scenarios as sensitivity analyses per CADTH^33^ recommendation.

These definitions allow us to perform a harms–benefits analysis across all 309 scenarios using the efficiency ratio of harms per cancer prevented. In finding efficient strategies, any strategy with more harms and fewer cancers prevented than an alternative strategy is considered dominated as well as any strategy that yields more harms and more cancers prevented but has a higher efficiency ratio than an alternative scenario (i.e., dominated by extension). The remaining, nondominated, screening strategies are selected as the efficient frontier, and incremental harms–benefits ratios (IHBRs)^29^ are computed between them in ascending order of harms and benefits.

An initial analysis of the current unvaccinated population screened with cytology was performed to assess the IHBR of the future HPV-based screening program for the entire current prevaccinated population. The current 3-yearly cytology program was also evaluated in this initial analysis as a comparator. Since this future HPV-based program can be considered as acceptable, we subsequently used this IHBR target as an acceptability threshold, analogous to a willingness-to-pay (WTP) threshold in cost-effectiveness analyses, for selecting the optimal strategies in the main analysis on vaccinated cohorts. The scenario on the efficiency frontier with the highest IHBR below the threshold was selected as optimal for the unstratified scenario and the 2 stratified subgroups.

We repeated the main analysis for multiple different sensitivity analysis scenarios. We examined different assumed vaccine characteristics considering high and low scenarios for both efficacy (90% versus 99%) and cross-protection (0% versus 53%). Also, a scenario with full 1-dose efficacy was explored. A high (90%) and low (56%) scenario was also considered for HPV test sensitivity for high-risk HPV infections and CIN1 lesions. Lastly, in the harms–benefits analysis, we used different harms (only screens or colposcopy referrals) measures, benefits (deaths prevented and life-years gained) measures, and colposcopy weights (5 versus 15) in the combined harms calculation. We also explored a constraint scenario in which only the HPV16/18 genotyping triage is available as well as two discounting scenarios. An overview of the different sensitivity analysis scenarios can be found in Table S1 in the Supplemental Material. Because some of the resulting optimal strategies included the minimal 5-y interval, we performed an additional sensitivity analysis with alternative screening strategies with a shorter interval of 4 y (Supplemental Material Section 12).

This research was funded through a services agreement with Ontario Health. The methods and preliminary results were discussed with the sponsor.

## Results

### Determining the Harms–Benefits Threshold

**Figure 1:**
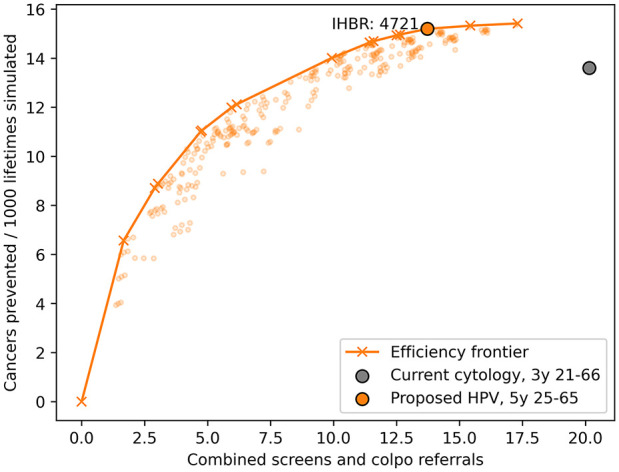
The efficiency frontier of different human papillomavirus (HPV)–based screening strategies in the prevaccinated population. The orange crosses and line represent the efficient frontier. The proposed 9 lifetime HPV tests (orange circle) are on the frontier with an incremental harms–benefits ratio of 4,721 harms per cancer prevented. The cytology-based strategy (gray circle) is clearly shown to be dominated by alternative HPV-based screening strategies. Combined harms = screening tests + 10 × colposcopy referrals. IHBR, incremental harms–benefits ratio.

The efficiency frontier of all different HPV-based screening strategies for the unvaccinated population is illustrated in Figure 1. The proposed primary HPV-based strategy of 9 lifetime screens between the ages of 25 and 65 y in the unvaccinated population is part of this efficiency frontier, with 15.3 cancers prevented per 1,000 lifetimes simulated using 13.7 average harms per lifetime. We can express its efficiency with an IHBR of 4,721 harms per extra cancer prevented compared with its closest less intensive strategy on the frontier. This number will subsequently be used as an acceptability threshold for the upcoming analyses in vaccinated cohorts. The current cytology-based strategy is clearly dominated by primary HPV-based alternatives. The 3-yearly cytology program from age 21 to 66 y prevents almost as many cancers as an alternative 6 lifetime HPV-based screens (i.e., 7-yearly, age 25–60 y) with HPV16/18 triage on the frontier (13.65 against 14.06 per 1,000 lifetimes simulated) but at twice the harms (20.1 v. 9.9).

### Optimal Screening in Vaccinated Cohorts

**Figure 2:**
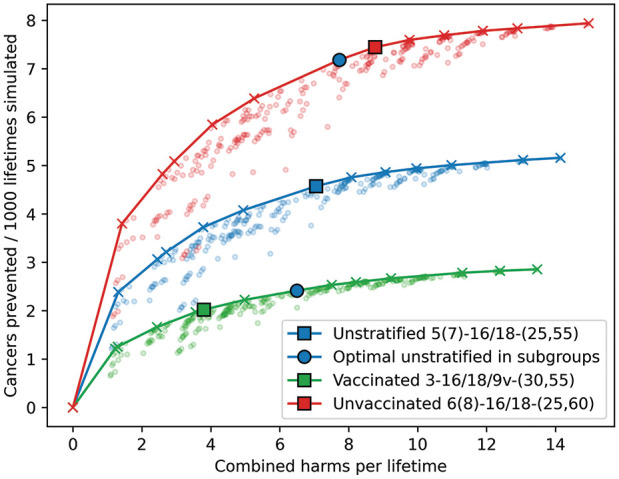
Effectiveness of all evaluated human papillomavirus (HPV)–based screening strategies for preventing cervical cancer incidence in different subgroups of vaccinated cohorts. The combined, unstratified results are displayed in blue with the unvaccinated individuals from these cohorts in red and the vaccinated subgroup in green. Squares represent the optimal strategy using the IHBR threshold of 4,721. The blue circles represent the potential outcome of the optimal unstratified strategy (blue square) in either of the 2 stratified subpopulations. Combined harms = screening tests + 10 × colposcopy referrals. IHBR, incremental harms–benefits ratio. Strategy names should be read as (number of lifetime screens [including optional screens)] – (triage protocol) − (start age, end age).

The outcomes for the vaccine-eligible cohorts are analyzed for the cohort as a whole as well as for the vaccinated and unvaccinated subgroups separately. Each of the 3 subgroups has its own efficiency frontier, as displayed in Figure 2. Using the acceptability threshold of 4,721 harms per cancer prevented, the subgroups all arrive at different levels of optimal screening intensity.

For the unstratified group, the optimal strategy would be 5 lifetime screens with HPV16/18 triage at ages 25, 30, 35, 45, and 55 y with 2 potential additional screens at ages 40 and 50 y following a nonnegative result at ages 35 or 45 y. For the vaccinated subgroup, the optimal strategy would be 3 lifetime screens with HPV16/18/9V triage at ages 30, 40, and 55 y. For the unvaccinated subgroup, the optimal strategy would be 6 lifetime screens with HPV16/18 triage at ages 25, 30, 35, 40, 50, and 60 y along with 2 potential additional screens at ages 45 and 55 y following a nonnegative screening result at ages 40 or 50 y. The vaccinated subgroup results in 3.8 harms per lifetime, the unvaccinated in 8.8 per lifetime, and the optimal amount of harms for the combined unstratified group is 7.1 per lifetime. Similarly, the optimal strategy for the unvaccinated subgroup prevents the most cancers compared with no screening (7.4 per 1,000 people simulated) and the optimal strategy for the vaccinated subgroup the least (2.0 per 1,000 people simulated). In the unvaccinated subpopulation, using the optimal screening strategy results in a CC incidence of 154.7 cancers per 100,000 lifetimes (v. 124.5 in vaccinated and 129.1 in the unstratified subgroup). Further details of all optimal strategies with their respective neighboring strategies on the frontier are given in Table 1. The full efficiency frontiers and full base-case results of all strategies can be found in sections 4 and 5 of the Supplemental Material, respectively.

**Table 1:** Summary Statistics of (Near) Optimal Strategies for Mixed Partially Vaccinated Cohorts, Both Vaccinated and Unvaccinated Individuals in These Cohorts.^a^

Subgroup	Screening Strategy	Total Screens*	Colposcopy Referrals*	Cancers*	Cancer Deaths*	IHBR	AHBR
Unstratified	4-16/18-(35,55)	409,400	8,489	178.1	39.7	3,320	1,213
	**5 (7)-16/18-(25,55)**	**537,937**	**16,767**	**129.1**	**31.7**	**4,310**	**1,546**
	6 (8)-16/18-(25,60)	632,570	17,520	110.4	21.9	5,472	1,700
Vaccinated	3-16/18-(30,55)	306,887	4,911	129.9	33.4	3,715	1,815
	**3-16/18/9v-(30,55)**	**303,038**	**7,699**	**124.5**	**32.4**	**4,432**	**1,886**
	4-16/18/9v-(30,55)	403,757	9,440	104.5	26.4	5,910	2,249
Unvaccinated	5 (7)-16/18-(25,55)	541,381	23,165	181.5	42.3	3,151	1,078
	**6 (8)-16/18-(25,60)**	**635,571**	**24,120**	**154.7**	**29.1**	**3,860**	**1,178**
	7-16/18-(25-61)	726,388	25,002	139.8	23.9	6,713	1,287

AHBR, average harms–benefits ratio; IHBR, incremental harms–benefits ratio; HPV, human papillomavirus.

a Screening strategies are coded as (number of lifetime screens) – (triage method) − (start age, end age). Columns marked with an asterisk (*) present numbers per 100,000 individuals simulated over their entire lifetime. The optimal strategies comparable in efficiency to the future HPV-based strategy are displayed in bold.

The blue circles in Figure 2 represent the outcomes of the optimal unstratified strategy for the 2 subgroups separately. This shows that providing the whole population with a single screening program would move the 2 subpopulations away from their optimal strategies. Even though the optimal unstratified strategy is present on the efficiency frontier for both subgroups, it is not at the optimal level of efficiency. For the vaccinated subgroup, the IHBR moves up from 4,432 to 7,995 harms per cancer prevented, while for the unvaccinated subgroup, it drops from 3,860 to 3,151.

### Sensitivity Analyses

Multiple sensitivity analyses were performed, the results of which are displayed in Table 2. Considering only screens as the sole harms measure did not change the number of advised lifetime screens but shifted all optimal triage strategies to direct colposcopy referral. Only considering colposcopy referrals as harms standardized the optimal screening across the different subgroups to 4 lifetime screens. Changing the weighting of colposcopy in the harms measure up or down to either 5 or 15 did not change the outcomes considerably but affected the optimal triage strategy for the vaccinated subgroup. Using both different benefits measures of deaths prevented or life-years gained resulted in an extra lifetime screen for all subgroups. For deaths prevented, the screening ages also shifted to slightly older ages since CC has worse survivability with age.

**Table 2:** Results of the Sensitivity Analyses.^a^

Scenario	Subgroup	Triage	Lifetime Screens	Screening Ages
Base results	Unstratified	16/18	5 (7)	25, 30, 35, (40,) 45, (50,) 55
	Vaccinated	16/18/9v	3	30, 40, 55
	Unvaccinated	16/18	6 (8)	25, 30, 35, 40, (45,) 50, (55), 60
Screens as sole harms	Unstratified	Direct colpo	**5 (7)**	**25, 30, 35, (40,) 45, (50), 55**
	Vaccinated	Direct colpo	**3**	25, 35, 45
	Unvaccinated	Direct colpo	**6 (8)**	**25, 30, 35, 40, (45,) 50, (55), 60**
Colposcopy weight set to 5 screens	**Unstratified**	**16/18**	**5 (7)**	**25, 30, 35, (40,) 45, (50), 55**
	Vaccinated	Direct colpo	**3**	**30, 40, 55**
	Unvaccinated	**16/18**	**6 (8)**	**25, 30, 35, 40, (45), 50, (55), 60**
Colposcopy weight set to 15 screens	**Unstratified**	**16/18**	**5 (7)**	**25, 30, 35, (40,) 45, (50,) 55**
	Vaccinated	16/18	**3**	**30, 40, 55**
	**Unvaccinated**	**16/18**	**6 (8)**	**25, 30, 35, 40, (45,) 50, (55,) 60**
Colposcopy referrals as sole harms	Unstratified	**16/18**	4	30, 35, 45, 55
	Vaccinated	16/18	4	30, 35, 45, 55
	Unvaccinated	**16/18**	4	30, 35, 45, 55
Deaths prevented as benefits	**Unstratified**	**16/18**	6	25, 33, 41, 49, 56, 64
	Vaccinated	16/18	4	30, 40, 50, 65
	**Unvaccinated**	**16/18**	7	25, 32, 39, 46, 53, 60, 67
Life-years gained as benefits	Unstratified	**16/18**	6 (8)	25, 30, 35, 40, (45), 50, (55), 60
	Vaccinated	**16/18/9v**	4	30, 35, 45, 55
	Unvaccinated	**16/18**	7 (9)	25, 30, 35, 40, 45, (50), 55, (60,) 65
High HPV test sensitivity	Unstratified	16/18	5 (6)	25, 30, 35, 40, (45), 55
	Vaccinated	16/18	**3**	**30, 40, 55**
	**Unvaccinated**	**16/18**	**6 (8)**	**25, 30, 35, 40, (45), 50, (55,) 60**
Low HPV test sensitivity	Unstratified	16/18	5 (6)	25, 30, 35, 40, (45), 55
	**Vaccinated**	**16/18/9v**	**3**	**30, 40, 55**
	**Unvaccinated**	**16/18**	**6 (8)**	**25, 30, 35, 40, (45), 50, (55), 60**
High vaccine efficacy	Unstratified	16/18	5 (6)	25, 30, 35, 40, (45), 55
	Vaccinated	16/18	**3**	**30, 40, 55**
	**Unvaccinated**	**16/18**	**6 (8)**	**25, 30, 35, 40, (45), 50, (55), 60**
Low vaccine efficacy	Unstratified	16/18	6 (8)	25, 30, 35, 40, (45), 50, (55), 60
	Vaccinated	16/18/9v	4	30, 35, 45, 55
	Unvaccinated	16/18	7 (9)	25, 30, 35, 40, 45, (50), 55, (60), 65
Single-dose efficacy	Unstratified	16/18	4 (6)	25, 30, (35), 40, (45), 55
	**Vaccinated**	**16/18/9v**	**3**	**30, 40, 55**
	Unvaccinated	16/18	5 (6)	25, 30, 35, 40, (45), 55
High cross-protection	Unstratified	16/18	5 (6)	25, 30, 35, 40, (45), 55
	Vaccinated	Direct colpo	2	35, 45
	**Unvaccinated**	**16/18**	**6 (8)**	**25, 30, 35, 40, (45), 50, (55), 60**
Low cross-protection	Unstratified	16/18	6 (8)	25, 30, 35, 40, (45), 50, (55), 60
	Vaccinated	16/18	4	25, 35, 45, 60
	Unvaccinated	16/18	7	25, 30, 35, 40, 45, 53, 61
HPV16/18 only	**Unstratified**	**16/18**	**5 (7)**	**25, 30, 35, (40), 45, (50), 55**
	Vaccinated	16/18	4	30, 35, 45, 55
	**Unvaccinated**	**16/18**	**6 (8)**	**25, 30, 35, 40, (45), 50, (55), 60**
3% discounting	Unstratified	**16/18**	5 (6)	25, 30, 35, 40, (45), 55
	**Vaccinated**	**16/18/9v**	**3**	**30, 40, 55**
	Unvaccinated	**16/18**	5 (6)	25, 30, 35, 40, (45), 55
1.5% discounting	**Unstratified**	**16/18**	**5 (7)**	**25, 30, 35, (40), 45, (50), 55**
	**Vaccinated**	**16/18/9v**	**3**	**30, 40, 55**
	**Unvaccinated**	**16/18**	**6 (8)**	**25, 30, 35, 40, (45), 50, (55), 60**

9v, HPV types 31, 33, 45, 52, and 58; direct colpo, direct colposcopy referral for any positive HPV test; HPV, human papillomavirus.

a Optimal combinations of triage method and screening ages for different subgroups and scenarios. Strategies that correspond with the base results are denoted in bold.

Changing the HPV test sensitivity removed only a single optional screen, which does not affect conclusions. By lowering vaccine efficacy, the number of lifetime screens increased by 1 for all subgroups, which can be explained by less vaccine protection. Single-dose efficacy led to a larger part of the population being protected by vaccination, thus increasing the herd immunity for the unvaccinated group and requiring 1 less screen for the unstratified and unvaccinated groups. High cross-protection rates removed 1 lifetime screen from the vaccinated group at the exchange of a more intensive triage strategy of direct colposcopy referral for all HPV subtypes, whereas low cross-protection rates added a lifetime screen for all subgroups. Lastly, if only the HPV16/18 triage strategy is considered, this would add a lifetime screen to the optimal strategy for the vaccinated subgroup. The results were robust to a 1.5% discounting rate, whereas 3% discounting resulted in the removal of a lifetime screen at older ages for the unvaccinated subgroup as well as an optional screen for the unstratified and unvaccinated subgroups. Changing the 5-yearly intervals to 4-yearly intervals for the optimal strategies resulted in less favorable harms–benefits ratios and did not affect the main outcomes (Supplemental Material Section 12).

## Discussion

### Main Findings

We selected specific screening strategies with an optimal harms–benefits balance from the simulated strategies for each of the different vaccine-eligible subgroups. For the unstratified scenario, we found 5 lifetime screens with HPV16/18 genotyping to be optimal. For the stratified scenario, the optimal scenario was 3 lifetime screens with HPV16/18/31/33/45/52/58 genotyping for vaccinated individuals versus 6 lifetime screens with HPV16/18 genotyping for unvaccinated individuals.

### Strengths

We used a harms–benefits analysis to determine optimal screening strategies, while classical cost-effectiveness analyses aim to quantify all aspects of health care decisions into costs and (quality-adjusted) life-years gained. This classical approach allows for decisions to be evaluated against commonly recognized WTP thresholds but does rely on cost and utility estimates that may add uncertainty. Our method both directly assesses the harms and benefits of alternative decisions without adding extra uncertainty and also manages to define an acceptability threshold to guide policy makers. Another strength of this research is the use of 2 state-of-the-art microsimulation models. Combined with close collaboration with experts from Ontario Health, including their data, this allows for a detailed incorporation of the Ontario CC disease dynamics to produce reliable model estimates. In addition, we evaluated a wide range of different screening strategies, thus providing a sufficient number of comparator strategies and an accurate estimation of the IHBR.^34^ Results were generally consistent (i.e., within 1 lifetime screen difference) under all but 1 sensitivity scenario.

### Limitations

A limitation of our study is that we simulated a restricted number of triage strategies and did not consider personalized (e.g., based on previous screening test results) screening strategies, any less intensive triage strategy in which HPV16/18 was not directly referred to colposcopy, nor any new technologies (such as methylation or dual staining). However, the screening and triage strategies included are the ones most feasible in Ontario according to the experts; local factors are important to make the implementation successful. Furthermore, whereas new technologies are under investigation, none are ready to implement in a population-based program.

The decision to follow a harms–benefits framework also has its limitations in disregarding costs and utility estimates. It involves, for example, more subjective judgments in selecting or weighing the various harms and benefits. Most strikingly, if one would disregard the harms of primary screens, our model results would advise against differential screening between vaccinated and unvaccinated subgroups. However, even when using a lower or higher weight for colposcopies in the combined harms measure, we still found that the number of lifetime screens in vaccinated women can be reduced. Also, due to lack of a general acceptable harms–benefits threshold, we assumed the harms–benefits ratio of the currently proposed screening strategy to be acceptable. While this strategy of 5-yearly HPV screening is widely proposed and implemented in many jurisdictions, there is no quantitative precedent to support this assumption for the Ontario context.

It should be mentioned that the calibration fit of the model (see Supplemental Material section 7) was not perfect for all calibration targets. While a perfect fit might not always be preferable as it could lead to overfitting, this does leave room for some potential bias. Most notably, the CIN3 and cancer detection rates in the model were higher than those rates observed in Ontario. This difference may partly be explained by differential data collection methods between detection rates and cancer incidence but could result in a bias toward a stronger effect of screening in the model and subsequently more frequent screening to be found optimal. The model also slightly overestimates HPV prevalence for ages 22 to 24 y, but since no evaluated screening strategies start before age 25 y, this is not expected to have affected our findings. There is still some uncertainty regarding the extent and longevity of the actual cross-protection the quadrivalent HPV vaccine provides against other nonavalent high-risk subtypes.^35^ Our analysis acknowledges this uncertainty by adding high and low cross-protection scenarios to the sensitivity analyses. However, in the unlikely scenario of zero cross-protection, our model reports an extra lifetime screen for all subgroups. If extended follow-up or new research shows the absence of cross-protection or fast waning, screening programs for vaccinated cohorts should be adjusted accordingly.

Another limitation could be that we considered only organized school-based HPV vaccination. We recognize that some individuals may be vaccinated outside of that program, but postexposure and opportunistic vaccination data are not available, nor has vaccine efficacy in reducing cervical disease in those scenarios been well-studied. Next, an acute decrease in vaccination uptake and screening participation was observed during the COVID-19 pandemic. Because the vaccine program uptake is expected to recover in the future, and “catch-up” opportunities are available in the interim, this discrepancy was not factored into the model.

Unfortunately, there was no HPV infection prevalence data from Ontario to inform the model, and we had to resort to neighboring jurisdictions, which could have added some bias in the estimated effectiveness of HPV-based screening. Also, the model did not factor in any migration dynamics. Ontario, and Canada as a whole, experience significant immigration from various backgrounds and diverse, possibly unknown HPV vaccination status, which could affect the level of overall herd immunity. Lastly, immunocompromised individuals, who are generally recommended more intense screening recommendations, were not factored into the model, and results would not generalize to this subpopulation.

### Interpretation

The optimal strategies for vaccinated cohorts in Ontario are in line with those suggested in the existing literature. A review of other modeling work evaluating the cost-effectiveness^6,7,30,36,37^ or harms–benefits balance^30^ of screening strategies in vaccinated and unvaccinated populations found a range of 1 to 5 lifetime screens optimal for vaccinated individuals and a minimum interval of 5-yearly screening for unvaccinated individuals, which would correspond to a maximum of 9 lifetime screens from ages 25 to 65 y and a minimum of 7 lifetime screens.^30^ Our results of 3 lifetime screens for vaccinated individuals correspond very well with these other studies. However, our result of 6 lifetime screens for unvaccinated individuals is lower than the minimum of 7 found in the literature. This difference might be caused by a more detailed estimation of herd immunity effects in our model. Also, our optimal strategy of 6 base lifetime screens also includes 2 optional screens, which could bring the actual lifetime number of tests closer to the 7 from the literature. A previous comparison^38^ of different microsimulation models found MISCAN-Cervix to have comparatively short durations of health states, which might favor more frequent screening. However, the optimal screening strategies found in this study were not more intense than others from the literature.^6,7,30,36,37^

The results show HPV16/18 genotyping to be the optimal triage method for unvaccinated individuals or unstratified screening. For vaccinated individuals, extended HPV16/18/31/33/45/52/58 genotyping was found to be the optimal triage in the base case, although not consistently across all sensitivity scenarios. We hypothesize that the extended genotyping was found optimal among vaccinated individuals as nearly all cancers in that population are from non–vaccine-type HPV infections.

All different sensitivity analyses found differential screening between vaccinated and unvaccinated subgroups optimal, except for when considering only colposcopy referrals as harms. While one might expect lower benefits of an extra screen in a vaccinated population, we suspect the lower HPV prevalence and screen test positivity also decreases the colposcopies, and thereby in this case harms, proportionally. This would allow the same screening strategy to achieve a similar harms–benefits balance in both the vaccinated and unvaccinated subgroups.

To conclude, the results of this modeling exercise demonstrate that the OCSP could consider updating its recommendations for cervical screening in the future for birth cohorts that have been offered HPV vaccination as part of a school-based program. Their lower risk of developing CC justifies a decrease in screening intensity. This finding may be generalized to organized cervical screening programs in jurisdictions with similarly significant coverage of a school-based HPV immunization program. In addition, we suggest that further research be performed examining the feasibility of implementing a program that recommends different screening strategies for vaccinated and nonvaccinated participants. This could be a step forward toward individualized risk-based screening in which every person undergoes a screening intensity appropriate to their harms and benefits tradeoff. Finally, to estimate the impact of screening on the overall health care infrastructure and budget, a more detailed analysis using costs and/or resource constraints should be considered.

## Conclusions

Our modeling results show that a screening strategy with fewer lifetime screens than currently recommended could be offered with an acceptable harms–benefits balance for birth cohorts offered school-based HPV vaccination. To reach these findings, we introduced a combined harms measure that allowed us to compute an acceptability threshold based on the planned future HPV-based screening program in Ontario. The optimal screening protocol for vaccinated cohorts mostly depends on whether the program’s design can be stratified by vaccination status. If this is the case, the OCSP could consider recommending 3 lifetime screens with HPV16/18/9v triage for people with a cervix vaccinated in a school-based program and 6 lifetime screens with HPV16/18 triage and 2 optional screens for others eligible for cervical screening. In the case of no stratification, the optimal screening strategy is 7 lifetime screens with HPV16/18 triage and 2 optional screens.

## Supplemental Material

sj-docx-1-mdm-10.1177_0272989X251332597 – Supplemental material for Optimizing the Harms and Benefits of Cervical Screening in a Partially Vaccinated Population in Ontario, Canada: A Modeling Study Supplemental material, sj-docx-1-mdm-10.1177_0272989X251332597 for Optimizing the Harms and Benefits of Cervical Screening in a Partially Vaccinated Population in Ontario, Canada: A Modeling Study by Daniël D. de Bondt, Erik E. L. Jansen, Christine Stogios, Bronwen R. McCurdy, Rachel Kupets, Joan Murphy, Dustin Costescu, Linda Rabeneck, Rebecca Truscott, Jan A. C. Hontelez and Inge M. C. M. de Kok in Medical Decision Making
