# Supplementary material for: Optimizing the Harms and Benefits of Cervical Screening in a Partially Vaccinated Population in Ontario, Canada: A Modeling Study
Source: Med Decis Making. 2025 Apr 22;45(5):545–56. doi: 10.1177/0272989X251332597 (PMC12166155; doi:10.1177/0272989X251332597)
Supplement: sj-docx-1-mdm-10.1177_0272989X251332597 – Supplemental material for Optimizing the Harms and Benefits of Cervical Screening in a Partially Vaccinated Population in Ontario, Canada: A Modeling Study [file sj-docx-1-mdm-10.1177_0272989X251332597.docx]

**Supplementary Material.**

**CONTENTS**

[1. Sensitivity analyses 2](#_Toc179381793)

[2. Screening ages 2](#_Toc179381794)

[3. Triage strategies 6](#_Toc179381795)

[4. Efficiency frontiers 7](#_Toc179381796)

[5. Full baseline results tables 9](#_Toc179381797)

[6. Model description 38](#_Toc179381798)

[6.1. Model purpose 38](#_Toc179381799)

[6.2. STDSIM model overview 39](#_Toc179381800)

[STDSIM quantifications 42](#_Toc179381801)

[6.3. MISCAN model overview 44](#_Toc179381802)

[6.3.1. Model description 44](#_Toc179381803)

[6.3.2. Demography 45](#_Toc179381804)

[Demography assumptions 45](#_Toc179381805)

[6.3.3. Natural history 45](#_Toc179381806)

[Natural history assumptions 49](#_Toc179381807)

[6.3.4. Screening part 51](#_Toc179381808)

[6.3.5. Screening assumptions 52](#_Toc179381809)

[6.4. Model output 54](#_Toc179381810)

[6.4.1. STDSIM outputs 54](#_Toc179381811)

[6.4.2. Demographic or epidemiological outputs 54](#_Toc179381812)

[6.4.3. Screening outputs 54](#_Toc179381813)

[6.4.4. Harms and Benefits of screening 54](#_Toc179381814)

[6.5. MISCAN model parameters 55](#_Toc179381815)

[6.5.1. Demographic part 55](#_Toc179381816)

[6.5.2. Natural history part 55](#_Toc179381817)

[6.5.3. Screening part 59](#_Toc179381818)

[6.6. Model calibration 62](#_Toc179381819)

[6.6.1. Stepwise process 63](#_Toc179381820)

[7. Calibration results 66](#_Toc179381821)

[8. Summary tables of model data and assumptions. 72](#_Toc179381822)

[9. HPV prevalence sources 74](#_Toc179381823)

[References 75](#_Toc179381824)

# Sensitivity analyses

**Table S1.** Overview of sensitivity analyses. Nonavalent types = HPV 31, 33, 45, 52 and 58; Other high-risk types = high risk HPV types not 16 or 18 and not nonavalent types.

| Model aspect | Parameter | Base case | SA scenario | Value |
| --- | --- | --- | --- | --- |
| Vaccine | Efficacy | 95% | High | 99% |
|  |  |  | Low | 90% |
|  | Cross-protection | Nonavalent types: 35%  Other high-risk types: 0% | High | Nonavalent: 53%  Other high-risk: 20% |
|  |  |  | Low | Both 0% |
|  | Single dose efficacy | 0% | Full efficacy | 95% |
| HPV test | Sensitivity no lesion or CIN1 | 69% (no lesion)  72% (CIN1) | High | 90% |
|  |  |  | Low | 56% |
| Harms-benefits analysis | Colposcopy weight* | 10 | Low  High | 5  15 |
|  | Triage methods | All | Only HPV16/18 genotyping | |
|  | Harms measure | Screens plus weighted colposcopies | Screens only | |
|  |  |  | Colposcopies only | |
|  | Benefits measure | Cancers prevented | Cancer deaths prevented | |
|  |  |  | Life years gained | |
|  | Discounting | No discounting | CADTH | 1.5% |
|  |  |  | High | 3% |

***Colposcopy weight determines the quantification of harms through the following formula: N_harms_ = N_screens_ + weight × N_colposcopy referrals_**

# Screening ages

All combinations of screening ages that were simulated are listed below in Table S2.

The combinations are grouped by number of lifetime screens. Within that group, the combinations are sorted by increasing starting age (and following screening ages).

The screening ages indicated in orange will be skipped after a preceding negative previous HPV test.

**Table S2.** All different screening ages, listed by number of lifetime screens.

1 lifetime screen – 5 combinations

| 1.1 | 30 |
| --- | --- |
| 1-2 | 35 |
| 1-3 | 40 |
| 1-4 | 45 |
| 1-5 | 50 |

2 lifetime screens – 8 combinations

| 2-1 | 30 | 45 |
| --- | --- | --- |
| 2-2 | 30 | 50 |
| 2-3 | 35 | 45 |
| 2-4 | 35 | 50 |
| 2-5 | 35 | 55 |
| 2-6 | 40 | 50 |
| 2-7 | 40 | 55 |
| 2-8 | 40 | 60 |

3 lifetime screens – 21 combinations

| 3-1 | 25 | 35 | 45 |
| --- | --- | --- | --- |
| 3-2 | 25 | 35 | 50 |
| 3-3 | 25 | 35 | 55 |
| 3-4 | 25 | 40 | 55 |
| 3-5 | 25 | 40 | 60 |
| 3-6 | 25 | 45 | 65 |
| 3-7 | 30 | 40 | 50 |
| 3-8 | 30 | 40 | 55 |
| 3-9 | 30 | 40 | 60 |
| 3-10 | 30 | 45 | 60 |
| 3-11 | 30 | 45 | 65 |
| 3-12 | 35 | 45 | 55 |
| 3-13 | 35 | 45 | 60 |
| 3-14 | 35 | 45 | 65 |
| 3-15 | 35 | 50 | 65 |
| 3-16 | 35 | 50 | 70 |
| 3-17 | 40 | 50 | 60 |
| 3-18 | 40 | 50 | 65 |
| 3-19 | 40 | 50 | 70 |
| 3-20 | 45 | 55 | 65 |
| 3-21 | 45 | 55 | 70 |

4 lifetime screens – 16 combinations

| 4-1 | 25 | 35 | 45 | 55 |
| --- | --- | --- | --- | --- |
| 4-2 | 25 | 35 | 45 | 60 |
| 4-3 | 25 | 35 | 45 | 65 |
| 4-4 | 25 | 35 | 50 | 65 |
| 4-5 | 25 | 40 | 55 | 70 |
| 4-6 | 30 | 35 | 45 | 55 |
| 4-7 | 30 | 40 | 50 | 60 |
| 4-8 | 30 | 40 | 50 | 65 |
| 4-9 | 30 | 40 | 50 | 70 |
| 4-10 | 30 | 40 | 55 | 70 |
| 4-11 | 35 | 40 | 45 | 55 |
| 4-12 | 35 | 40 | 48 | 56 |
| 4-13 | 35 | 40 | 50 | 60 |
| 4-14 | 35 | 43 | 51 | 59 |
| 4-15 | 35 | 45 | 55 | 65 |
| 4-16 | 35 | 45 | 55 | 70 |

5 lifetime screens – 4 combinations

| 5-1 | 25 | 30 | 35 | 45 | 55 |
| --- | --- | --- | --- | --- | --- |
| 5-2 | 25 | 30 | 40 | 50 | 60 |
| 5-3 | 25 | 33 | 41 | 49 | 57 |
| 5-4 | 25 | 35 | 45 | 55 | 65 |

6 lifetime screens – 12 combinations

| 6-1 | 25 | 30 | 35 | 40 | 45 | 55 |
| --- | --- | --- | --- | --- | --- | --- |
| 6-2 | 25 | 30 | 35 | 40 | 45 | 55 |
| 6-3 | 25 | 30 | 35 | 40 | 45 | 55 |
| 6-4 | 25 | 30 | 35 | 40 | 50 | 60 |
| 6-5 | 25 | 30 | 35 | 40 | 50 | 60 |
| 6-6 | 25 | 30 | 35 | 45 | 55 | 65 |
| 6-7 | 25 | 30 | 35 | 43 | 51 | 59 |
| 6-8 | 25 | 30 | 37 | 44 | 51 | 58 |
| 6-9 | 25 | 30 | 38 | 46 | 54 | 62 |
| 6-10 | 25 | 30 | 40 | 50 | 60 | 70 |
| 6-11 | 25 | 32 | 39 | 46 | 53 | 60 |
| 6-12 | 25 | 33 | 41 | 49 | 56 | 64 |

7 lifetime screens – 15 combinations

| 7-1 | 25 | 30 | 35 | 40 | 45 | 50 | 55 |
| --- | --- | --- | --- | --- | --- | --- | --- |
| 7-2 | 25 | 30 | 35 | 40 | 45 | 50 | 55 |
| 7-3 | 25 | 30 | 35 | 40 | 45 | 50 | 60 |
| 7-4 | 25 | 30 | 35 | 40 | 45 | 50 | 60 |
| 7-5 | 25 | 30 | 35 | 40 | 45 | 52 | 59 |
| 7-6 | 25 | 30 | 35 | 40 | 45 | 53 | 61 |
| 7-7 | 25 | 30 | 35 | 40 | 45 | 55 | 65 |
| 7-8 | 25 | 30 | 35 | 40 | 45 | 55 | 65 |
| 7-9 | 25 | 30 | 35 | 40 | 47 | 54 | 61 |
| 7-10 | 25 | 30 | 35 | 40 | 48 | 56 | 64 |
| 7-11 | 25 | 30 | 35 | 40 | 50 | 60 | 70 |
| 7-12 | 25 | 30 | 35 | 40 | 50 | 60 | 70 |
| 7-13 | 25 | 30 | 35 | 42 | 49 | 56 | 63 |
| 7-14 | 25 | 30 | 35 | 43 | 51 | 59 | 67 |
| 7-15 | 25 | 32 | 39 | 46 | 53 | 60 | 67 |

8 lifetime screens – 13 combinations

| 8-1 | 25 | 30 | 35 | 40 | 45 | 50 | 55 | 60 |
| --- | --- | --- | --- | --- | --- | --- | --- | --- |
| 8-2 | 25 | 30 | 35 | 40 | 45 | 50 | 55 | 60 |
| 8-3 | 25 | 30 | 35 | 40 | 45 | 50 | 55 | 60 |
| 8-4 | 25 | 30 | 35 | 40 | 45 | 50 | 55 | 60 |
| 8-5 | 25 | 30 | 35 | 40 | 45 | 50 | 55 | 65 |
| 8-6 | 25 | 30 | 35 | 40 | 45 | 50 | 55 | 65 |
| 8-7 | 25 | 30 | 35 | 40 | 45 | 50 | 57 | 64 |
| 8-8 | 25 | 30 | 35 | 40 | 45 | 50 | 58 | 66 |
| 8-9 | 25 | 30 | 35 | 40 | 45 | 50 | 60 | 70 |
| 8-10 | 25 | 30 | 35 | 40 | 45 | 50 | 60 | 70 |
| 8-11 | 25 | 30 | 35 | 40 | 45 | 52 | 59 | 66 |
| 8-12 | 25 | 30 | 35 | 40 | 45 | 53 | 61 | 69 |
| 8-13 | 25 | 30 | 35 | 40 | 47 | 54 | 61 | 68 |

9 lifetime screens – 9 combinations

| 9-1 | 25 | 30 | 35 | 40 | 45 | 50 | 55 | 60 | 65 |
| --- | --- | --- | --- | --- | --- | --- | --- | --- | --- |
| 9-2 | 25 | 30 | 35 | 40 | 45 | 50 | 55 | 60 | 65 |
| 9-3 | 25 | 30 | 35 | 40 | 45 | 50 | 55 | 60 | 65 |
| 9-4 | 25 | 30 | 35 | 40 | 45 | 50 | 55 | 60 | 65 |
| 9-5 | 25 | 30 | 35 | 40 | 45 | 50 | 55 | 60 | 65 |
| 9-6 | 25 | 30 | 35 | 40 | 45 | 50 | 55 | 60 | 70 |
| 9-7 | 25 | 30 | 35 | 40 | 45 | 50 | 55 | 60 | 70 |
| 9-8 | 25 | 30 | 35 | 40 | 45 | 50 | 55 | 60 | 70 |
| 9-9 | 25 | 30 | 35 | 40 | 45 | 50 | 55 | 60 | 70 |

#
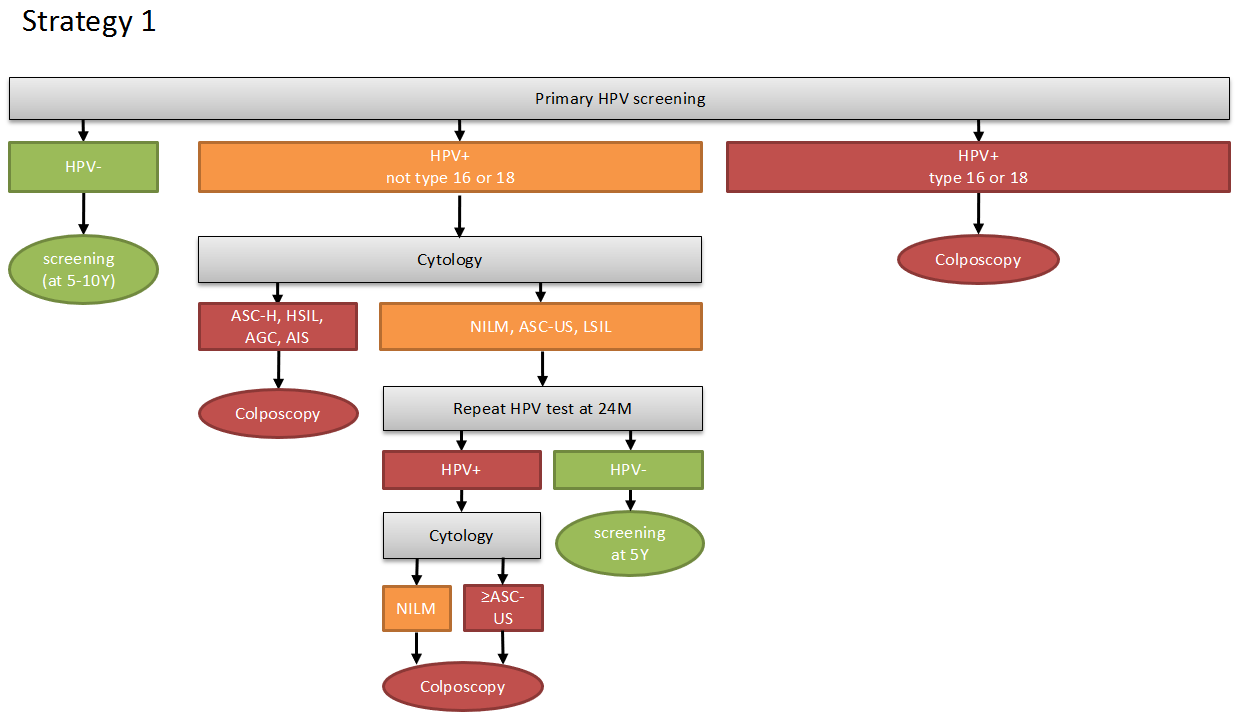
Triage strategies

**
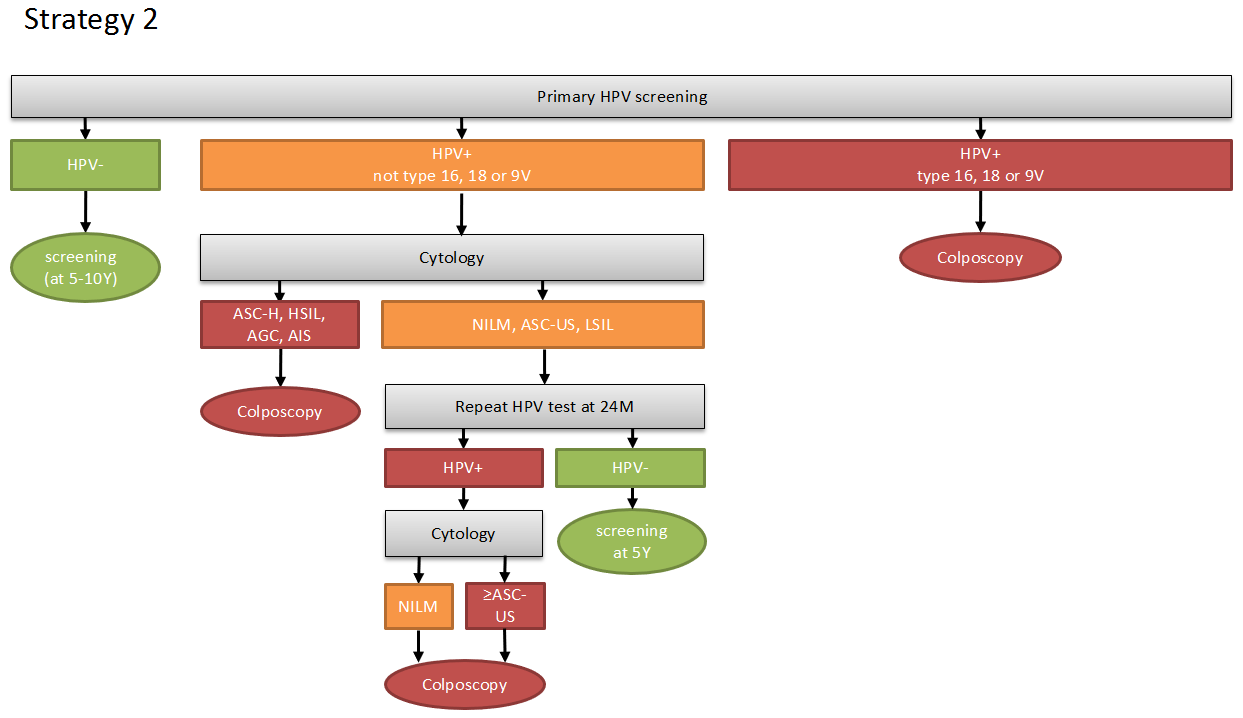
**

**
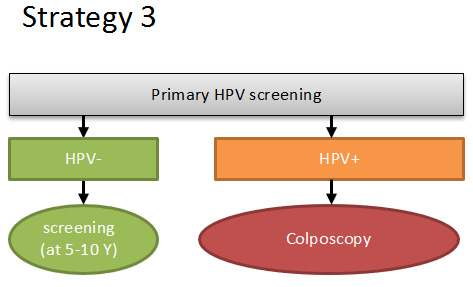
**

**Figure S1. The different triage strategies simulated.**

ASC-H = atypical squamous cells cannot exclude HSIL; HSIL = high grade squamous intraepithelial lesion; AGC = atypical glandular cells; AIS = adenocarcinoma in situ; NILM = negative for intraepithelial lesion or malignancy; ASC-US = atypical squamous cells of undetermined significance; LSIL = low-grade squamous epithelial lesion; 9V = non-16-18 oncogenic HPV types covered by the nonavalent vaccine, types 31, 33, 45, 52 and 58

# Efficiency frontiers

Table S3a. Baseline unstratified. All numbers per 100,000 women simulated. Harms = screens + colposcopy referrals * 10. IHBR = incremental harms-benefits ratio. AHBR = average harms-benefits ratio.

| **Strategy** | **Total screens** | **Colposcopy referrals** | **Harms** | **Cancers** | **Cancers Prevented** | **IHBR** | **AHBR** |
| --- | --- | --- | --- | --- | --- | --- | --- |
| No screening | 0 | 0 | 0 | 586.9 | 0 | NA | NA |
| hpv_16/18_1_2 | 104,081 | 2,751 | 131,595 | 348.6 | 238.3 | 552 | 552 |
| hpv_16/18_2_3 | 204,073 | 4,110 | 245,168 | 280.8 | 306.2 | 1,674 | 801 |
| hpv_16/18_2_1 | 209,917 | 6,059 | 270,511 | 265.8 | 321.2 | 1,690 | 842 |
| hpv_16/18_3_8 | 307,608 | 7,033 | 377,937 | 214.6 | 372.4 | 2,098 | 1,015 |
| hpv_16/18_4_6 | 409,399 | 8,487 | 494,270 | 179.5 | 407.4 | 3,318 | 1,213 |
| **hpv_16/18_7_2** | **537,932** | **16,765** | **705,586** | **129.8** | **457.1** | **4,251** | **1,544** |
| hpv_16/18_8_3 | 632,572 | 17,521 | 807,786 | 111.4 | 475.5 | 5,568 | 1,699 |
| hpv_16/18_7_6 | 724,132 | 18,243 | 906,565 | 100.7 | 486.2 | 9,204 | 1,865 |
| hpv_16/18/9v_7_6 | 709,734 | 28,646 | 996,198 | 93 | 493.9 | 11,624 | 2,017 |
| hpv_16/18/9v_8_7 | 803,694 | 29,439 | 1,098,087 | 86.4 | 500.6 | 15,372 | 2,194 |
| hpv_direct_colpo_8_7 | 771,891 | 53,382 | 1,305,709 | 75.6 | 511.4 | 19,221 | 2,553 |
| hpv_direct_colpo_9_1 | 863,985 | 55,025 | 1,414,235 | 71.1 | 515.8 | 24,544 | 2,742 |

Table S3b. Baseline vaccinated. All numbers per 100,000 women simulated. Harms = screens + colposcopy referrals * 10. IHBR = incremental harms-benefits ratio. AHBR = average harms-benefits ratio.

| **Strategy** | **Total screens** | **Colposcopy referrals** | **Harms** | **Cancers** | **Cancers Prevented** | **IHBR** | **AHBR** |
| --- | --- | --- | --- | --- | --- | --- | --- |
| No screening | 0 | 0 | 0 | 326.9 | 0 | NA | NA |
| hpv_16/18_1_2 | 103,797 | 1,886 | 122,652 | 205.6 | 121.3 | 1,011 | 1,011 |
| hpv_16/18/9v_1_2 | 102,505 | 2,761 | 130,115 | 201.4 | 125.5 | 1,762 | 1,037 |
| hpv_16/18/9v_2_3 | 201,871 | 4,196 | 243,833 | 160.9 | 166 | 2,808 | 1,469 |
| hpv_16/18_3_8 | 306,901 | 4,914 | 356,041 | 130.1 | 196.8 | 3,645 | 1,809 |
| **hpv_16/18/9v_3_8** | **303,051** | **7,699** | **380,037** | **124.9** | **202** | **4,634** | **1,882** |
| hpv_16/18/9v_4_6 | 403,771 | 9,437 | 498,139 | 104.7 | 222.2 | 5,855 | 2,242 |
| hpv_16/18_7_2 | 535,088 | 11,458 | 649,667 | 85.7 | 241.2 | 7,941 | 2,693 |
| hpv_16/18_8_3 | 630,088 | 12,044 | 750,524 | 73.9 | 253 | 8,566 | 2,966 |
| hpv_16/18/9v_8_3 | 619,010 | 20,181 | 820,821 | 68.1 | 258.8 | 12,131 | 3,172 |
| hpv_16/18/9v_9_3 | 712,366 | 21,039 | 922,751 | 60.3 | 266.6 | 13,042 | 3,461 |
| hpv_direct_colpo_9_3 | 681,117 | 44,817 | 1,129,289 | 48.8 | 278.1 | 17,925 | 4,060 |
| hpv_direct_colpo_8_7 | 772,046 | 46,692 | 1,238,963 | 44.7 | 282.2 | 26,739 | 4,390 |
| hpv_direct_colpo_9_1 | 864,162 | 48,247 | 1,346,636 | 41.5 | 285.4 | 34,693 | 4,719 |

Table S3c. Baseline unvaccinated. All numbers per 100,000 women simulated. Harms = screens + colposcopy referrals * 10. IHBR = incremental harms-benefits ratio. AHBR = average harms-benefits ratio.

| **Strategy** | **Total screens** | **Colposcopy referrals** | **Harms** | **Cancers** | **Cancers Prevented** | **IHBR** | **AHBR** |
| --- | --- | --- | --- | --- | --- | --- | --- |
| no_screen | 0 | 0 | 0 | 900 | 0 | NA | NA |
| hpv_16/18_1_2 | 104,422 | 3,793 | 142356 | 520.2 | 379.7 | 375 | 375 |
| hpv_16/18_2_3 | 204,489 | 5,529 | 259774 | 417.7 | 482.3 | 1145 | 539 |
| hpv_16/18_2_1 | 210,708 | 8,358 | 294291 | 391.2 | 508.8 | 1305 | 578 |
| hpv_16/18_3_8 | 308,467 | 9,587 | 404339 | 315.5 | 584.4 | 1454 | 692 |
| hpv_16/18_4_6 | 410,486 | 11,515 | 525637 | 261.5 | 638.5 | 2244 | 823 |
| hpv_16/18_7_2 | 541,375 | 23,162 | 772993 | 181.7 | 718.3 | 3101 | 1076 |
| **hpv_16/18_8_3** | **635,573** | **24,122** | **876792** | **155.6** | **744.4** | **3977** | **1178** |
| hpv_16/18_7_6 | 726,372 | 25,004 | 976416 | 140.5 | 759.5 | 6586 | 1286 |
| hpv_16/18_8_7 | 820,633 | 25,616 | 1076796 | 131.1 | 768.9 | 10648 | 1400 |
| hpv_16/18/9v_8_7 | 802,315 | 38,700 | 1189315 | 121.8 | 778.2 | 12099 | 1528 |
| hpv_16/18/9v_9_1 | 895,766 | 39,327 | 1289036 | 116.6 | 783.4 | 19166 | 1645 |
| hpv_direct_colpo_9_1 | 863,766 | 63,204 | 1495806 | 106.1 | 793.9 | 19737 | 1884 |

# Full baseline results tables

Table S4a. Full baseline results in the unstratified cohort. All numbers per 100,000 individuals simulated. Strategies on the efficiency frontier are highlighted in bold font. Harms = screens + colposcopy referrals * 10

| **Strategy** | **Total screens** | **Colposcopy referrals** | **CIN2/3 treatments** | **Cancers** | **Cancer deaths** | **Harms** | **Cancers prevented** | **Cancer deaths prevented** | **Life years gained** |
| --- | --- | --- | --- | --- | --- | --- | --- | --- | --- |
| current_cyt | 1,618,885 | 27,446 | 6,985 | 131.5 | 21 | 1,893,350 | 455.4 | 250.5 | 6,596 |
| hpv_16/18/9v_1_1 | 106,200 | 7,120 | 1,735 | 367.7 | 160.6 | 177,399 | 219.2 | 110.9 | 3,368 |
| hpv_16/18/9v_1_2 | 102,428 | 3,866 | 1,616 | 343.4 | 126.4 | 141,093 | 243.6 | 145.1 | 4,017 |
| hpv_16/18/9v_1_3 | 100,884 | 2,911 | 1,336 | 373.2 | 119.5 | 129,998 | 213.7 | 152.1 | 3,818 |
| hpv_16/18/9v_1_4 | 99,350 | 2,315 | 1,086 | 418 | 129.7 | 122,503 | 169 | 141.8 | 3,188 |
| hpv_16/18/9v_1_5 | 97,417 | 1,708 | 795 | 461.9 | 150.6 | 114,496 | 125.1 | 120.9 | 2,383 |
| hpv_16/18/9v_2_1 | 205,573 | 9,165 | 2,624 | 260.3 | 76.8 | 297,227 | 326.6 | 194.7 | 5,212 |
| hpv_16/18/9v_2_2 | 203,702 | 8,655 | 2,413 | 282.8 | 82.9 | 290,247 | 304.1 | 188.6 | 4,876 |
| hpv_16/18/9v_2_3 | 201,698 | 5,719 | 2,348 | 273.8 | 76 | 258,888 | 313.2 | 195.6 | 5,098 |
| hpv_16/18/9v_2_4 | 199,868 | 5,285 | 2,210 | 282.7 | 74.4 | 252,713 | 304.3 | 197.2 | 5,005 |
| hpv_16/18/9v_2_5 | 198,075 | 4,902 | 2,029 | 290.2 | 76.3 | 247,100 | 296.7 | 195.2 | 4,829 |
| hpv_16/18/9v_2_6 | 198,218 | 4,208 | 1,833 | 331.9 | 86.7 | 240,300 | 255.1 | 184.9 | 4,420 |
| hpv_16/18/9v_2_7 | 196,453 | 3,877 | 1,698 | 332 | 83.4 | 235,225 | 255 | 188.2 | 4,389 |
| hpv_16/18/9v_2_8 | 194,192 | 3,619 | 1,576 | 337.5 | 83.8 | 230,383 | 249.5 | 187.7 | 4,293 |
| hpv_16/18/9v_3_1 | 313,930 | 19,439 | 3,720 | 192.5 | 59.5 | 508,319 | 394.5 | 212.1 | 5,849 |
| hpv_16/18/9v_3_10 | 298,957 | 9,823 | 2,829 | 231.6 | 50.7 | 397,187 | 355.3 | 220.8 | 5,549 |
| hpv_16/18/9v_3_11 | 295,841 | 9,699 | 2,754 | 239.8 | 53 | 392,835 | 347.2 | 218.6 | 5,472 |
| hpv_16/18/9v_3_12 | 297,306 | 6,596 | 2,638 | 245.9 | 54.6 | 363,263 | 341.1 | 216.9 | 5,416 |
| hpv_16/18/9v_3_13 | 295,067 | 6,373 | 2,550 | 245.7 | 50.7 | 358,798 | 341.2 | 220.8 | 5,423 |
| hpv_16/18/9v_3_14 | 291,951 | 6,251 | 2,477 | 253.7 | 52.6 | 354,463 | 333.3 | 218.9 | 5,353 |
| hpv_16/18/9v_3_15 | 290,096 | 5,790 | 2,321 | 265.1 | 55.6 | 347,997 | 321.9 | 216 | 5,207 |
| hpv_16/18/9v_3_16 | 285,636 | 5,637 | 2,283 | 273.2 | 59.3 | 342,007 | 313.7 | 212.2 | 5,140 |
| hpv_16/18/9v_3_17 | 291,499 | 4,819 | 2,000 | 309.9 | 68.8 | 339,684 | 277 | 202.7 | 4,642 |
| hpv_16/18/9v_3_18 | 288,398 | 4,714 | 1,942 | 314.8 | 68.5 | 335,538 | 272.1 | 203.1 | 4,616 |
| hpv_16/18/9v_3_19 | 283,942 | 4,560 | 1,905 | 322.9 | 72.1 | 329,546 | 264 | 199.5 | 4,550 |
| hpv_16/18/9v_3_2 | 312,099 | 19,007 | 3,585 | 200.1 | 57.2 | 502,169 | 386.9 | 214.4 | 5,775 |
| hpv_16/18/9v_3_20 | 284,927 | 3,676 | 1,472 | 374.5 | 93.3 | 321,688 | 212.4 | 178.3 | 3,684 |
| hpv_16/18/9v_3_21 | 280,485 | 3,535 | 1,444 | 380 | 94.7 | 315,833 | 206.9 | 176.9 | 3,653 |
| hpv_16/18/9v_3_3 | 310,305 | 18,626 | 3,404 | 207.5 | 58.7 | 496,567 | 379.4 | 212.8 | 5,609 |
| hpv_16/18/9v_3_4 | 308,774 | 17,747 | 3,216 | 226.6 | 56.2 | 486,242 | 360.4 | 215.3 | 5,558 |
| hpv_16/18/9v_3_5 | 306,514 | 17,490 | 3,096 | 231.8 | 56.5 | 481,414 | 355.2 | 215.1 | 5,467 |
| hpv_16/18/9v_3_6 | 301,848 | 16,799 | 2,805 | 276 | 68.5 | 469,836 | 310.9 | 203.1 | 4,952 |
| hpv_16/18/9v_3_7 | 304,467 | 10,878 | 3,203 | 209 | 53.1 | 413,243 | 378 | 218.4 | 5,798 |
| hpv_16/18/9v_3_8 | 302,702 | 10,547 | 3,071 | 207.9 | 49 | 408,169 | 379.1 | 222.5 | 5,786 |
| hpv_16/18/9v_3_9 | 300,440 | 10,291 | 2,952 | 212.7 | 48.9 | 403,348 | 374.3 | 222.6 | 5,705 |
| hpv_16/18/9v_4_1 | 409,617 | 20,318 | 4,010 | 164.1 | 38 | 612,802 | 422.8 | 233.5 | 6,171 |
| hpv_16/18/9v_4_10 | 388,521 | 10,882 | 3,131 | 200.7 | 38.4 | 497,343 | 386.2 | 233.1 | 5,876 |
| hpv_16/18/9v_4_11 | 397,792 | 8,397 | 3,019 | 223.8 | 49 | 481,759 | 363.1 | 222.5 | 5,601 |
| hpv_16/18/9v_4_12 | 396,414 | 8,169 | 2,996 | 223.6 | 47.3 | 478,104 | 363.4 | 224.2 | 5,609 |
| hpv_16/18/9v_4_13 | 393,799 | 7,841 | 2,899 | 223.1 | 43.3 | 472,209 | 363.9 | 228.2 | 5,626 |
| hpv_16/18/9v_4_14 | 393,166 | 7,612 | 2,905 | 226.3 | 44.4 | 469,287 | 360.6 | 227.1 | 5,595 |
| hpv_16/18/9v_4_15 | 387,541 | 7,069 | 2,725 | 233.3 | 43 | 458,231 | 353.6 | 228.5 | 5,535 |
| hpv_16/18/9v_4_16 | 383,091 | 6,928 | 2,697 | 238.8 | 44.4 | 452,367 | 348.2 | 227.1 | 5,503 |
| hpv_16/18/9v_4_2 | 407,373 | 20,095 | 3,923 | 163.8 | 33.9 | 608,320 | 423.2 | 237.6 | 6,182 |
| hpv_16/18/9v_4_3 | 404,256 | 19,971 | 3,848 | 172.1 | 36 | 603,965 | 414.9 | 235.6 | 6,107 |
| hpv_16/18/9v_4_4 | 402,401 | 19,515 | 3,697 | 182.7 | 38.4 | 597,547 | 404.3 | 233.1 | 5,977 |
| hpv_16/18/9v_4_5 | 394,573 | 18,077 | 3,276 | 219.2 | 45.6 | 575,344 | 367.8 | 226 | 5,648 |
| hpv_16/18/9v_4_6 | 403,362 | 12,870 | 3,621 | 172.3 | 38.6 | 532,060 | 414.7 | 232.9 | 6,104 |
| hpv_16/18/9v_4_7 | 397,863 | 11,486 | 3,370 | 186.9 | 35.4 | 512,728 | 400.1 | 236.1 | 6,017 |
| hpv_16/18/9v_4_8 | 394,761 | 11,383 | 3,314 | 191.7 | 34.9 | 508,588 | 395.2 | 236.6 | 5,992 |
| hpv_16/18/9v_4_9 | 390,294 | 11,229 | 3,276 | 200.1 | 38.7 | 502,584 | 386.9 | 232.9 | 5,923 |
| hpv_16/18/9v_5_1 | 515,473 | 26,221 | 4,607 | 127.2 | 32.1 | 777,686 | 459.8 | 239.5 | 6,418 |
| hpv_16/18/9v_5_2 | 509,978 | 24,845 | 4,366 | 140.2 | 28.1 | 758,432 | 446.7 | 243.4 | 6,357 |
| hpv_16/18/9v_5_3 | 509,251 | 22,930 | 4,457 | 134.9 | 28.7 | 738,555 | 452 | 242.8 | 6,408 |
| hpv_16/18/9v_5_4 | 499,924 | 20,789 | 4,098 | 151.6 | 26.4 | 707,816 | 435.3 | 245.2 | 6,289 |
| hpv_16/18/9v_6_1 | 616,067 | 28,008 | 4,973 | 107.3 | 27.5 | 896,143 | 479.6 | 244 | 6,562 |
| hpv_16/18/9v_6_10 | 595,834 | 25,156 | 4,411 | 135.6 | 22.5 | 847,390 | 451.4 | 249 | 6,403 |
| hpv_16/18/9v_6_11 | 606,535 | 24,683 | 4,741 | 115.9 | 21.4 | 853,369 | 471 | 250.1 | 6,551 |
| hpv_16/18/9v_6_12 | 600,706 | 23,429 | 4,552 | 124.4 | 20.3 | 834,994 | 462.5 | 251.3 | 6,496 |
| hpv_16/18/9v_6_2 | 520,813 | 26,622 | 4,608 | 124.2 | 33.7 | 787,035 | 462.7 | 237.8 | 6,402 |
| hpv_16/18/9v_6_3 | 431,001 | 24,410 | 4,219 | 150.7 | 39 | 675,103 | 436.3 | 232.6 | 6,214 |
| hpv_16/18/9v_6_4 | 612,067 | 27,453 | 4,855 | 106.6 | 21.9 | 886,601 | 480.4 | 249.7 | 6,587 |
| hpv_16/18/9v_6_5 | 521,884 | 25,235 | 4,465 | 132.9 | 26.8 | 774,231 | 454 | 244.7 | 6,407 |
| hpv_16/18/9v_6_6 | 605,818 | 26,693 | 4,694 | 114.4 | 20.2 | 872,745 | 472.5 | 251.3 | 6,541 |
| hpv_16/18/9v_6_7 | 611,445 | 27,234 | 4,871 | 108.2 | 22.2 | 883,785 | 478.7 | 249.3 | 6,584 |
| hpv_16/18/9v_6_8 | 610,871 | 26,754 | 4,853 | 112.6 | 23.4 | 878,415 | 474.4 | 248.1 | 6,560 |
| hpv_16/18/9v_6_9 | 606,876 | 26,162 | 4,710 | 116.6 | 20.5 | 868,497 | 470.4 | 251 | 6,545 |
| hpv_16/18/9v_7_1 | 713,411 | 28,994 | 5,198 | 100.4 | 25.3 | 1,003,354 | 486.5 | 246.3 | 6,610 |
| hpv_16/18/9v_7_10 | 705,750 | 28,261 | 5,039 | 95.2 | 15.7 | 988,356 | 491.7 | 255.8 | 6,678 |
| hpv_16/18/9v_7_11 | 697,953 | 27,760 | 4,899 | 101.8 | 16.2 | 975,553 | 485.2 | 255.3 | 6,633 |
| hpv_16/18/9v_7_12 | 607,748 | 25,545 | 4,509 | 128.3 | 21 | 863,200 | 458.7 | 250.5 | 6,457 |
| hpv_16/18/9v_7_13 | 705,511 | 28,064 | 5,039 | 96.9 | 16.2 | 986,155 | 490 | 255.4 | 6,668 |
| hpv_16/18/9v_7_14 | 700,224 | 27,649 | 4,926 | 101.2 | 15.6 | 976,712 | 485.7 | 255.9 | 6,644 |
| hpv_16/18/9v_7_15 | 695,295 | 25,090 | 4,790 | 109.9 | 16 | 946,195 | 477 | 255.5 | 6,599 |
| hpv_16/18/9v_7_2 | 524,538 | 26,439 | 4,685 | 122.8 | 30.8 | 788,929 | 464.2 | 240.7 | 6,454 |
| hpv_16/18/9v_7_3 | 711,227 | 28,835 | 5,169 | 95.1 | 18.8 | 999,579 | 491.8 | 252.7 | 6,673 |
| hpv_16/18/9v_7_4 | 526,209 | 25,337 | 4,505 | 130.9 | 26.2 | 779,579 | 456 | 245.3 | 6,424 |
| hpv_16/18/9v_7_5 | 711,095 | 28,785 | 5,164 | 94.2 | 19.2 | 998,942 | 492.7 | 252.4 | 6,674 |
| **hpv_16/18/9v_7_6** | **709,734** | **28,646** | **5,133** | **93** | **17.2** | **996,198** | **493.9** | **254.3** | **6,688** |
| hpv_16/18/9v_7_7 | 706,426 | 28,478 | 5,061 | 94.8 | 15.8 | 991,210 | 492.1 | 255.7 | 6,683 |
| hpv_16/18/9v_7_8 | 611,434 | 26,843 | 4,746 | 111.2 | 19.5 | 879,865 | 475.7 | 252.1 | 6,564 |
| hpv_16/18/9v_7_9 | 708,710 | 28,459 | 5,109 | 94.1 | 17.2 | 993,302 | 492.9 | 254.3 | 6,681 |
| hpv_16/18/9v_8_1 | 806,837 | 29,529 | 5,307 | 88.2 | 16.6 | 1,102,128 | 498.7 | 254.9 | 6,713 |
| hpv_16/18/9v_8_10 | 612,077 | 25,648 | 4,550 | 126.1 | 20.4 | 868,556 | 460.8 | 251.1 | 6,474 |
| hpv_16/18/9v_8_11 | 521,681 | 25,095 | 4,388 | 137.3 | 25.9 | 772,632 | 449.6 | 245.6 | 6,377 |
| hpv_16/18/9v_8_12 | 518,680 | 24,888 | 4,342 | 141.7 | 27.5 | 767,560 | 445.3 | 244 | 6,338 |
| hpv_16/18/9v_8_13 | 796,594 | 28,807 | 5,154 | 89.1 | 12.5 | 1,084,666 | 497.9 | 259 | 6,725 |
| hpv_16/18/9v_8_2 | 713,621 | 28,872 | 5,185 | 94.1 | 18.4 | 1,002,341 | 492.8 | 253.1 | 6,682 |
| hpv_16/18/9v_8_3 | 618,631 | 27,590 | 4,909 | 104.1 | 21 | 894,534 | 482.8 | 250.5 | 6,607 |
| hpv_16/18/9v_8_4 | 528,797 | 25,375 | 4,521 | 130 | 25.9 | 782,544 | 456.9 | 245.7 | 6,432 |
| hpv_16/18/9v_8_5 | 803,774 | 29,464 | 5,284 | 88.4 | 13.9 | 1,098,412 | 498.5 | 257.6 | 6,726 |
| hpv_16/18/9v_8_6 | 614,884 | 26,910 | 4,772 | 110.2 | 19.1 | 883,981 | 476.7 | 252.4 | 6,574 |
| **hpv_16/18/9v_8_7** | **803,694** | **29,439** | **5,276** | **86.4** | **13.5** | **1,098,087** | **500.6** | **258.1** | **6,736** |
| hpv_16/18/9v_8_8 | 801,785 | 29,339 | 5,254 | 87.3 | 13 | 1,095,172 | 499.6 | 258.5 | 6,734 |
| hpv_16/18/9v_8_9 | 797,124 | 29,143 | 5,215 | 90.3 | 13 | 1,088,553 | 496.7 | 258.6 | 6,724 |
| hpv_16/18/9v_9_1 | 897,169 | 29,960 | 5,360 | 82.2 | 11.9 | 1,196,767 | 504.7 | 259.7 | 6,758 |
| hpv_16/18/9v_9_2 | 805,734 | 29,486 | 5,292 | 87.3 | 13.6 | 1,100,598 | 499.6 | 257.9 | 6,731 |
| hpv_16/18/9v_9_3 | 711,706 | 28,565 | 5,094 | 93.1 | 15.2 | 997,361 | 493.8 | 256.3 | 6,693 |
| hpv_16/18/9v_9_4 | 616,966 | 26,932 | 4,780 | 109.4 | 18.8 | 886,282 | 477.6 | 252.7 | 6,579 |
| hpv_16/18/9v_9_6 | 892,737 | 29,836 | 5,352 | 83.5 | 11.2 | 1,191,093 | 503.4 | 260.3 | 6,758 |
| hpv_16/18/9v_9_7 | 799,517 | 29,182 | 5,230 | 89.4 | 12.7 | 1,091,336 | 497.5 | 258.8 | 6,728 |
| hpv_16/18/9v_9_8 | 704,524 | 27,899 | 4,954 | 99.3 | 15.4 | 983,518 | 487.6 | 256.1 | 6,653 |
| hpv_16/18/9v_9_9 | 614,666 | 25,688 | 4,566 | 125.1 | 20.1 | 871,549 | 461.8 | 251.4 | 6,479 |
| hpv_16/18_1_1 | 109,791 | 4,520 | 1,670 | 369.3 | 160 | 154,987 | 217.6 | 111.5 | 3,375 |
| **hpv_16/18_1_2** | **104,081** | **2,751** | **1,540** | **348.6** | **127.4** | **131,595** | **238.3** | **144.1** | **3,978** |
| hpv_16/18_1_3 | 101,996 | 2,190 | 1,270 | 378.7 | 121 | 123,901 | 208.3 | 150.6 | 3,771 |
| hpv_16/18_1_4 | 100,144 | 1,807 | 1,029 | 422.7 | 131.4 | 118,213 | 164.3 | 140.1 | 3,140 |
| hpv_16/18_1_5 | 97,944 | 1,373 | 751 | 465.3 | 152.3 | 111,673 | 121.6 | 119.2 | 2,342 |
| **hpv_16/18_2_1** | **209,917** | **6,059** | **2,507** | **265.8** | **77.9** | **270,511** | **321.2** | **193.6** | **5,176** |
| hpv_16/18_2_2 | 207,795 | 5,723 | 2,308 | 287.2 | 83.9 | 265,024 | 299.7 | 187.6 | 4,843 |
| **hpv_16/18_2_3** | **204,073** | **4,110** | **2,230** | **280.8** | **77.5** | **245,168** | **306.2** | **194** | **5,045** |
| hpv_16/18_2_4 | 202,006 | 3,840 | 2,097 | 289 | 75.7 | 240,402 | 297.9 | 195.9 | 4,960 |
| hpv_16/18_2_5 | 200,021 | 3,585 | 1,928 | 296.7 | 77.7 | 235,871 | 290.2 | 193.9 | 4,782 |
| hpv_16/18_2_6 | 199,792 | 3,165 | 1,737 | 337.9 | 88.2 | 231,444 | 249 | 183.3 | 4,372 |
| hpv_16/18_2_7 | 197,845 | 2,955 | 1,610 | 338.3 | 85.1 | 227,398 | 248.6 | 186.4 | 4,332 |
| hpv_16/18_2_8 | 195,460 | 2,781 | 1,498 | 343.8 | 85.7 | 223,266 | 243.1 | 185.9 | 4,239 |
| hpv_16/18_3_1 | 323,735 | 12,616 | 3,547 | 198.1 | 60.2 | 449,892 | 388.9 | 211.3 | 5,822 |
| hpv_16/18_3_10 | 303,454 | 6,602 | 2,702 | 237.6 | 51.9 | 369,477 | 349.4 | 219.6 | 5,509 |
| hpv_16/18_3_11 | 300,322 | 6,485 | 2,633 | 245.9 | 54.2 | 365,172 | 341 | 217.3 | 5,434 |
| hpv_16/18_3_12 | 299,945 | 4,789 | 2,503 | 253.2 | 56.1 | 347,838 | 333.7 | 215.4 | 5,367 |
| hpv_16/18_3_13 | 297,588 | 4,648 | 2,422 | 253.3 | 52.3 | 344,072 | 333.7 | 219.2 | 5,370 |
| hpv_16/18_3_14 | 294,460 | 4,530 | 2,354 | 261 | 54.3 | 339,758 | 326 | 217.3 | 5,298 |
| hpv_16/18_3_15 | 292,365 | 4,236 | 2,204 | 272.3 | 57.2 | 334,730 | 314.6 | 214.3 | 5,153 |
| hpv_16/18_3_16 | 287,829 | 4,142 | 2,168 | 280.2 | 60.9 | 329,250 | 306.7 | 210.7 | 5,089 |
| hpv_16/18_3_17 | 293,214 | 3,662 | 1,895 | 316.4 | 70.5 | 329,830 | 270.5 | 201.1 | 4,591 |
| hpv_16/18_3_18 | 290,103 | 3,561 | 1,843 | 321.1 | 70 | 325,708 | 265.8 | 201.5 | 4,567 |
| hpv_16/18_3_19 | 285,571 | 3,466 | 1,807 | 329.4 | 73.7 | 320,235 | 257.5 | 197.8 | 4,501 |
| hpv_16/18_3_2 | 321,667 | 12,348 | 3,415 | 205.8 | 58.1 | 445,151 | 381.1 | 213.4 | 5,742 |
| hpv_16/18_3_20 | 286,124 | 2,864 | 1,393 | 379.9 | 95 | 314,765 | 207 | 176.6 | 3,637 |
| hpv_16/18_3_21 | 281,606 | 2,781 | 1,368 | 385.3 | 96.4 | 309,419 | 201.6 | 175.1 | 3,606 |
| hpv_16/18_3_3 | 319,687 | 12,096 | 3,248 | 213.1 | 59.6 | 440,649 | 373.8 | 211.9 | 5,581 |
| hpv_16/18_3_4 | 317,637 | 11,610 | 3,068 | 231.7 | 57.1 | 433,739 | 355.2 | 214.4 | 5,531 |
| hpv_16/18_3_5 | 315,248 | 11,437 | 2,958 | 236.9 | 57.4 | 429,617 | 350 | 214.1 | 5,440 |
| hpv_16/18_3_6 | 310,261 | 10,968 | 2,683 | 279.7 | 69.3 | 419,944 | 307.2 | 202.2 | 4,935 |
| hpv_16/18_3_7 | 309,555 | 7,240 | 3,054 | 215.5 | 54.3 | 381,950 | 371.4 | 217.2 | 5,755 |
| **hpv_16/18_3_8** | **307,608** | **7,033** | **2,930** | **214.6** | **50.1** | **377,937** | **372.4** | **221.4** | **5,747** |
| hpv_16/18_3_9 | 305,223 | 6,860 | 2,820 | 219.4 | 50.1 | 373,824 | 367.5 | 221.5 | 5,666 |
| hpv_16/18_4_1 | 419,689 | 13,295 | 3,820 | 170.7 | 39 | 552,641 | 416.2 | 232.5 | 6,136 |
| hpv_16/18_4_10 | 393,482 | 7,318 | 2,989 | 207.7 | 39.7 | 466,660 | 379.2 | 231.8 | 5,834 |
| hpv_16/18_4_11 | 401,302 | 5,922 | 2,877 | 231.4 | 50.3 | 460,519 | 355.6 | 221.3 | 5,554 |
| hpv_16/18_4_12 | 399,788 | 5,801 | 2,852 | 231.3 | 48.5 | 457,801 | 355.7 | 223 | 5,566 |
| hpv_16/18_4_13 | 396,995 | 5,606 | 2,761 | 230.7 | 44.7 | 453,055 | 356.2 | 226.9 | 5,580 |
| hpv_16/18_4_14 | 396,202 | 5,510 | 2,759 | 234 | 45.8 | 451,301 | 353 | 225.7 | 5,546 |
| hpv_16/18_4_15 | 390,305 | 5,152 | 2,586 | 241.2 | 44.5 | 441,822 | 345.8 | 227.1 | 5,482 |
| hpv_16/18_4_16 | 385,785 | 5,069 | 2,560 | 246 | 45.8 | 436,472 | 340.9 | 225.7 | 5,456 |
| hpv_16/18_4_2 | 417,330 | 13,155 | 3,740 | 170.7 | 35.2 | 548,877 | 416.3 | 236.3 | 6,140 |
| hpv_16/18_4_3 | 414,199 | 13,037 | 3,671 | 178.6 | 37.2 | 544,568 | 408.3 | 234.4 | 6,070 |
| hpv_16/18_4_4 | 412,103 | 12,744 | 3,523 | 188.9 | 39.5 | 539,544 | 398.1 | 232 | 5,946 |
| hpv_16/18_4_5 | 403,492 | 11,895 | 3,128 | 224.7 | 46.6 | 522,441 | 362.3 | 225 | 5,622 |
| **hpv_16/18_4_6** | **409,399** | **8,487** | **3,456** | **179.5** | **39.8** | **494,270** | **407.4** | **231.8** | **6,062** |
| hpv_16/18_4_7 | 403,092 | 7,733 | 3,211 | 194.1 | 36.6 | 480,422 | 392.9 | 234.9 | 5,972 |
| hpv_16/18_4_8 | 399,981 | 7,634 | 3,159 | 198.7 | 36.2 | 476,321 | 388.3 | 235.4 | 5,952 |
| hpv_16/18_4_9 | 395,442 | 7,542 | 3,124 | 206.8 | 39.8 | 470,857 | 380.2 | 231.7 | 5,884 |
| hpv_16/18_5_1 | 528,824 | 16,623 | 4,408 | 134 | 32.9 | 695,052 | 453 | 238.6 | 6,387 |
| hpv_16/18_5_2 | 522,527 | 15,883 | 4,174 | 146.6 | 29.1 | 681,358 | 440.4 | 242.4 | 6,326 |
| hpv_16/18_5_3 | 520,666 | 14,864 | 4,253 | 141.6 | 29.6 | 669,309 | 445.3 | 241.9 | 6,376 |
| hpv_16/18_5_4 | 510,124 | 13,659 | 3,903 | 158.5 | 27.4 | 646,718 | 428.5 | 244.1 | 6,255 |
| hpv_16/18_6_1 | 630,287 | 17,747 | 4,769 | 114.5 | 28.3 | 807,758 | 472.4 | 243.2 | 6,531 |
| hpv_16/18_6_10 | 608,437 | 16,143 | 4,219 | 142 | 23.4 | 769,865 | 445 | 248.1 | 6,373 |
| hpv_16/18_6_11 | 618,762 | 15,984 | 4,525 | 123 | 22.4 | 778,601 | 464 | 249.2 | 6,518 |
| hpv_16/18_6_12 | 612,258 | 15,249 | 4,344 | 131.4 | 21.1 | 764,748 | 455.6 | 250.5 | 6,467 |
| hpv_16/18_6_2 | 534,420 | 16,802 | 4,414 | 131.1 | 34.6 | 702,439 | 455.9 | 237 | 6,371 |
| hpv_16/18_6_3 | 443,395 | 15,488 | 4,018 | 157.9 | 40.1 | 598,278 | 429 | 231.4 | 6,177 |
| hpv_16/18_6_4 | 625,971 | 17,427 | 4,652 | 113.9 | 22.8 | 800,243 | 473 | 248.8 | 6,554 |
| hpv_16/18_6_5 | 534,558 | 16,110 | 4,255 | 140.1 | 27.8 | 695,656 | 446.8 | 243.7 | 6,375 |
| hpv_16/18_6_6 | 619,296 | 16,992 | 4,492 | 121.7 | 21.3 | 789,216 | 465.2 | 250.2 | 6,503 |
| hpv_16/18_6_7 | 625,191 | 17,344 | 4,661 | 115.4 | 23.2 | 798,633 | 471.6 | 248.4 | 6,548 |
| hpv_16/18_6_8 | 624,326 | 17,109 | 4,638 | 119.6 | 24.2 | 795,411 | 467.4 | 247.3 | 6,532 |
| hpv_16/18_6_9 | 619,984 | 16,770 | 4,498 | 124 | 21.6 | 787,681 | 463 | 250 | 6,510 |
| hpv_16/18_7_1 | 728,021 | 18,430 | 4,984 | 107.5 | 26 | 912,324 | 479.5 | 245.5 | 6,580 |
| hpv_16/18_7_10 | 719,957 | 17,996 | 4,828 | 102.8 | 16.6 | 899,916 | 484.2 | 255 | 6,647 |
| hpv_16/18_7_11 | 711,910 | 17,688 | 4,697 | 109.2 | 17.1 | 888,794 | 477.8 | 254.4 | 6,601 |
| hpv_16/18_7_12 | 620,480 | 16,372 | 4,299 | 135.4 | 22.1 | 784,200 | 451.5 | 249.4 | 6,420 |
| hpv_16/18_7_13 | 719,587 | 17,917 | 4,823 | 104.2 | 17.1 | 898,762 | 482.8 | 254.5 | 6,638 |
| hpv_16/18_7_14 | 714,088 | 17,649 | 4,714 | 108.5 | 16.5 | 890,573 | 478.5 | 255 | 6,609 |
| hpv_16/18_7_15 | 707,650 | 16,289 | 4,572 | 117 | 16.9 | 870,538 | 469.9 | 254.6 | 6,570 |
| **hpv_16/18_7_2** | **537,932** | **16,765** | **4,473** | **129.8** | **31.7** | **705,586** | **457.1** | **239.8** | **6,423** |
| hpv_16/18_7_3 | 725,739 | 18,345 | 4,958 | 102.4 | 19.6 | 909,184 | 484.6 | 252 | 6,645 |
| hpv_16/18_7_4 | 538,922 | 16,180 | 4,288 | 138.2 | 27.1 | 700,720 | 448.7 | 244.5 | 6,393 |
| hpv_16/18_7_5 | 725,582 | 18,315 | 4,950 | 101.5 | 20 | 908,735 | 485.5 | 251.5 | 6,640 |
| **hpv_16/18_7_6** | **724,132** | **18,243** | **4,921** | **100.7** | **18.2** | **906,565** | **486.2** | **253.4** | **6,654** |
| hpv_16/18_7_7 | 720,773 | 18,110 | 4,853 | 102.2 | 16.7 | 901,877 | 484.7 | 254.8 | 6,648 |
| hpv_16/18_7_8 | 624,933 | 17,085 | 4,533 | 118.6 | 20.6 | 795,779 | 468.3 | 251 | 6,530 |
| hpv_16/18_7_9 | 722,992 | 18,147 | 4,894 | 101.4 | 18.1 | 904,462 | 485.5 | 253.4 | 6,649 |
| hpv_16/18_8_1 | 821,570 | 18,858 | 5,088 | 95.6 | 17.4 | 1,010,149 | 491.3 | 254.1 | 6,681 |
| hpv_16/18_8_10 | 624,842 | 16,439 | 4,331 | 133.5 | 21.4 | 789,229 | 453.5 | 250.1 | 6,440 |
| hpv_16/18_8_11 | 534,335 | 15,977 | 4,180 | 144.4 | 27.1 | 694,108 | 442.6 | 244.5 | 6,340 |
| hpv_16/18_8_12 | 531,197 | 15,868 | 4,134 | 148.8 | 28.7 | 689,880 | 438.1 | 242.8 | 6,301 |
| hpv_16/18_8_13 | 810,952 | 18,423 | 4,936 | 96.8 | 13.5 | 995,186 | 490.2 | 258 | 6,690 |
| hpv_16/18_8_2 | 728,137 | 18,374 | 4,971 | 101.4 | 19.3 | 911,881 | 485.5 | 252.2 | 6,649 |
| **hpv_16/18_8_3** | **632,572** | **17,521** | **4,696** | **111.4** | **22.1** | **807,786** | **475.5** | **249.5** | **6,571** |
| hpv_16/18_8_4 | 541,528 | 16,208 | 4,302 | 137.1 | 26.9 | 703,606 | 449.8 | 244.7 | 6,397 |
| hpv_16/18_8_5 | 818,512 | 18,792 | 5,067 | 95.8 | 14.8 | 1,006,427 | 491.2 | 256.8 | 6,696 |
| hpv_16/18_8_6 | 628,411 | 17,129 | 4,556 | 117.9 | 20.2 | 799,703 | 469.1 | 251.4 | 6,538 |
| hpv_16/18_8_7 | 818,415 | 18,775 | 5,059 | 93.7 | 14.3 | 1,006,161 | 493.2 | 257.2 | 6,705 |
| hpv_16/18_8_8 | 816,458 | 18,715 | 5,039 | 94.7 | 13.8 | 1,003,613 | 492.3 | 257.7 | 6,704 |
| hpv_16/18_8_9 | 811,689 | 18,607 | 5,000 | 97.5 | 13.9 | 997,755 | 489.4 | 257.6 | 6,690 |
| hpv_16/18_9_1 | 912,024 | 19,176 | 5,138 | 89.6 | 12.8 | 1,103,781 | 497.3 | 258.7 | 6,722 |
| hpv_16/18_9_2 | 820,475 | 18,808 | 5,072 | 95 | 14.5 | 1,008,560 | 492 | 257 | 6,698 |
| hpv_16/18_9_3 | 726,063 | 18,171 | 4,880 | 100.7 | 16.1 | 907,774 | 486.2 | 255.4 | 6,662 |
| hpv_16/18_9_4 | 630,501 | 17,150 | 4,563 | 117.2 | 19.9 | 802,000 | 469.8 | 251.6 | 6,540 |
| hpv_16/18_9_6 | 907,531 | 19,118 | 5,131 | 91.1 | 12.1 | 1,098,713 | 495.9 | 259.4 | 6,725 |
| hpv_16/18_9_7 | 814,085 | 18,633 | 5,014 | 96.7 | 13.6 | 1,000,417 | 490.2 | 257.9 | 6,695 |
| hpv_16/18_9_8 | 718,506 | 17,781 | 4,740 | 106.4 | 16.3 | 896,320 | 480.5 | 255.2 | 6,621 |
| hpv_16/18_9_9 | 627,446 | 16,468 | 4,344 | 132.8 | 21.2 | 792,125 | 454.2 | 250.3 | 6,442 |
| hpv_direct_colpo_1_1 | 99,321 | 12,310 | 1,836 | 366 | 161.3 | 222,425 | 221 | 110.2 | 3,360 |
| hpv_direct_colpo_1_2 | 98,874 | 6,431 | 1,736 | 337.1 | 125.2 | 163,179 | 249.9 | 146.3 | 4,064 |
| hpv_direct_colpo_1_3 | 98,137 | 4,844 | 1,441 | 366.2 | 117.5 | 146,575 | 220.8 | 154 | 3,881 |
| hpv_direct_colpo_1_4 | 96,975 | 3,976 | 1,191 | 411.5 | 127.2 | 136,737 | 175.5 | 144.3 | 3,257 |
| hpv_direct_colpo_1_5 | 95,505 | 3,048 | 893 | 456.8 | 148.3 | 125,982 | 130.2 | 123.2 | 2,439 |
| hpv_direct_colpo_2_1 | 196,369 | 16,016 | 2,824 | 253.5 | 75.4 | 356,532 | 333.5 | 196.1 | 5,259 |
| hpv_direct_colpo_2_2 | 194,938 | 15,184 | 2,609 | 276.9 | 81.7 | 346,781 | 310 | 189.8 | 4,914 |
| hpv_direct_colpo_2_3 | 195,867 | 9,930 | 2,550 | 264.8 | 74.3 | 295,169 | 322.2 | 197.2 | 5,160 |
| hpv_direct_colpo_2_4 | 194,456 | 9,182 | 2,416 | 273.8 | 72.3 | 286,276 | 313.1 | 199.2 | 5,075 |
| hpv_direct_colpo_2_5 | 192,850 | 8,706 | 2,217 | 281.3 | 74.1 | 279,909 | 305.6 | 197.5 | 4,901 |
| hpv_direct_colpo_2_6 | 193,646 | 7,467 | 2,014 | 323.8 | 84.8 | 268,313 | 263.1 | 186.7 | 4,486 |
| hpv_direct_colpo_2_7 | 192,052 | 7,045 | 1,867 | 323.4 | 81.3 | 262,506 | 263.6 | 190.3 | 4,456 |
| hpv_direct_colpo_2_8 | 189,955 | 6,707 | 1,726 | 328 | 81.2 | 257,024 | 259 | 190.3 | 4,367 |
| hpv_direct_colpo_3_1 | 295,549 | 32,816 | 4,023 | 184.8 | 58.4 | 623,713 | 402.2 | 213.1 | 5,887 |
| hpv_direct_colpo_3_10 | 288,270 | 17,826 | 3,071 | 222.5 | 48.8 | 466,534 | 364.4 | 222.7 | 5,606 |
| hpv_direct_colpo_3_11 | 285,317 | 17,618 | 2,981 | 230.6 | 50.7 | 461,492 | 356.3 | 220.8 | 5,534 |
| hpv_direct_colpo_3_12 | 289,847 | 12,038 | 2,897 | 235.7 | 52.9 | 410,226 | 351.2 | 218.6 | 5,479 |
| hpv_direct_colpo_3_13 | 287,757 | 11,737 | 2,795 | 234.5 | 48.4 | 405,125 | 352.4 | 223.1 | 5,496 |
| hpv_direct_colpo_3_14 | 284,805 | 11,528 | 2,706 | 242.2 | 50.1 | 400,081 | 344.8 | 221.4 | 5,424 |
| hpv_direct_colpo_3_15 | 283,375 | 10,752 | 2,553 | 254.3 | 53.1 | 390,900 | 332.6 | 218.5 | 5,280 |
| hpv_direct_colpo_3_16 | 279,074 | 10,506 | 2,504 | 263 | 56.8 | 384,138 | 323.9 | 214.8 | 5,212 |
| hpv_direct_colpo_3_17 | 285,465 | 9,224 | 2,219 | 300.3 | 66.6 | 377,701 | 286.7 | 204.9 | 4,713 |
| hpv_direct_colpo_3_18 | 282,519 | 9,035 | 2,150 | 304.6 | 65.8 | 372,870 | 282.3 | 205.7 | 4,691 |
| hpv_direct_colpo_3_19 | 278,221 | 8,789 | 2,101 | 313.1 | 69.5 | 366,108 | 273.9 | 202.1 | 4,623 |
| hpv_direct_colpo_3_2 | 294,138 | 32,069 | 3,892 | 193.2 | 56 | 614,826 | 393.8 | 215.5 | 5,815 |
| hpv_direct_colpo_3_20 | 279,628 | 7,625 | 1,656 | 365.7 | 90.6 | 355,875 | 221.3 | 180.9 | 3,759 |
| hpv_direct_colpo_3_21 | 275,337 | 7,391 | 1,619 | 371.1 | 92 | 349,249 | 215.9 | 179.6 | 3,726 |
| hpv_direct_colpo_3_3 | 292,532 | 31,597 | 3,693 | 200.2 | 57.4 | 608,504 | 386.7 | 214.1 | 5,650 |
| hpv_direct_colpo_3_4 | 291,786 | 30,078 | 3,491 | 219.7 | 54.9 | 592,564 | 367.2 | 216.6 | 5,597 |
| hpv_direct_colpo_3_5 | 289,687 | 29,740 | 3,351 | 224.2 | 54.7 | 587,086 | 362.7 | 216.8 | 5,512 |
| hpv_direct_colpo_3_6 | 285,551 | 28,685 | 3,044 | 269.2 | 66.6 | 572,402 | 317.8 | 205 | 4,993 |
| hpv_direct_colpo_3_7 | 293,108 | 19,322 | 3,472 | 200.4 | 51.9 | 486,330 | 386.5 | 219.7 | 5,847 |
| hpv_direct_colpo_3_8 | 291,513 | 18,906 | 3,326 | 198.9 | 47.4 | 480,575 | 388 | 224.1 | 5,840 |
| hpv_direct_colpo_3_9 | 289,415 | 18,571 | 3,187 | 203.1 | 46.9 | 475,126 | 383.8 | 224.6 | 5,763 |
| hpv_direct_colpo_4_1 | 389,605 | 34,925 | 4,371 | 155.5 | 36.9 | 738,850 | 431.4 | 234.6 | 6,210 |
| hpv_direct_colpo_4_10 | 376,187 | 20,208 | 3,400 | 190.2 | 36.3 | 578,267 | 396.7 | 235.2 | 5,939 |
| hpv_direct_colpo_4_11 | 387,953 | 15,686 | 3,288 | 213.5 | 47.5 | 544,810 | 373.4 | 224 | 5,657 |
| hpv_direct_colpo_4_12 | 386,787 | 15,285 | 3,279 | 213.2 | 45.7 | 539,632 | 373.8 | 225.9 | 5,672 |
| hpv_direct_colpo_4_13 | 384,455 | 14,763 | 3,176 | 212.4 | 41.6 | 532,084 | 374.5 | 230 | 5,686 |
| hpv_direct_colpo_4_14 | 383,987 | 14,379 | 3,200 | 215 | 42.4 | 527,773 | 371.9 | 229.1 | 5,663 |
| hpv_direct_colpo_4_15 | 378,787 | 13,573 | 3,007 | 221.4 | 40.6 | 514,521 | 365.5 | 231 | 5,610 |
| hpv_direct_colpo_4_16 | 374,489 | 13,337 | 2,970 | 226.9 | 42 | 507,860 | 360 | 229.6 | 5,578 |
| hpv_direct_colpo_4_2 | 387,514 | 34,627 | 4,269 | 154.4 | 32.4 | 733,780 | 432.6 | 239.1 | 6,229 |
| hpv_direct_colpo_4_3 | 384,559 | 34,416 | 4,180 | 162.1 | 34.1 | 728,720 | 424.8 | 237.4 | 6,159 |
| hpv_direct_colpo_4_4 | 383,129 | 33,644 | 4,029 | 173.5 | 36.6 | 719,565 | 413.4 | 235 | 6,026 |
| hpv_direct_colpo_4_5 | 376,441 | 31,380 | 3,564 | 211 | 43.8 | 690,246 | 376 | 227.7 | 5,692 |
| hpv_direct_colpo_4_6 | 389,281 | 23,467 | 3,930 | 162.4 | 37.3 | 623,955 | 424.5 | 234.2 | 6,155 |
| hpv_direct_colpo_4_7 | 385,042 | 21,086 | 3,676 | 176.8 | 33.7 | 595,903 | 410.2 | 237.8 | 6,075 |
| hpv_direct_colpo_4_8 | 382,092 | 20,895 | 3,608 | 181.1 | 33 | 591,047 | 405.8 | 238.6 | 6,049 |
| hpv_direct_colpo_4_9 | 377,789 | 20,651 | 3,559 | 189.7 | 36.6 | 584,295 | 397.2 | 235 | 5,983 |
| hpv_direct_colpo_5_1 | 488,987 | 45,973 | 4,989 | 118.3 | 31.1 | 948,717 | 468.6 | 240.5 | 6,453 |
| hpv_direct_colpo_5_2 | 484,750 | 43,601 | 4,745 | 131.4 | 26.8 | 920,760 | 455.6 | 244.7 | 6,399 |
| hpv_direct_colpo_5_3 | 485,933 | 40,132 | 4,851 | 125.9 | 27.7 | 887,250 | 461 | 243.9 | 6,447 |
| hpv_direct_colpo_5_4 | 478,617 | 36,459 | 4,481 | 141.2 | 24.6 | 843,205 | 445.7 | 246.9 | 6,340 |
| hpv_direct_colpo_6_1 | 587,202 | 49,619 | 5,367 | 98.1 | 26.6 | 1,083,396 | 488.9 | 244.9 | 6,598 |
| hpv_direct_colpo_6_10 | 569,472 | 44,882 | 4,801 | 125.6 | 20.8 | 1,018,295 | 461.3 | 250.7 | 6,451 |
| hpv_direct_colpo_6_11 | 580,686 | 43,870 | 5,166 | 106 | 20.3 | 1,019,388 | 480.9 | 251.3 | 6,592 |
| hpv_direct_colpo_6_12 | 576,046 | 41,723 | 4,969 | 114.3 | 18.8 | 993,273 | 472.7 | 252.7 | 6,541 |
| hpv_direct_colpo_6_2 | 493,990 | 46,732 | 4,980 | 115 | 32.6 | 961,312 | 472 | 238.9 | 6,440 |
| hpv_direct_colpo_6_3 | 406,944 | 42,398 | 4,596 | 141.4 | 37.7 | 830,925 | 445.5 | 233.8 | 6,257 |
| hpv_direct_colpo_6_4 | 583,700 | 48,699 | 5,256 | 96.8 | 20.7 | 1,070,692 | 490.2 | 250.9 | 6,625 |
| hpv_direct_colpo_6_5 | 496,310 | 44,351 | 4,872 | 122.8 | 25.2 | 939,824 | 464.2 | 246.3 | 6,457 |
| hpv_direct_colpo_6_6 | 578,032 | 47,517 | 5,099 | 104 | 18.8 | 1,053,202 | 482.9 | 252.8 | 6,583 |
| hpv_direct_colpo_6_7 | 583,236 | 48,316 | 5,289 | 98.4 | 21 | 1,066,400 | 488.5 | 250.5 | 6,622 |
| hpv_direct_colpo_6_8 | 583,171 | 47,401 | 5,282 | 102.8 | 22.3 | 1,057,177 | 484.1 | 249.2 | 6,600 |
| hpv_direct_colpo_6_9 | 579,605 | 46,504 | 5,130 | 106.5 | 19.2 | 1,044,642 | 480.5 | 252.4 | 6,586 |
| hpv_direct_colpo_7_1 | 682,899 | 51,876 | 5,623 | 91.3 | 24.5 | 1,201,660 | 495.7 | 247 | 6,644 |
| hpv_direct_colpo_7_10 | 675,779 | 50,776 | 5,470 | 84.4 | 14.3 | 1,183,535 | 502.6 | 257.2 | 6,721 |
| hpv_direct_colpo_7_11 | 668,451 | 49,977 | 5,313 | 91.2 | 14.7 | 1,168,217 | 495.7 | 256.9 | 6,677 |
| hpv_direct_colpo_7_12 | 581,039 | 45,632 | 4,928 | 117 | 19.3 | 1,037,361 | 469.9 | 252.3 | 6,506 |
| hpv_direct_colpo_7_13 | 675,690 | 50,443 | 5,481 | 86.3 | 14.9 | 1,180,122 | 500.7 | 256.6 | 6,710 |
| hpv_direct_colpo_7_14 | 670,792 | 49,764 | 5,360 | 90.4 | 14.2 | 1,168,431 | 496.5 | 257.4 | 6,687 |
| hpv_direct_colpo_7_15 | 668,233 | 45,305 | 5,230 | 99.2 | 14.6 | 1,121,283 | 487.7 | 256.9 | 6,642 |
| hpv_direct_colpo_7_2 | 497,857 | 46,420 | 5,101 | 113.5 | 29.9 | 962,059 | 473.5 | 241.7 | 6,492 |
| hpv_direct_colpo_7_3 | 680,809 | 51,663 | 5,595 | 84.8 | 17.5 | 1,197,444 | 502.2 | 254 | 6,715 |
| hpv_direct_colpo_7_4 | 500,522 | 44,559 | 4,929 | 120.7 | 24.7 | 946,117 | 466.3 | 246.8 | 6,472 |
| hpv_direct_colpo_7_5 | 680,696 | 51,593 | 5,594 | 84 | 18.1 | 1,196,623 | 503 | 253.4 | 6,712 |
| hpv_direct_colpo_7_6 | 679,404 | 51,418 | 5,559 | 82.6 | 16 | 1,193,582 | 504.3 | 255.5 | 6,729 |
| hpv_direct_colpo_7_7 | 676,265 | 51,153 | 5,477 | 83.9 | 14.3 | 1,187,797 | 503.1 | 257.2 | 6,725 |
| hpv_direct_colpo_7_8 | 583,534 | 47,806 | 5,170 | 100.3 | 17.9 | 1,061,596 | 486.6 | 253.6 | 6,610 |
| hpv_direct_colpo_7_9 | 678,535 | 51,104 | 5,544 | 83.7 | 16 | 1,189,577 | 503.2 | 255.5 | 6,722 |
| hpv_direct_colpo_8_1 | 774,917 | 53,541 | 5,755 | 78 | 15.7 | 1,310,328 | 508.9 | 255.8 | 6,748 |
| hpv_direct_colpo_8_10 | 585,254 | 45,840 | 4,985 | 114.8 | 18.6 | 1,043,651 | 472.1 | 252.9 | 6,527 |
| hpv_direct_colpo_8_11 | 496,262 | 44,170 | 4,795 | 126.4 | 24.2 | 937,965 | 460.6 | 247.3 | 6,426 |
| hpv_direct_colpo_8_12 | 493,383 | 43,897 | 4,738 | 131.2 | 25.8 | 932,356 | 455.8 | 245.7 | 6,384 |
| hpv_direct_colpo_8_13 | 765,228 | 52,457 | 5,599 | 77.9 | 11.2 | 1,289,802 | 509.1 | 260.3 | 6,763 |
| hpv_direct_colpo_8_2 | 683,159 | 51,758 | 5,619 | 83.7 | 17.2 | 1,200,735 | 503.3 | 254.3 | 6,721 |
| hpv_direct_colpo_8_3 | 590,119 | 48,995 | 5,337 | 93.6 | 19.8 | 1,080,065 | 493.3 | 251.7 | 6,647 |
| hpv_direct_colpo_8_4 | 503,052 | 44,660 | 4,954 | 119.5 | 24.4 | 949,653 | 467.4 | 247.1 | 6,478 |
| hpv_direct_colpo_8_5 | 771,968 | 53,411 | 5,731 | 77.2 | 12.6 | 1,306,080 | 509.7 | 259 | 6,767 |
| hpv_direct_colpo_8_6 | 586,905 | 47,957 | 5,211 | 99.2 | 17.5 | 1,066,472 | 487.8 | 254 | 6,620 |
| **hpv_direct_colpo_8_7** | **771,891** | **53,382** | **5,725** | **75.6** | **12.3** | **1,305,709** | **511.4** | **259.3** | **6,775** |
| hpv_direct_colpo_8_8 | 770,059 | 53,240 | 5,701 | 76.1 | 11.6 | 1,302,457 | 510.8 | 259.9 | 6,776 |
| hpv_direct_colpo_8_9 | 765,573 | 52,940 | 5,651 | 78.9 | 11.5 | 1,294,975 | 508 | 260 | 6,766 |
| **hpv_direct_colpo_9_1** | **863,985** | **55,025** | **5,822** | **71.1** | **10.7** | **1,414,235** | **515.8** | **260.9** | **6,797** |
| hpv_direct_colpo_9_2 | 773,891 | 53,483 | 5,745 | 76.2 | 12.2 | 1,308,718 | 510.7 | 259.3 | 6,773 |
| hpv_direct_colpo_9_3 | 681,437 | 51,373 | 5,531 | 81.8 | 13.7 | 1,195,169 | 505.2 | 257.8 | 6,738 |
| hpv_direct_colpo_9_4 | 588,942 | 48,034 | 5,224 | 98.3 | 17.3 | 1,069,278 | 488.7 | 254.2 | 6,621 |
| hpv_direct_colpo_9_6 | 859,685 | 54,819 | 5,810 | 72.4 | 9.8 | 1,407,872 | 514.5 | 261.7 | 6,798 |
| hpv_direct_colpo_9_7 | 767,923 | 53,036 | 5,675 | 77.9 | 11.3 | 1,298,279 | 509 | 260.3 | 6,771 |
| hpv_direct_colpo_9_8 | 674,872 | 50,273 | 5,393 | 87.9 | 13.8 | 1,177,599 | 499.1 | 257.7 | 6,699 |
| hpv_direct_colpo_9_9 | 587,786 | 45,942 | 5,010 | 113.8 | 18.4 | 1,047,208 | 473.1 | 253.1 | 6,530 |
| no_screen | 0 | 0 | 0 | 586.9 | 271.5 | 0 | 0 | 0 | 0 |

Table S4b. Full baseline results in the vaccinated cohort All numbers per 100,000 individuals simulated. Strategies on the efficiency frontier are highlighted in bold font. Harms = screens + colposcopy referrals * 10

| **Strategy** | **Total screens** | **Colposcopy referrals** | **CIN2/3 treatments** | **Cancers** | **Cancer deaths** | **Harms** | **Cancers prevented** | **Cancer deaths prevented** | **Life years gained** |
| --- | --- | --- | --- | --- | --- | --- | --- | --- | --- |
| current_cyt | 1,612,505 | 25,390 | 5,950 | 76 | 13 | 1,866,408 | 251 | 141 | 3,598 |
| hpv_16/18/9v_1_1 | 106,413 | 5,058 | 1,234 | 217 | 98 | 156,994 | 110 | 56 | 1,691 |
| **hpv_16/18/9v_1_2** | **102,505** | **2,761** | **1,156** | **201** | **78** | **130,115** | **126** | **75** | **2,081** |
| hpv_16/18/9v_1_3 | 100,967 | 2,119 | 967 | 214 | 73 | 122,155 | 113 | 81 | 2,032 |
| hpv_16/18/9v_1_4 | 99,460 | 1,716 | 795 | 236 | 76 | 116,624 | 91 | 77 | 1,737 |
| hpv_16/18/9v_1_5 | 97,547 | 1,306 | 585 | 259 | 87 | 110,609 | 68 | 67 | 1,321 |
| hpv_16/18/9v_2_1 | 205,853 | 6,616 | 1,910 | 157 | 50 | 272,016 | 170 | 104 | 2,762 |
| hpv_16/18/9v_2_2 | 203,987 | 6,268 | 1,752 | 168 | 52 | 266,664 | 159 | 102 | 2,582 |
| **hpv_16/18/9v_2_3** | **201,871** | **4,196** | **1,725** | **161** | **48** | **243,833** | **166** | **106** | **2,729** |
| hpv_16/18/9v_2_4 | 200,034 | 3,899 | 1,618 | 166 | 47 | 239,026 | 161 | 107 | 2,677 |
| hpv_16/18/9v_2_5 | 198,247 | 3,601 | 1,484 | 168 | 47 | 234,255 | 158 | 106 | 2,582 |
| hpv_16/18/9v_2_6 | 198,428 | 3,174 | 1,361 | 189 | 53 | 230,171 | 138 | 101 | 2,401 |
| hpv_16/18/9v_2_7 | 196,663 | 2,913 | 1,261 | 187 | 50 | 225,791 | 139 | 104 | 2,389 |
| hpv_16/18/9v_2_8 | 194,401 | 2,680 | 1,161 | 190 | 49 | 221,203 | 137 | 105 | 2,343 |
| hpv_16/18/9v_3_1 | 314,781 | 14,165 | 2,760 | 120 | 40 | 456,435 | 207 | 114 | 3,101 |
| hpv_16/18/9v_3_10 | 299,321 | 7,145 | 2,079 | 137 | 32 | 370,770 | 190 | 122 | 2,989 |
| hpv_16/18/9v_3_11 | 296,203 | 7,125 | 2,018 | 142 | 33 | 367,457 | 185 | 121 | 2,940 |
| hpv_16/18/9v_3_12 | 297,575 | 4,928 | 1,969 | 142 | 34 | 346,854 | 184 | 120 | 2,941 |
| hpv_16/18/9v_3_13 | 295,328 | 4,721 | 1,893 | 142 | 31 | 342,537 | 185 | 123 | 2,946 |
| hpv_16/18/9v_3_14 | 292,211 | 4,704 | 1,832 | 146 | 32 | 339,255 | 180 | 122 | 2,906 |
| hpv_16/18/9v_3_15 | 290,359 | 4,388 | 1,712 | 153 | 34 | 334,236 | 174 | 120 | 2,820 |
| hpv_16/18/9v_3_16 | 285,889 | 4,224 | 1,681 | 158 | 36 | 328,128 | 169 | 118 | 2,778 |
| hpv_16/18/9v_3_17 | 291,832 | 3,668 | 1,504 | 173 | 40 | 328,513 | 154 | 114 | 2,553 |
| hpv_16/18/9v_3_18 | 288,725 | 3,664 | 1,455 | 176 | 40 | 325,361 | 151 | 114 | 2,539 |
| hpv_16/18/9v_3_19 | 284,259 | 3,498 | 1,424 | 182 | 42 | 319,235 | 145 | 112 | 2,494 |
| hpv_16/18/9v_3_2 | 312,947 | 13,874 | 2,653 | 124 | 38 | 451,688 | 203 | 116 | 3,063 |
| hpv_16/18/9v_3_20 | 285,345 | 2,920 | 1,120 | 206 | 52 | 314,549 | 121 | 102 | 2,068 |
| hpv_16/18/9v_3_21 | 280,890 | 2,767 | 1,098 | 210 | 52 | 308,561 | 117 | 102 | 2,049 |
| hpv_16/18/9v_3_3 | 311,159 | 13,579 | 2,522 | 127 | 38 | 446,945 | 200 | 116 | 2,976 |
| hpv_16/18/9v_3_4 | 309,642 | 12,986 | 2,391 | 135 | 36 | 439,500 | 192 | 118 | 2,964 |
| hpv_16/18/9v_3_5 | 307,379 | 12,750 | 2,290 | 138 | 36 | 434,880 | 189 | 118 | 2,915 |
| hpv_16/18/9v_3_6 | 302,744 | 12,339 | 2,076 | 161 | 41 | 426,138 | 166 | 113 | 2,657 |
| hpv_16/18/9v_3_7 | 304,813 | 7,957 | 2,367 | 127 | 36 | 384,379 | 200 | 118 | 3,095 |
| **hpv_16/18/9v_3_8** | **303,051** | **7,699** | **2,271** | **125** | **32** | **380,037** | **202** | **122** | **3,098** |
| hpv_16/18/9v_3_9 | 300,784 | 7,465 | 2,171 | 127 | 32 | 375,439 | 200 | 122 | 3,053 |
| hpv_16/18/9v_4_1 | 410,529 | 14,904 | 3,002 | 102 | 26 | 559,567 | 225 | 128 | 3,312 |
| hpv_16/18/9v_4_10 | 388,925 | 8,010 | 2,323 | 120 | 24 | 469,030 | 207 | 130 | 3,167 |
| hpv_16/18/9v_4_11 | 398,148 | 6,307 | 2,287 | 129 | 31 | 461,221 | 198 | 123 | 3,046 |
| hpv_16/18/9v_4_12 | 396,762 | 6,148 | 2,267 | 129 | 30 | 458,239 | 198 | 124 | 3,050 |
| hpv_16/18/9v_4_13 | 394,147 | 5,884 | 2,184 | 128 | 27 | 452,989 | 199 | 127 | 3,066 |
| hpv_16/18/9v_4_14 | 393,517 | 5,751 | 2,185 | 130 | 27 | 451,025 | 197 | 127 | 3,055 |
| hpv_16/18/9v_4_15 | 387,893 | 5,394 | 2,045 | 133 | 26 | 441,834 | 194 | 128 | 3,022 |
| hpv_16/18/9v_4_16 | 383,433 | 5,238 | 2,021 | 137 | 27 | 435,812 | 190 | 127 | 3,000 |
| hpv_16/18/9v_4_2 | 408,280 | 14,697 | 2,928 | 101 | 23 | 555,251 | 226 | 131 | 3,324 |
| hpv_16/18/9v_4_3 | 405,159 | 14,672 | 2,866 | 106 | 24 | 551,877 | 221 | 130 | 3,277 |
| hpv_16/18/9v_4_4 | 403,304 | 14,360 | 2,749 | 111 | 25 | 546,909 | 215 | 129 | 3,203 |
| hpv_16/18/9v_4_5 | 395,508 | 13,295 | 2,445 | 130 | 28 | 528,458 | 197 | 125 | 3,031 |
| **hpv_16/18/9v_4_6** | **403,771** | **9,437** | **2,711** | **105** | **26** | **498,139** | **222** | **128** | **3,296** |
| hpv_16/18/9v_4_7 | 398,275 | 8,454 | 2,510 | 112 | 23 | 482,810 | 215 | 131 | 3,250 |
| hpv_16/18/9v_4_8 | 395,169 | 8,450 | 2,463 | 114 | 22 | 479,667 | 213 | 132 | 3,235 |
| hpv_16/18/9v_4_9 | 390,692 | 8,282 | 2,431 | 120 | 25 | 473,513 | 207 | 129 | 3,188 |
| hpv_16/18/9v_5_1 | 516,529 | 19,139 | 3,482 | 82 | 23 | 707,922 | 244 | 131 | 3,448 |
| hpv_16/18/9v_5_2 | 511,038 | 18,160 | 3,288 | 88 | 19 | 692,633 | 239 | 134 | 3,417 |
| hpv_16/18/9v_5_3 | 510,223 | 16,858 | 3,361 | 84 | 20 | 678,801 | 242 | 134 | 3,457 |
| hpv_16/18/9v_5_4 | 500,882 | 15,366 | 3,080 | 92 | 17 | 654,542 | 235 | 137 | 3,398 |
| hpv_16/18/9v_6_1 | 617,159 | 20,508 | 3,790 | 71 | 20 | 822,243 | 256 | 134 | 3,532 |
| hpv_16/18/9v_6_10 | 596,926 | 18,451 | 3,331 | 84 | 15 | 781,437 | 242 | 139 | 3,452 |
| hpv_16/18/9v_6_11 | 607,561 | 18,165 | 3,597 | 73 | 15 | 789,214 | 254 | 139 | 3,544 |
| hpv_16/18/9v_6_12 | 601,720 | 17,336 | 3,445 | 77 | 14 | 775,085 | 250 | 140 | 3,519 |
| hpv_16/18/9v_6_2 | 521,392 | 19,407 | 3,485 | 81 | 24 | 715,458 | 246 | 130 | 3,428 |
| hpv_16/18/9v_6_3 | 429,782 | 17,735 | 3,156 | 96 | 27 | 607,127 | 231 | 127 | 3,312 |
| hpv_16/18/9v_6_4 | 613,148 | 20,087 | 3,687 | 69 | 16 | 814,022 | 257 | 138 | 3,553 |
| hpv_16/18/9v_6_5 | 521,248 | 18,407 | 3,359 | 85 | 19 | 705,319 | 242 | 135 | 3,442 |
| hpv_16/18/9v_6_6 | 606,903 | 19,600 | 3,557 | 73 | 14 | 802,908 | 254 | 140 | 3,534 |
| hpv_16/18/9v_6_7 | 612,530 | 19,962 | 3,697 | 70 | 16 | 812,146 | 257 | 138 | 3,559 |
| hpv_16/18/9v_6_8 | 611,964 | 19,641 | 3,685 | 73 | 17 | 808,378 | 254 | 137 | 3,542 |
| hpv_16/18/9v_6_9 | 607,964 | 19,175 | 3,571 | 74 | 14 | 799,716 | 253 | 140 | 3,536 |
| hpv_16/18/9v_7_1 | 714,530 | 21,329 | 3,980 | 66 | 18 | 927,825 | 261 | 136 | 3,565 |
| hpv_16/18/9v_7_10 | 706,859 | 20,808 | 3,846 | 62 | 11 | 914,941 | 265 | 143 | 3,617 |
| hpv_16/18/9v_7_11 | 699,058 | 20,375 | 3,728 | 65 | 11 | 902,804 | 261 | 143 | 3,588 |
| hpv_16/18/9v_7_12 | 607,147 | 18,697 | 3,398 | 81 | 14 | 794,113 | 246 | 140 | 3,480 |
| hpv_16/18/9v_7_13 | 706,626 | 20,665 | 3,843 | 63 | 12 | 913,272 | 264 | 142 | 3,611 |
| hpv_16/18/9v_7_14 | 701,332 | 20,380 | 3,749 | 64 | 11 | 905,137 | 262 | 143 | 3,603 |
| hpv_16/18/9v_7_15 | 696,352 | 18,578 | 3,641 | 69 | 11 | 882,129 | 258 | 143 | 3,578 |
| hpv_16/18/9v_7_2 | 524,436 | 19,291 | 3,540 | 80 | 22 | 717,346 | 247 | 132 | 3,468 |
| hpv_16/18/9v_7_3 | 712,341 | 21,175 | 3,951 | 63 | 14 | 924,093 | 264 | 140 | 3,610 |
| hpv_16/18/9v_7_4 | 525,023 | 18,478 | 3,388 | 83 | 19 | 709,808 | 243 | 135 | 3,454 |
| hpv_16/18/9v_7_5 | 712,209 | 21,168 | 3,949 | 62 | 14 | 923,886 | 265 | 140 | 3,612 |
| hpv_16/18/9v_7_6 | 710,845 | 21,041 | 3,926 | 61 | 13 | 921,251 | 266 | 141 | 3,620 |
| hpv_16/18/9v_7_7 | 707,539 | 20,969 | 3,866 | 61 | 11 | 917,229 | 265 | 143 | 3,621 |
| hpv_16/18/9v_7_8 | 611,742 | 19,703 | 3,595 | 71 | 13 | 808,770 | 256 | 140 | 3,550 |
| hpv_16/18/9v_7_9 | 709,816 | 20,892 | 3,904 | 61 | 13 | 918,731 | 266 | 141 | 3,613 |
| hpv_16/18/9v_8_1 | 807,981 | 21,760 | 4,075 | 58 | 12 | 1,025,579 | 269 | 142 | 3,638 |
| hpv_16/18/9v_8_10 | 610,922 | 18,773 | 3,428 | 80 | 14 | 798,650 | 247 | 140 | 3,492 |
| hpv_16/18/9v_8_11 | 520,495 | 18,393 | 3,293 | 87 | 18 | 704,425 | 240 | 136 | 3,422 |
| hpv_16/18/9v_8_12 | 517,485 | 18,177 | 3,258 | 90 | 19 | 699,259 | 237 | 135 | 3,402 |
| hpv_16/18/9v_8_13 | 797,726 | 21,237 | 3,946 | 57 | 9 | 1,010,093 | 270 | 145 | 3,651 |
| hpv_16/18/9v_8_2 | 714,551 | 21,205 | 3,965 | 62 | 14 | 926,603 | 265 | 140 | 3,615 |
| **hpv_16/18/9v_8_3** | **619,010** | **20,181** | **3,728** | **68** | **15** | **820,821** | **259** | **139** | **3,563** |
| hpv_16/18/9v_8_4 | 527,392 | 18,509 | 3,399 | 83 | 18 | 712,487 | 244 | 136 | 3,457 |
| hpv_16/18/9v_8_5 | 804,915 | 21,795 | 4,055 | 57 | 10 | 1,022,867 | 270 | 144 | 3,647 |
| hpv_16/18/9v_8_6 | 614,818 | 19,751 | 3,615 | 71 | 13 | 812,327 | 256 | 141 | 3,556 |
| hpv_16/18/9v_8_7 | 804,829 | 21,758 | 4,047 | 56 | 10 | 1,022,412 | 271 | 144 | 3,659 |
| hpv_16/18/9v_8_8 | 802,923 | 21,682 | 4,028 | 56 | 9 | 1,019,747 | 271 | 145 | 3,658 |
| hpv_16/18/9v_8_9 | 798,255 | 21,467 | 3,995 | 59 | 9 | 1,012,922 | 268 | 145 | 3,648 |
| hpv_16/18/9v_9_1 | 898,330 | 22,191 | 4,124 | 53 | 8 | 1,120,242 | 274 | 145 | 3,672 |
| hpv_16/18/9v_9_2 | 806,768 | 21,810 | 4,062 | 57 | 10 | 1,024,869 | 270 | 144 | 3,653 |
| **hpv_16/18/9v_9_3** | **712,366** | **21,039** | **3,892** | **60** | **11** | **922,751** | **267** | **143** | **3,625** |
| hpv_16/18/9v_9_4 | 616,769 | 19,773 | 3,623 | 70 | 13 | 814,500 | 257 | 141 | 3,560 |
| hpv_16/18/9v_9_6 | 893,897 | 22,056 | 4,117 | 54 | 8 | 1,114,456 | 273 | 146 | 3,674 |
| hpv_16/18/9v_9_7 | 800,463 | 21,497 | 4,004 | 58 | 9 | 1,015,431 | 269 | 145 | 3,651 |
| hpv_16/18/9v_9_8 | 704,927 | 20,478 | 3,771 | 64 | 11 | 909,708 | 263 | 143 | 3,598 |
| hpv_16/18/9v_9_9 | 613,292 | 18,807 | 3,442 | 79 | 14 | 801,358 | 248 | 140 | 3,491 |
| hpv_16/18_1_1 | 109,235 | 2,990 | 1,184 | 219 | 98 | 139,136 | 108 | 56 | 1,696 |
| **hpv_16/18_1_2** | **103,797** | **1,886** | **1,096** | **206** | **79** | **122,652** | **121** | **75** | **2,056** |
| hpv_16/18_1_3 | 101,841 | 1,551 | 916 | 218 | 74 | 117,347 | 109 | 80 | 1,999 |
| hpv_16/18_1_4 | 100,084 | 1,316 | 750 | 240 | 78 | 113,245 | 87 | 76 | 1,699 |
| hpv_16/18_1_5 | 97,979 | 1,029 | 551 | 261 | 88 | 108,270 | 66 | 66 | 1,288 |
| hpv_16/18_2_1 | 209,269 | 4,152 | 1,817 | 161 | 50 | 250,785 | 166 | 104 | 2,733 |
| hpv_16/18_2_2 | 207,218 | 3,923 | 1,669 | 172 | 53 | 246,453 | 155 | 100 | 2,546 |
| hpv_16/18_2_3 | 203,729 | 2,931 | 1,633 | 167 | 49 | 233,038 | 160 | 104 | 2,688 |
| hpv_16/18_2_4 | 201,726 | 2,748 | 1,528 | 171 | 48 | 229,201 | 156 | 106 | 2,645 |
| hpv_16/18_2_5 | 199,772 | 2,566 | 1,403 | 173 | 48 | 225,436 | 153 | 106 | 2,550 |
| hpv_16/18_2_6 | 199,681 | 2,339 | 1,287 | 193 | 54 | 223,066 | 134 | 100 | 2,361 |
| hpv_16/18_2_7 | 197,756 | 2,187 | 1,192 | 193 | 51 | 219,629 | 134 | 103 | 2,346 |
| hpv_16/18_2_8 | 195,368 | 2,040 | 1,100 | 195 | 51 | 215,772 | 132 | 103 | 2,295 |
| hpv_16/18_3_1 | 322,627 | 8,577 | 2,620 | 125 | 41 | 408,400 | 202 | 113 | 3,074 |
| hpv_16/18_3_10 | 302,827 | 4,610 | 1,980 | 142 | 33 | 348,929 | 185 | 121 | 2,954 |
| hpv_16/18_3_11 | 299,763 | 4,533 | 1,922 | 147 | 34 | 345,096 | 180 | 120 | 2,908 |
| hpv_16/18_3_12 | 299,640 | 3,510 | 1,862 | 148 | 35 | 334,745 | 179 | 119 | 2,899 |
| hpv_16/18_3_13 | 297,274 | 3,388 | 1,792 | 148 | 33 | 331,153 | 179 | 121 | 2,906 |
| hpv_16/18_3_14 | 294,223 | 3,312 | 1,737 | 152 | 33 | 327,339 | 175 | 121 | 2,864 |
| hpv_16/18_3_15 | 292,199 | 3,114 | 1,621 | 158 | 35 | 323,336 | 169 | 119 | 2,780 |
| hpv_16/18_3_16 | 287,636 | 3,029 | 1,591 | 164 | 37 | 317,922 | 163 | 117 | 2,739 |
| hpv_16/18_3_17 | 293,170 | 2,764 | 1,422 | 178 | 41 | 320,812 | 149 | 113 | 2,516 |
| hpv_16/18_3_18 | 290,130 | 2,700 | 1,377 | 181 | 41 | 317,129 | 146 | 113 | 2,501 |
| hpv_16/18_3_19 | 285,567 | 2,614 | 1,347 | 187 | 43 | 311,710 | 140 | 111 | 2,455 |
| hpv_16/18_3_2 | 320,626 | 8,397 | 2,515 | 129 | 39 | 404,600 | 198 | 115 | 3,034 |
| hpv_16/18_3_20 | 286,334 | 2,243 | 1,058 | 210 | 53 | 308,761 | 116 | 101 | 2,032 |
| hpv_16/18_3_21 | 281,782 | 2,165 | 1,036 | 214 | 54 | 303,436 | 113 | 100 | 2,011 |
| hpv_16/18_3_3 | 318,679 | 8,220 | 2,392 | 132 | 39 | 400,875 | 195 | 115 | 2,944 |
| hpv_16/18_3_4 | 316,757 | 7,930 | 2,268 | 140 | 37 | 396,056 | 187 | 117 | 2,936 |
| hpv_16/18_3_5 | 314,366 | 7,788 | 2,179 | 142 | 37 | 392,242 | 185 | 117 | 2,893 |
| hpv_16/18_3_6 | 309,558 | 7,495 | 1,974 | 164 | 42 | 384,509 | 163 | 112 | 2,635 |
| hpv_16/18_3_7 | 308,825 | 5,061 | 2,252 | 132 | 37 | 359,431 | 195 | 117 | 3,063 |
| **hpv_16/18_3_8** | **306,901** | **4,914** | **2,159** | **130** | **33** | **356,041** | **197** | **121** | **3,067** |
| hpv_16/18_3_9 | 304,511 | 4,766 | 2,067 | 133 | 33 | 352,171 | 194 | 121 | 3,022 |
| hpv_16/18_4_1 | 418,583 | 9,158 | 2,846 | 107 | 27 | 510,162 | 220 | 127 | 3,283 |
| hpv_16/18_4_10 | 392,833 | 5,177 | 2,211 | 125 | 25 | 444,607 | 202 | 129 | 3,129 |
| hpv_16/18_4_11 | 400,894 | 4,362 | 2,175 | 135 | 32 | 444,516 | 192 | 122 | 3,008 |
| hpv_16/18_4_12 | 399,408 | 4,284 | 2,152 | 135 | 31 | 442,249 | 192 | 123 | 3,015 |
| hpv_16/18_4_13 | 396,636 | 4,133 | 2,076 | 134 | 28 | 437,970 | 193 | 126 | 3,032 |
| hpv_16/18_4_14 | 395,899 | 4,099 | 2,073 | 136 | 28 | 436,885 | 191 | 125 | 3,016 |
| hpv_16/18_4_15 | 390,103 | 3,845 | 1,935 | 139 | 27 | 428,557 | 188 | 127 | 2,983 |
| hpv_16/18_4_16 | 385,555 | 3,769 | 1,913 | 143 | 28 | 423,246 | 184 | 126 | 2,963 |
| hpv_16/18_4_2 | 416,213 | 9,036 | 2,779 | 107 | 24 | 506,571 | 220 | 130 | 3,287 |
| hpv_16/18_4_3 | 413,158 | 8,959 | 2,723 | 111 | 25 | 502,752 | 215 | 129 | 3,245 |
| hpv_16/18_4_4 | 411,135 | 8,763 | 2,609 | 117 | 26 | 498,768 | 210 | 128 | 3,173 |
| hpv_16/18_4_5 | 402,675 | 8,195 | 2,321 | 134 | 29 | 484,630 | 193 | 125 | 3,013 |
| hpv_16/18_4_6 | 408,506 | 5,975 | 2,582 | 111 | 27 | 468,254 | 216 | 127 | 3,261 |
| hpv_16/18_4_7 | 402,369 | 5,486 | 2,387 | 117 | 24 | 457,229 | 210 | 130 | 3,212 |
| hpv_16/18_4_8 | 399,330 | 5,422 | 2,343 | 120 | 24 | 453,546 | 207 | 130 | 3,198 |
| hpv_16/18_4_9 | 394,762 | 5,337 | 2,312 | 126 | 26 | 448,137 | 201 | 128 | 3,156 |
| hpv_16/18_5_1 | 527,153 | 11,362 | 3,322 | 88 | 23 | 640,774 | 239 | 131 | 3,424 |
| hpv_16/18_5_2 | 521,030 | 10,884 | 3,135 | 93 | 20 | 629,865 | 234 | 134 | 3,389 |
| hpv_16/18_5_3 | 519,339 | 10,289 | 3,196 | 90 | 21 | 622,234 | 237 | 133 | 3,425 |
| hpv_16/18_5_4 | 509,080 | 9,494 | 2,920 | 98 | 18 | 604,023 | 229 | 136 | 3,368 |
| hpv_16/18_6_1 | 628,459 | 12,212 | 3,627 | 77 | 21 | 750,583 | 250 | 133 | 3,506 |
| hpv_16/18_6_10 | 606,970 | 11,127 | 3,176 | 89 | 16 | 718,238 | 238 | 138 | 3,429 |
| hpv_16/18_6_11 | 617,290 | 11,108 | 3,422 | 79 | 16 | 728,371 | 248 | 138 | 3,514 |
| hpv_16/18_6_12 | 610,976 | 10,640 | 3,277 | 83 | 15 | 717,378 | 244 | 139 | 3,488 |
| hpv_16/18_6_2 | 532,209 | 11,454 | 3,328 | 87 | 25 | 646,744 | 240 | 129 | 3,406 |
| hpv_16/18_6_3 | 439,635 | 10,499 | 2,993 | 102 | 28 | 544,625 | 225 | 126 | 3,279 |
| hpv_16/18_6_4 | 624,195 | 11,978 | 3,526 | 75 | 17 | 743,973 | 252 | 137 | 3,524 |
| hpv_16/18_6_5 | 531,326 | 11,019 | 3,191 | 91 | 20 | 641,516 | 236 | 134 | 3,416 |
| hpv_16/18_6_6 | 617,681 | 11,705 | 3,395 | 79 | 15 | 734,729 | 248 | 139 | 3,505 |
| hpv_16/18_6_7 | 623,465 | 11,951 | 3,531 | 76 | 17 | 742,972 | 251 | 137 | 3,529 |
| hpv_16/18_6_8 | 622,681 | 11,814 | 3,513 | 78 | 18 | 740,819 | 249 | 136 | 3,516 |
| hpv_16/18_6_9 | 618,375 | 11,573 | 3,399 | 80 | 15 | 734,103 | 247 | 139 | 3,505 |
| hpv_16/18_7_1 | 726,157 | 12,780 | 3,809 | 72 | 19 | 853,962 | 255 | 135 | 3,538 |
| hpv_16/18_7_10 | 718,187 | 12,473 | 3,677 | 68 | 12 | 842,913 | 259 | 142 | 3,588 |
| hpv_16/18_7_11 | 710,156 | 12,224 | 3,567 | 71 | 12 | 832,400 | 255 | 142 | 3,564 |
| hpv_16/18_7_12 | 617,283 | 11,263 | 3,231 | 87 | 15 | 729,908 | 240 | 139 | 3,449 |
| hpv_16/18_7_13 | 717,842 | 12,428 | 3,669 | 68 | 12 | 842,120 | 259 | 142 | 3,589 |
| hpv_16/18_7_14 | 712,416 | 12,239 | 3,580 | 70 | 11 | 834,809 | 257 | 142 | 3,576 |
| hpv_16/18_7_15 | 706,235 | 11,400 | 3,466 | 74 | 12 | 820,238 | 252 | 142 | 3,554 |
| **hpv_16/18_7_2** | **535,088** | **11,458** | **3,369** | **86** | **23** | **649,667** | **241** | **131** | **3,441** |
| hpv_16/18_7_3 | 723,860 | 12,704 | 3,783 | 68 | 14 | 850,901 | 259 | 140 | 3,585 |
| hpv_16/18_7_4 | 535,128 | 11,064 | 3,213 | 89 | 19 | 645,770 | 238 | 135 | 3,425 |
| hpv_16/18_7_5 | 723,734 | 12,697 | 3,780 | 68 | 15 | 850,706 | 259 | 139 | 3,584 |
| hpv_16/18_7_6 | 722,274 | 12,636 | 3,755 | 67 | 13 | 848,639 | 260 | 141 | 3,595 |
| hpv_16/18_7_7 | 718,988 | 12,546 | 3,698 | 67 | 12 | 844,445 | 260 | 142 | 3,593 |
| hpv_16/18_7_8 | 622,523 | 11,766 | 3,424 | 77 | 14 | 740,183 | 250 | 140 | 3,522 |
| hpv_16/18_7_9 | 721,150 | 12,569 | 3,731 | 67 | 13 | 846,839 | 260 | 141 | 3,588 |
| hpv_16/18_8_1 | 819,671 | 13,147 | 3,900 | 64 | 13 | 951,141 | 263 | 141 | 3,614 |
| hpv_16/18_8_10 | 621,072 | 11,311 | 3,255 | 85 | 15 | 734,182 | 242 | 139 | 3,463 |
| hpv_16/18_8_11 | 530,639 | 10,934 | 3,128 | 93 | 19 | 639,980 | 234 | 135 | 3,395 |
| hpv_16/18_8_12 | 527,467 | 10,842 | 3,093 | 96 | 20 | 635,885 | 231 | 134 | 3,370 |
| hpv_16/18_8_13 | 809,141 | 12,835 | 3,771 | 63 | 10 | 937,492 | 263 | 144 | 3,621 |
| hpv_16/18_8_2 | 726,076 | 12,730 | 3,795 | 68 | 14 | 853,377 | 259 | 140 | 3,585 |
| **hpv_16/18_8_3** | **630,088** | **12,044** | **3,558** | **74** | **16** | **750,524** | **253** | **138** | **3,534** |
| hpv_16/18_8_4 | 537,505 | 11,086 | 3,224 | 89 | 19 | 648,365 | 238 | 135 | 3,424 |
| hpv_16/18_8_5 | 816,680 | 13,114 | 3,882 | 63 | 11 | 947,819 | 264 | 143 | 3,623 |
| hpv_16/18_8_6 | 625,616 | 11,796 | 3,441 | 77 | 14 | 743,574 | 250 | 140 | 3,524 |
| hpv_16/18_8_7 | 816,574 | 13,095 | 3,874 | 62 | 10 | 947,523 | 265 | 144 | 3,633 |
| hpv_16/18_8_8 | 814,635 | 13,054 | 3,857 | 62 | 10 | 945,174 | 264 | 144 | 3,630 |
| hpv_16/18_8_9 | 809,833 | 12,952 | 3,823 | 65 | 10 | 939,349 | 262 | 144 | 3,621 |
| hpv_16/18_9_1 | 910,169 | 13,451 | 3,946 | 59 | 9 | 1,044,675 | 268 | 145 | 3,641 |
| hpv_16/18_9_2 | 818,539 | 13,131 | 3,886 | 63 | 11 | 949,850 | 264 | 143 | 3,622 |
| hpv_16/18_9_3 | 723,821 | 12,594 | 3,720 | 66 | 12 | 849,758 | 261 | 142 | 3,602 |
| hpv_16/18_9_4 | 627,568 | 11,812 | 3,447 | 76 | 14 | 745,689 | 251 | 140 | 3,525 |
| hpv_16/18_9_6 | 905,651 | 13,397 | 3,939 | 60 | 9 | 1,039,618 | 267 | 145 | 3,643 |
| hpv_16/18_9_7 | 812,038 | 12,973 | 3,834 | 64 | 10 | 941,767 | 263 | 144 | 3,623 |
| hpv_16/18_9_8 | 716,039 | 12,295 | 3,600 | 70 | 12 | 838,985 | 257 | 142 | 3,575 |
| hpv_16/18_9_9 | 623,455 | 11,337 | 3,265 | 85 | 15 | 736,826 | 242 | 139 | 3,464 |
| hpv_direct_colpo_1_1 | 99,330 | 10,451 | 1,341 | 215 | 98 | 203,835 | 112 | 56 | 1,694 |
| hpv_direct_colpo_1_2 | 98,901 | 5,373 | 1,277 | 194 | 77 | 152,634 | 132 | 77 | 2,135 |
| hpv_direct_colpo_1_3 | 98,191 | 4,075 | 1,073 | 207 | 71 | 138,945 | 120 | 83 | 2,094 |
| hpv_direct_colpo_1_4 | 97,063 | 3,397 | 901 | 229 | 74 | 131,029 | 98 | 80 | 1,811 |
| hpv_direct_colpo_1_5 | 95,625 | 2,655 | 683 | 254 | 85 | 122,175 | 73 | 69 | 1,376 |
| hpv_direct_colpo_2_1 | 196,430 | 13,684 | 2,115 | 149 | 48 | 333,269 | 178 | 106 | 2,813 |
| hpv_direct_colpo_2_2 | 195,012 | 13,003 | 1,952 | 162 | 51 | 325,043 | 165 | 103 | 2,617 |
| hpv_direct_colpo_2_3 | 195,974 | 8,467 | 1,928 | 151 | 47 | 280,645 | 176 | 107 | 2,795 |
| hpv_direct_colpo_2_4 | 194,567 | 7,850 | 1,826 | 156 | 45 | 273,066 | 171 | 109 | 2,751 |
| hpv_direct_colpo_2_5 | 192,966 | 7,458 | 1,673 | 159 | 45 | 267,544 | 168 | 109 | 2,659 |
| hpv_direct_colpo_2_6 | 193,819 | 6,465 | 1,545 | 181 | 51 | 258,466 | 146 | 103 | 2,463 |
| hpv_direct_colpo_2_7 | 192,225 | 6,115 | 1,431 | 179 | 48 | 253,379 | 148 | 106 | 2,458 |
| hpv_direct_colpo_2_8 | 190,130 | 5,800 | 1,313 | 181 | 47 | 248,134 | 146 | 107 | 2,417 |
| hpv_direct_colpo_3_1 | 295,611 | 28,328 | 3,074 | 112 | 39 | 578,891 | 215 | 115 | 3,143 |
| hpv_direct_colpo_3_10 | 288,409 | 15,370 | 2,327 | 127 | 30 | 442,106 | 200 | 124 | 3,052 |
| hpv_direct_colpo_3_11 | 285,453 | 15,259 | 2,250 | 132 | 31 | 438,044 | 195 | 123 | 3,007 |
| hpv_direct_colpo_3_12 | 290,041 | 10,439 | 2,230 | 132 | 32 | 394,435 | 195 | 121 | 3,003 |
| hpv_direct_colpo_3_13 | 287,950 | 10,152 | 2,140 | 130 | 29 | 389,467 | 197 | 125 | 3,023 |
| hpv_direct_colpo_3_14 | 284,995 | 10,044 | 2,064 | 135 | 30 | 385,430 | 192 | 124 | 2,980 |
| hpv_direct_colpo_3_15 | 283,577 | 9,407 | 1,947 | 142 | 31 | 377,643 | 185 | 123 | 2,900 |
| hpv_direct_colpo_3_16 | 279,269 | 9,149 | 1,904 | 148 | 33 | 370,757 | 179 | 121 | 2,853 |
| hpv_direct_colpo_3_17 | 285,757 | 8,109 | 1,725 | 164 | 38 | 366,852 | 163 | 116 | 2,625 |
| hpv_direct_colpo_3_18 | 282,806 | 8,018 | 1,665 | 166 | 37 | 362,988 | 161 | 117 | 2,613 |
| hpv_direct_colpo_3_19 | 278,500 | 7,762 | 1,623 | 172 | 39 | 356,118 | 155 | 115 | 2,567 |
| hpv_direct_colpo_3_2 | 294,204 | 27,712 | 2,972 | 116 | 37 | 571,320 | 211 | 117 | 3,104 |
| hpv_direct_colpo_3_20 | 280,011 | 6,898 | 1,306 | 197 | 49 | 348,990 | 130 | 105 | 2,147 |
| hpv_direct_colpo_3_21 | 275,709 | 6,652 | 1,273 | 201 | 50 | 342,227 | 126 | 104 | 2,123 |
| hpv_direct_colpo_3_3 | 292,602 | 27,326 | 2,820 | 119 | 37 | 565,861 | 208 | 117 | 3,013 |
| hpv_direct_colpo_3_4 | 291,887 | 26,076 | 2,676 | 127 | 35 | 552,644 | 200 | 119 | 3,011 |
| hpv_direct_colpo_3_5 | 289,791 | 25,762 | 2,558 | 129 | 34 | 547,407 | 198 | 120 | 2,967 |
| hpv_direct_colpo_3_6 | 285,695 | 24,979 | 2,329 | 153 | 39 | 535,484 | 174 | 115 | 2,707 |
| hpv_direct_colpo_3_7 | 293,221 | 16,628 | 2,641 | 118 | 34 | 459,497 | 209 | 120 | 3,147 |
| hpv_direct_colpo_3_8 | 291,626 | 16,282 | 2,529 | 116 | 31 | 454,447 | 211 | 123 | 3,153 |
| hpv_direct_colpo_3_9 | 289,530 | 15,968 | 2,411 | 117 | 30 | 449,206 | 209 | 124 | 3,112 |
| hpv_direct_colpo_4_1 | 389,715 | 30,300 | 3,374 | 92 | 25 | 692,715 | 235 | 129 | 3,356 |
| hpv_direct_colpo_4_10 | 376,357 | 17,560 | 2,597 | 109 | 22 | 551,959 | 218 | 132 | 3,224 |
| hpv_direct_colpo_4_11 | 388,217 | 13,681 | 2,558 | 119 | 29 | 525,027 | 208 | 125 | 3,104 |
| hpv_direct_colpo_4_12 | 387,053 | 13,340 | 2,552 | 118 | 28 | 520,449 | 209 | 126 | 3,115 |
| hpv_direct_colpo_4_13 | 384,721 | 12,881 | 2,464 | 117 | 25 | 513,529 | 210 | 129 | 3,133 |
| hpv_direct_colpo_4_14 | 384,262 | 12,592 | 2,487 | 118 | 25 | 510,180 | 209 | 129 | 3,128 |
| hpv_direct_colpo_4_15 | 379,063 | 11,968 | 2,329 | 121 | 23 | 498,738 | 206 | 131 | 3,098 |
| hpv_direct_colpo_4_16 | 374,758 | 11,718 | 2,296 | 125 | 24 | 491,940 | 202 | 130 | 3,079 |
| hpv_direct_colpo_4_2 | 387,624 | 30,013 | 3,285 | 90 | 21 | 687,749 | 237 | 133 | 3,377 |
| hpv_direct_colpo_4_3 | 384,668 | 29,907 | 3,210 | 95 | 22 | 683,739 | 232 | 132 | 3,332 |
| hpv_direct_colpo_4_4 | 383,249 | 29,272 | 3,093 | 102 | 23 | 675,966 | 225 | 131 | 3,256 |
| hpv_direct_colpo_4_5 | 376,607 | 27,355 | 2,744 | 121 | 27 | 650,159 | 206 | 127 | 3,077 |
| hpv_direct_colpo_4_6 | 389,428 | 20,292 | 3,024 | 95 | 25 | 592,350 | 232 | 129 | 3,346 |
| hpv_direct_colpo_4_7 | 385,218 | 18,282 | 2,822 | 101 | 21 | 568,038 | 226 | 132 | 3,308 |
| hpv_direct_colpo_4_8 | 382,265 | 18,188 | 2,763 | 103 | 20 | 564,140 | 224 | 134 | 3,297 |
| hpv_direct_colpo_4_9 | 377,956 | 17,929 | 2,720 | 110 | 23 | 557,249 | 217 | 131 | 3,249 |
| hpv_direct_colpo_5_1 | 489,077 | 39,864 | 3,874 | 73 | 22 | 887,712 | 254 | 132 | 3,486 |
| hpv_direct_colpo_5_2 | 484,867 | 37,860 | 3,679 | 79 | 18 | 863,464 | 248 | 136 | 3,459 |
| hpv_direct_colpo_5_3 | 486,060 | 34,900 | 3,768 | 75 | 19 | 835,056 | 252 | 135 | 3,493 |
| hpv_direct_colpo_5_4 | 478,774 | 31,823 | 3,474 | 81 | 16 | 796,999 | 246 | 138 | 3,451 |
| hpv_direct_colpo_6_1 | 587,308 | 43,101 | 4,194 | 61 | 19 | 1,018,320 | 266 | 135 | 3,567 |
| hpv_direct_colpo_6_10 | 569,624 | 39,122 | 3,732 | 74 | 13 | 960,839 | 253 | 141 | 3,499 |
| hpv_direct_colpo_6_11 | 580,831 | 38,232 | 4,033 | 63 | 14 | 963,155 | 264 | 140 | 3,585 |
| hpv_direct_colpo_6_12 | 576,210 | 36,470 | 3,875 | 66 | 12 | 940,907 | 261 | 142 | 3,561 |
| hpv_direct_colpo_6_2 | 493,603 | 40,478 | 3,866 | 71 | 23 | 898,383 | 256 | 131 | 3,468 |
| hpv_direct_colpo_6_3 | 404,834 | 36,610 | 3,542 | 87 | 26 | 770,937 | 240 | 128 | 3,355 |
| hpv_direct_colpo_6_4 | 583,810 | 42,298 | 4,099 | 59 | 15 | 1,006,794 | 268 | 139 | 3,590 |
| hpv_direct_colpo_6_5 | 494,783 | 38,428 | 3,775 | 74 | 17 | 879,066 | 252 | 136 | 3,486 |
| hpv_direct_colpo_6_6 | 578,152 | 41,397 | 3,975 | 62 | 12 | 992,124 | 265 | 141 | 3,579 |
| hpv_direct_colpo_6_7 | 583,352 | 42,013 | 4,129 | 60 | 15 | 1,003,485 | 267 | 139 | 3,599 |
| hpv_direct_colpo_6_8 | 583,300 | 41,263 | 4,125 | 62 | 16 | 995,926 | 265 | 138 | 3,585 |
| hpv_direct_colpo_6_9 | 579,739 | 40,473 | 4,001 | 63 | 13 | 984,469 | 264 | 141 | 3,581 |
| hpv_direct_colpo_7_1 | 683,032 | 45,202 | 4,417 | 56 | 18 | 1,135,055 | 270 | 136 | 3,598 |
| hpv_direct_colpo_7_10 | 675,911 | 44,297 | 4,288 | 50 | 10 | 1,118,886 | 277 | 144 | 3,656 |
| hpv_direct_colpo_7_11 | 668,582 | 43,566 | 4,153 | 55 | 10 | 1,104,247 | 272 | 144 | 3,632 |
| hpv_direct_colpo_7_12 | 579,542 | 39,695 | 3,830 | 70 | 12 | 976,496 | 257 | 141 | 3,529 |
| hpv_direct_colpo_7_13 | 675,827 | 44,012 | 4,297 | 51 | 10 | 1,115,948 | 276 | 144 | 3,652 |
| hpv_direct_colpo_7_14 | 670,935 | 43,467 | 4,196 | 53 | 9 | 1,105,604 | 274 | 145 | 3,648 |
| hpv_direct_colpo_7_15 | 668,409 | 39,674 | 4,095 | 57 | 9 | 1,065,148 | 270 | 145 | 3,626 |
| hpv_direct_colpo_7_2 | 496,809 | 40,223 | 3,965 | 70 | 21 | 899,037 | 257 | 133 | 3,507 |
| hpv_direct_colpo_7_3 | 680,942 | 44,985 | 4,389 | 52 | 13 | 1,130,795 | 275 | 141 | 3,648 |
| hpv_direct_colpo_7_4 | 498,451 | 38,595 | 3,823 | 73 | 17 | 884,399 | 254 | 137 | 3,500 |
| hpv_direct_colpo_7_5 | 680,827 | 44,956 | 4,391 | 51 | 13 | 1,130,390 | 276 | 141 | 3,650 |
| hpv_direct_colpo_7_6 | 679,533 | 44,790 | 4,361 | 50 | 11 | 1,127,428 | 277 | 143 | 3,663 |
| hpv_direct_colpo_7_7 | 676,394 | 44,624 | 4,293 | 50 | 10 | 1,122,632 | 277 | 144 | 3,659 |
| hpv_direct_colpo_7_8 | 582,887 | 41,620 | 4,031 | 60 | 12 | 999,091 | 267 | 142 | 3,592 |
| hpv_direct_colpo_7_9 | 678,665 | 44,518 | 4,349 | 51 | 11 | 1,123,844 | 276 | 143 | 3,656 |
| hpv_direct_colpo_8_1 | 775,073 | 46,760 | 4,534 | 47 | 11 | 1,242,677 | 280 | 143 | 3,673 |
| hpv_direct_colpo_8_10 | 583,212 | 39,863 | 3,875 | 68 | 12 | 981,843 | 259 | 142 | 3,540 |
| hpv_direct_colpo_8_11 | 494,188 | 38,363 | 3,711 | 76 | 16 | 877,821 | 251 | 138 | 3,473 |
| hpv_direct_colpo_8_12 | 491,305 | 38,074 | 3,663 | 79 | 17 | 872,050 | 248 | 137 | 3,445 |
| hpv_direct_colpo_8_13 | 765,381 | 45,863 | 4,401 | 46 | 8 | 1,224,016 | 281 | 146 | 3,688 |
| hpv_direct_colpo_8_2 | 683,112 | 45,071 | 4,409 | 51 | 12 | 1,133,818 | 276 | 142 | 3,653 |
| hpv_direct_colpo_8_3 | 589,546 | 42,547 | 4,168 | 57 | 14 | 1,015,014 | 270 | 140 | 3,606 |
| hpv_direct_colpo_8_4 | 500,764 | 38,684 | 3,844 | 72 | 17 | 887,603 | 255 | 137 | 3,504 |
| hpv_direct_colpo_8_5 | 772,122 | 46,729 | 4,515 | 46 | 9 | 1,239,412 | 281 | 145 | 3,687 |
| hpv_direct_colpo_8_6 | 585,887 | 41,751 | 4,064 | 59 | 12 | 1,003,394 | 267 | 142 | 3,600 |
| **hpv_direct_colpo_8_7** | **772,046** | **46,692** | **4,509** | **45** | **9** | **1,238,963** | **282** | **145** | **3,695** |
| hpv_direct_colpo_8_8 | 770,214 | 46,566 | 4,486 | 45 | 8 | 1,235,870 | 282 | 146 | 3,696 |
| hpv_direct_colpo_8_9 | 765,721 | 46,254 | 4,441 | 47 | 8 | 1,228,263 | 280 | 146 | 3,692 |
| **hpv_direct_colpo_9_1** | **864,162** | **48,247** | **4,598** | **42** | **7** | **1,346,636** | **285** | **147** | **3,711** |
| hpv_direct_colpo_9_2 | 773,936 | 46,792 | 4,527 | 45 | 8 | 1,241,861 | 282 | 145 | 3,693 |
| **hpv_direct_colpo_9_3** | **681,117** | **44,817** | **4,339** | **49** | **9** | **1,129,289** | **278** | **144** | **3,669** |
| hpv_direct_colpo_9_4 | 587,797 | 41,821 | 4,077 | 59 | 12 | 1,006,010 | 268 | 142 | 3,602 |
| hpv_direct_colpo_9_6 | 859,857 | 48,029 | 4,587 | 42 | 7 | 1,340,149 | 285 | 147 | 3,712 |
| hpv_direct_colpo_9_7 | 767,892 | 46,330 | 4,462 | 46 | 8 | 1,231,194 | 281 | 146 | 3,694 |
| hpv_direct_colpo_9_8 | 674,318 | 43,816 | 4,220 | 52 | 9 | 1,112,481 | 275 | 145 | 3,647 |
| hpv_direct_colpo_9_9 | 585,528 | 39,957 | 3,897 | 67 | 12 | 985,098 | 259 | 142 | 3,542 |
| no_screen | 0 | 0 | 0 | 327 | 154 | 0 | 0 | 0 | 0 |

Table S4c. Full baseline results in the unvaccinated cohort. All numbers per 100,000 individuals simulated. Strategies on the efficiency frontier are highlighted in bold font. Harms = screens + colposcopy referrals * 10

| **Strategy** | **Total screens** | **Colposcopy referrals** | **CIN2/3 treatments** | **Cancers** | **Cancer deaths** | **Harms** | **Cancers prevented** | **Cancer deaths prevented** | **Life years gained** |
| --- | --- | --- | --- | --- | --- | --- | --- | --- | --- |
| current_cyt | 1,626,545 | 29,928 | 8,239 | 198 | 30 | 1,925,825 | 702 | 383 | 10,207 |
| hpv_16/18/9v_1_1 | 105,949 | 9,605 | 2,338 | 548 | 237 | 201,999 | 352 | 177 | 5,375 |
| hpv_16/18/9v_1_2 | 102,334 | 5,198 | 2,171 | 514 | 184 | 154,315 | 386 | 229 | 6,349 |
| hpv_16/18/9v_1_3 | 100,779 | 3,865 | 1,780 | 565 | 176 | 139,432 | 335 | 237 | 5,975 |
| hpv_16/18/9v_1_4 | 99,219 | 3,036 | 1,436 | 637 | 193 | 129,578 | 263 | 220 | 4,966 |
| hpv_16/18/9v_1_5 | 97,259 | 2,188 | 1,045 | 707 | 227 | 119,141 | 193 | 186 | 3,685 |
| hpv_16/18/9v_2_1 | 205,243 | 12,233 | 3,484 | 385 | 110 | 327,570 | 515 | 304 | 8,173 |
| hpv_16/18/9v_2_2 | 203,366 | 11,530 | 3,209 | 420 | 120 | 318,665 | 480 | 294 | 7,644 |
| hpv_16/18/9v_2_3 | 201,491 | 7,551 | 3,098 | 409 | 109 | 277,004 | 491 | 304 | 7,956 |
| hpv_16/18/9v_2_4 | 199,664 | 6,950 | 2,921 | 423 | 107 | 269,162 | 477 | 306 | 7,810 |
| hpv_16/18/9v_2_5 | 197,866 | 6,468 | 2,684 | 437 | 111 | 262,550 | 463 | 302 | 7,531 |
| hpv_16/18/9v_2_6 | 197,960 | 5,450 | 2,398 | 504 | 127 | 252,459 | 396 | 286 | 6,872 |
| hpv_16/18/9v_2_7 | 196,194 | 5,039 | 2,222 | 506 | 124 | 246,581 | 394 | 289 | 6,810 |
| hpv_16/18/9v_2_8 | 193,929 | 4,749 | 2,075 | 515 | 125 | 241,416 | 385 | 288 | 6,655 |
| hpv_16/18/9v_3_1 | 312,908 | 25,794 | 4,879 | 278 | 83 | 570,845 | 622 | 330 | 9,161 |
| hpv_16/18/9v_3_10 | 298,521 | 13,051 | 3,732 | 345 | 74 | 429,027 | 555 | 340 | 8,639 |
| hpv_16/18/9v_3_11 | 295,410 | 12,802 | 3,641 | 357 | 77 | 423,433 | 543 | 336 | 8,526 |
| hpv_16/18/9v_3_12 | 296,984 | 8,605 | 3,443 | 370 | 79 | 383,034 | 530 | 334 | 8,406 |
| hpv_16/18/9v_3_13 | 294,747 | 8,361 | 3,342 | 370 | 74 | 378,352 | 530 | 339 | 8,412 |
| hpv_16/18/9v_3_14 | 291,635 | 8,114 | 3,253 | 382 | 78 | 372,776 | 518 | 336 | 8,299 |
| hpv_16/18/9v_3_15 | 289,777 | 7,478 | 3,053 | 400 | 82 | 364,556 | 500 | 331 | 8,082 |
| hpv_16/18/9v_3_16 | 285,315 | 7,335 | 3,005 | 411 | 87 | 358,668 | 489 | 326 | 7,992 |
| hpv_16/18/9v_3_17 | 291,084 | 6,202 | 2,597 | 474 | 103 | 353,109 | 426 | 310 | 7,179 |
| hpv_16/18/9v_3_18 | 287,993 | 5,979 | 2,528 | 481 | 103 | 347,781 | 419 | 310 | 7,140 |
| hpv_16/18/9v_3_19 | 283,539 | 5,838 | 2,482 | 492 | 108 | 341,916 | 408 | 305 | 7,047 |
| hpv_16/18/9v_3_2 | 311,082 | 25,195 | 4,706 | 291 | 80 | 563,032 | 609 | 333 | 9,045 |
| hpv_16/18/9v_3_20 | 284,418 | 4,587 | 1,895 | 577 | 143 | 330,287 | 323 | 270 | 5,654 |
| hpv_16/18/9v_3_21 | 279,979 | 4,459 | 1,860 | 585 | 145 | 324,568 | 315 | 268 | 5,608 |
| hpv_16/18/9v_3_3 | 309,276 | 24,717 | 4,470 | 304 | 83 | 556,446 | 596 | 330 | 8,777 |
| hpv_16/18/9v_3_4 | 307,728 | 23,490 | 4,211 | 336 | 80 | 542,628 | 564 | 333 | 8,677 |
| hpv_16/18/9v_3_5 | 305,463 | 23,204 | 4,065 | 344 | 82 | 537,499 | 556 | 332 | 8,534 |
| hpv_16/18/9v_3_6 | 300,763 | 22,174 | 3,683 | 414 | 101 | 522,498 | 486 | 312 | 7,724 |
| hpv_16/18/9v_3_7 | 304,055 | 14,398 | 4,208 | 307 | 74 | 448,036 | 593 | 339 | 9,048 |
| hpv_16/18/9v_3_8 | 302,286 | 13,981 | 4,036 | 307 | 69 | 442,093 | 593 | 344 | 9,017 |
| hpv_16/18/9v_3_9 | 300,023 | 13,695 | 3,893 | 314 | 70 | 436,972 | 586 | 343 | 8,884 |
| hpv_16/18/9v_4_1 | 408,523 | 26,846 | 5,226 | 239 | 53 | 676,979 | 661 | 360 | 9,612 |
| hpv_16/18/9v_4_10 | 388,019 | 14,344 | 4,104 | 298 | 56 | 531,463 | 602 | 357 | 9,132 |
| hpv_16/18/9v_4_11 | 397,363 | 10,913 | 3,900 | 337 | 71 | 506,496 | 563 | 342 | 8,678 |
| hpv_16/18/9v_4_12 | 395,990 | 10,603 | 3,873 | 337 | 69 | 502,021 | 563 | 344 | 8,687 |
| hpv_16/18/9v_4_13 | 393,374 | 10,199 | 3,759 | 337 | 64 | 495,367 | 563 | 350 | 8,707 |
| hpv_16/18/9v_4_14 | 392,733 | 9,849 | 3,767 | 342 | 65 | 491,225 | 558 | 348 | 8,657 |
| hpv_16/18/9v_4_15 | 387,110 | 9,086 | 3,545 | 353 | 64 | 477,973 | 547 | 349 | 8,561 |
| hpv_16/18/9v_4_16 | 382,663 | 8,962 | 3,511 | 361 | 66 | 472,282 | 539 | 347 | 8,515 |
| hpv_16/18/9v_4_2 | 406,279 | 26,604 | 5,125 | 239 | 48 | 672,315 | 661 | 366 | 9,621 |
| hpv_16/18/9v_4_3 | 403,168 | 26,359 | 5,034 | 251 | 51 | 666,755 | 649 | 362 | 9,516 |
| hpv_16/18/9v_4_4 | 401,310 | 25,723 | 4,839 | 268 | 55 | 658,545 | 632 | 358 | 9,312 |
| hpv_16/18/9v_4_5 | 393,426 | 23,844 | 4,278 | 327 | 67 | 631,863 | 573 | 346 | 8,792 |
| hpv_16/18/9v_4_6 | 402,878 | 17,008 | 4,717 | 253 | 54 | 572,958 | 647 | 359 | 9,488 |
| hpv_16/18/9v_4_7 | 397,366 | 15,143 | 4,406 | 276 | 50 | 548,792 | 624 | 363 | 9,344 |
| hpv_16/18/9v_4_8 | 394,269 | 14,917 | 4,338 | 284 | 50 | 543,440 | 616 | 363 | 9,306 |
| hpv_16/18/9v_4_9 | 389,801 | 14,782 | 4,291 | 295 | 56 | 537,621 | 605 | 358 | 9,214 |
| hpv_16/18/9v_5_1 | 514,212 | 34,762 | 5,965 | 180 | 43 | 861,834 | 720 | 370 | 9,999 |
| hpv_16/18/9v_5_2 | 508,700 | 32,906 | 5,667 | 202 | 39 | 837,757 | 698 | 374 | 9,896 |
| hpv_16/18/9v_5_3 | 508,078 | 30,247 | 5,777 | 194 | 39 | 810,545 | 706 | 374 | 9,965 |
| hpv_16/18/9v_5_4 | 498,770 | 27,330 | 5,327 | 222 | 38 | 772,069 | 678 | 376 | 9,764 |
| hpv_16/18/9v_6_1 | 614,756 | 37,053 | 6,403 | 150 | 37 | 985,289 | 750 | 376 | 10,212 |
| hpv_16/18/9v_6_10 | 594,502 | 33,238 | 5,716 | 196 | 32 | 926,885 | 704 | 381 | 9,957 |
| hpv_16/18/9v_6_11 | 605,285 | 32,534 | 6,121 | 166 | 29 | 930,622 | 734 | 384 | 10,171 |
| hpv_16/18/9v_6_12 | 599,484 | 30,773 | 5,886 | 181 | 28 | 907,213 | 719 | 385 | 10,082 |
| hpv_16/18/9v_6_2 | 520,120 | 35,323 | 5,964 | 176 | 46 | 873,355 | 724 | 367 | 9,975 |
| hpv_16/18/9v_6_3 | 432,480 | 32,462 | 5,501 | 215 | 53 | 757,101 | 685 | 360 | 9,705 |
| hpv_16/18/9v_6_4 | 610,766 | 36,337 | 6,264 | 150 | 29 | 974,139 | 750 | 384 | 10,237 |
| hpv_16/18/9v_6_5 | 522,655 | 33,468 | 5,801 | 190 | 37 | 857,332 | 710 | 377 | 9,978 |
| hpv_16/18/9v_6_6 | 604,514 | 35,245 | 6,067 | 163 | 28 | 956,963 | 737 | 385 | 10,164 |
| hpv_16/18/9v_6_7 | 610,139 | 36,000 | 6,284 | 153 | 30 | 970,140 | 747 | 383 | 10,232 |
| hpv_16/18/9v_6_8 | 609,564 | 35,331 | 6,262 | 160 | 32 | 962,872 | 740 | 382 | 10,199 |
| hpv_16/18/9v_6_9 | 605,561 | 34,588 | 6,085 | 167 | 28 | 951,440 | 733 | 385 | 10,166 |
| hpv_16/18/9v_7_1 | 712,073 | 38,236 | 6,670 | 141 | 34 | 1,094,433 | 759 | 379 | 10,278 |
| hpv_16/18/9v_7_10 | 704,413 | 37,250 | 6,481 | 135 | 21 | 1,076,916 | 765 | 392 | 10,365 |
| hpv_16/18/9v_7_11 | 696,611 | 36,667 | 6,312 | 145 | 23 | 1,063,279 | 755 | 391 | 10,294 |
| hpv_16/18/9v_7_12 | 608,462 | 33,804 | 5,850 | 184 | 30 | 946,506 | 716 | 384 | 10,036 |
| hpv_16/18/9v_7_13 | 704,168 | 36,986 | 6,480 | 137 | 22 | 1,074,031 | 763 | 391 | 10,351 |
| hpv_16/18/9v_7_14 | 698,881 | 36,409 | 6,345 | 145 | 22 | 1,062,973 | 755 | 392 | 10,309 |
| hpv_16/18/9v_7_15 | 694,003 | 32,932 | 6,175 | 159 | 23 | 1,023,322 | 741 | 391 | 10,231 |
| hpv_16/18/9v_7_2 | 524,668 | 35,057 | 6,067 | 174 | 42 | 875,241 | 726 | 371 | 10,052 |
| hpv_16/18/9v_7_3 | 709,889 | 38,071 | 6,639 | 133 | 25 | 1,090,602 | 767 | 388 | 10,365 |
| hpv_16/18/9v_7_4 | 527,639 | 33,607 | 5,853 | 187 | 36 | 863,712 | 713 | 377 | 10,003 |
| hpv_16/18/9v_7_5 | 709,750 | 37,975 | 6,629 | 133 | 26 | 1,089,501 | 767 | 388 | 10,362 |
| hpv_16/18/9v_7_6 | 708,392 | 37,821 | 6,592 | 131 | 23 | 1,086,606 | 769 | 390 | 10,382 |
| hpv_16/18/9v_7_7 | 705,090 | 37,533 | 6,504 | 134 | 21 | 1,080,420 | 766 | 392 | 10,372 |
| hpv_16/18/9v_7_8 | 611,064 | 35,452 | 6,136 | 159 | 27 | 965,581 | 741 | 386 | 10,197 |
| hpv_16/18/9v_7_9 | 707,381 | 37,587 | 6,565 | 133 | 23 | 1,083,247 | 767 | 390 | 10,373 |
| hpv_16/18/9v_8_1 | 805,462 | 38,893 | 6,794 | 124 | 22 | 1,194,395 | 776 | 391 | 10,414 |
| hpv_16/18/9v_8_10 | 613,460 | 33,938 | 5,903 | 181 | 29 | 952,836 | 719 | 384 | 10,061 |
| hpv_16/18/9v_8_11 | 523,110 | 33,178 | 5,710 | 197 | 36 | 854,889 | 703 | 377 | 9,930 |
| hpv_16/18/9v_8_12 | 520,116 | 32,977 | 5,651 | 203 | 39 | 849,883 | 697 | 375 | 9,874 |
| hpv_16/18/9v_8_13 | 795,225 | 37,939 | 6,612 | 127 | 17 | 1,174,619 | 773 | 396 | 10,422 |
| hpv_16/18/9v_8_2 | 712,503 | 38,119 | 6,659 | 132 | 24 | 1,093,695 | 768 | 389 | 10,380 |
| hpv_16/18/9v_8_3 | 618,171 | 36,522 | 6,334 | 147 | 28 | 983,395 | 753 | 385 | 10,266 |
| hpv_16/18/9v_8_4 | 530,494 | 33,657 | 5,874 | 186 | 35 | 867,061 | 714 | 378 | 10,010 |
| hpv_16/18/9v_8_5 | 802,404 | 38,718 | 6,768 | 125 | 19 | 1,189,585 | 775 | 394 | 10,432 |
| hpv_16/18/9v_8_6 | 614,968 | 35,544 | 6,171 | 157 | 26 | 970,403 | 743 | 387 | 10,209 |
| **hpv_16/18/9v_8_7** | **802,315** | **38,700** | **6,760** | **122** | **18** | **1,189,315** | **778** | **395** | **10,444** |
| hpv_16/18/9v_8_8 | 800,410 | 38,572 | 6,733 | 124 | 18 | 1,186,130 | 776 | 395 | 10,440 |
| hpv_16/18/9v_8_9 | 795,750 | 38,399 | 6,688 | 128 | 18 | 1,179,740 | 772 | 395 | 10,428 |
| **hpv_16/18/9v_9_1** | **895,766** | **39,327** | **6,854** | **117** | **16** | **1,289,036** | **783** | **397** | **10,474** |
| hpv_16/18/9v_9_2 | 804,504 | 38,747 | 6,778 | 123 | 18 | 1,191,972 | 777 | 395 | 10,435 |
| hpv_16/18/9v_9_3 | 710,921 | 37,646 | 6,546 | 132 | 21 | 1,087,385 | 768 | 392 | 10,387 |
| hpv_16/18/9v_9_4 | 617,214 | 35,568 | 6,179 | 156 | 26 | 972,895 | 744 | 387 | 10,218 |
| hpv_16/18/9v_9_6 | 891,328 | 39,221 | 6,843 | 118 | 16 | 1,283,535 | 781 | 398 | 10,470 |
| hpv_16/18/9v_9_7 | 798,362 | 38,450 | 6,708 | 126 | 18 | 1,182,863 | 774 | 396 | 10,431 |
| hpv_16/18/9v_9_8 | 704,020 | 36,850 | 6,380 | 141 | 21 | 1,072,521 | 759 | 392 | 10,323 |
| hpv_16/18/9v_9_9 | 616,314 | 33,988 | 5,921 | 179 | 28 | 956,189 | 721 | 385 | 10,075 |
| hpv_16/18_1_1 | 110,467 | 6,360 | 2,255 | 550 | 236 | 174,070 | 350 | 177 | 5,388 |
| **hpv_16/18_1_2** | **104,422** | **3,793** | **2,074** | **520** | **186** | **142,356** | **380** | **227** | **6,294** |
| hpv_16/18_1_3 | 102,179 | 2,961 | 1,696 | 572 | 178 | 131,786 | 328 | 235 | 5,921 |
| hpv_16/18_1_4 | 100,217 | 2,397 | 1,362 | 643 | 195 | 124,188 | 257 | 218 | 4,903 |
| hpv_16/18_1_5 | 97,898 | 1,784 | 989 | 711 | 229 | 115,734 | 189 | 184 | 3,635 |
| **hpv_16/18_2_1** | **210,708** | **8,358** | **3,336** | **391** | **111** | **294,291** | **509** | **302** | **8,128** |
| hpv_16/18_2_2 | 208,497 | 7,887 | 3,075 | 425 | 121 | 287,369 | 475 | 292 | 7,611 |
| **hpv_16/18_2_3** | **204,489** | **5,529** | **2,950** | **418** | **111** | **259,774** | **482** | **302** | **7,895** |
| hpv_16/18_2_4 | 202,340 | 5,151 | 2,779 | 431 | 109 | 253,847 | 469 | 304 | 7,750 |
| hpv_16/18_2_5 | 200,319 | 4,811 | 2,558 | 444 | 113 | 248,434 | 456 | 300 | 7,471 |
| hpv_16/18_2_6 | 199,919 | 4,158 | 2,276 | 512 | 129 | 241,502 | 388 | 284 | 6,819 |
| hpv_16/18_2_7 | 197,948 | 3,881 | 2,113 | 514 | 126 | 236,758 | 386 | 287 | 6,743 |
| hpv_16/18_2_8 | 195,560 | 3,669 | 1,977 | 522 | 127 | 232,250 | 378 | 286 | 6,593 |
| hpv_16/18_3_1 | 325,076 | 17,478 | 4,665 | 285 | 84 | 499,860 | 615 | 330 | 9,135 |
| hpv_16/18_3_10 | 304,214 | 9,000 | 3,570 | 352 | 75 | 394,216 | 548 | 338 | 8,590 |
| hpv_16/18_3_11 | 300,996 | 8,836 | 3,487 | 364 | 79 | 389,352 | 536 | 335 | 8,486 |
| hpv_16/18_3_12 | 300,311 | 6,330 | 3,275 | 379 | 81 | 363,614 | 521 | 332 | 8,341 |
| hpv_16/18_3_13 | 297,959 | 6,166 | 3,181 | 379 | 76 | 359,617 | 521 | 337 | 8,342 |
| hpv_16/18_3_14 | 294,740 | 5,998 | 3,097 | 391 | 80 | 354,723 | 509 | 334 | 8,234 |
| hpv_16/18_3_15 | 292,558 | 5,587 | 2,904 | 409 | 84 | 348,432 | 491 | 329 | 8,013 |
| hpv_16/18_3_16 | 288,045 | 5,480 | 2,860 | 419 | 89 | 342,848 | 481 | 324 | 7,929 |
| hpv_16/18_3_17 | 293,255 | 4,738 | 2,462 | 482 | 105 | 340,639 | 418 | 308 | 7,110 |
| hpv_16/18_3_18 | 290,060 | 4,593 | 2,400 | 489 | 105 | 335,992 | 411 | 308 | 7,071 |
| hpv_16/18_3_19 | 285,555 | 4,490 | 2,358 | 501 | 110 | 330,454 | 399 | 303 | 6,983 |
| hpv_16/18_3_2 | 322,928 | 17,108 | 4,499 | 297 | 81 | 494,003 | 602 | 332 | 9,004 |
| hpv_16/18_3_20 | 285,863 | 3,614 | 1,797 | 584 | 145 | 322,006 | 316 | 268 | 5,596 |
| hpv_16/18_3_21 | 281,377 | 3,522 | 1,765 | 591 | 147 | 316,597 | 309 | 266 | 5,555 |
| hpv_16/18_3_3 | 320,909 | 16,770 | 4,281 | 310 | 84 | 488,605 | 590 | 329 | 8,751 |
| hpv_16/18_3_4 | 318,704 | 16,044 | 4,033 | 342 | 81 | 479,141 | 558 | 332 | 8,652 |
| hpv_16/18_3_5 | 316,306 | 15,832 | 3,898 | 350 | 82 | 474,629 | 550 | 331 | 8,512 |
| hpv_16/18_3_6 | 311,115 | 15,153 | 3,535 | 418 | 102 | 462,645 | 482 | 311 | 7,709 |
| hpv_16/18_3_7 | 310,437 | 9,864 | 4,018 | 315 | 76 | 409,072 | 585 | 337 | 8,995 |
| **hpv_16/18_3_8** | **308,467** | **9,587** | **3,857** | **316** | **71** | **404,339** | **584** | **342** | **8,969** |
| hpv_16/18_3_9 | 306,076 | 9,380 | 3,725 | 323 | 72 | 399,879 | 577 | 342 | 8,842 |
| hpv_16/18_4_1 | 421,028 | 18,282 | 4,994 | 246 | 54 | 603,848 | 654 | 359 | 9,577 |
| hpv_16/18_4_10 | 394,257 | 9,898 | 3,925 | 307 | 57 | 493,237 | 593 | 356 | 9,085 |
| hpv_16/18_4_11 | 401,790 | 7,800 | 3,723 | 346 | 72 | 479,785 | 554 | 341 | 8,625 |
| hpv_16/18_4_12 | 400,240 | 7,626 | 3,692 | 347 | 70 | 476,496 | 553 | 343 | 8,638 |
| hpv_16/18_4_13 | 397,418 | 7,375 | 3,583 | 346 | 65 | 471,165 | 554 | 348 | 8,648 |
| hpv_16/18_4_14 | 396,557 | 7,208 | 3,582 | 351 | 67 | 468,636 | 549 | 346 | 8,593 |
| hpv_16/18_4_15 | 390,542 | 6,725 | 3,370 | 363 | 66 | 457,795 | 537 | 347 | 8,495 |
| hpv_16/18_4_16 | 386,049 | 6,635 | 3,340 | 370 | 68 | 452,398 | 530 | 345 | 8,461 |
| hpv_16/18_4_2 | 418,677 | 18,117 | 4,899 | 247 | 49 | 599,851 | 653 | 364 | 9,576 |
| hpv_16/18_4_3 | 415,455 | 17,950 | 4,815 | 258 | 52 | 594,956 | 642 | 361 | 9,478 |
| hpv_16/18_4_4 | 413,266 | 17,540 | 4,624 | 275 | 56 | 588,668 | 625 | 357 | 9,275 |
| hpv_16/18_4_5 | 404,462 | 16,350 | 4,100 | 333 | 68 | 567,961 | 567 | 345 | 8,766 |
| **hpv_16/18_4_6** | **410,486** | **11,515** | **4,508** | **261** | **56** | **525,637** | **638** | **358** | **9,431** |
| hpv_16/18_4_7 | 403,960 | 10,439 | 4,204 | 285 | 52 | 508,348 | 615 | 361 | 9,291 |
| hpv_16/18_4_8 | 400,765 | 10,297 | 4,142 | 292 | 52 | 503,737 | 608 | 361 | 9,263 |
| hpv_16/18_4_9 | 396,247 | 10,192 | 4,098 | 303 | 57 | 498,166 | 597 | 356 | 9,166 |
| hpv_16/18_5_1 | 530,850 | 22,965 | 5,715 | 189 | 44 | 760,504 | 711 | 369 | 9,968 |
| hpv_16/18_5_2 | 524,339 | 21,904 | 5,426 | 210 | 40 | 743,381 | 690 | 373 | 9,865 |
| hpv_16/18_5_3 | 522,268 | 20,374 | 5,525 | 202 | 40 | 726,013 | 698 | 373 | 9,934 |
| hpv_16/18_5_4 | 511,379 | 18,678 | 5,089 | 230 | 39 | 698,160 | 670 | 374 | 9,729 |
| hpv_16/18_6_1 | 632,504 | 24,421 | 6,148 | 159 | 38 | 876,714 | 741 | 375 | 10,178 |
| hpv_16/18_6_10 | 610,195 | 22,187 | 5,476 | 204 | 33 | 832,067 | 696 | 380 | 9,918 |
| hpv_16/18_6_11 | 620,527 | 21,854 | 5,854 | 175 | 30 | 839,069 | 725 | 383 | 10,135 |
| hpv_16/18_6_12 | 613,799 | 20,799 | 5,629 | 189 | 29 | 821,784 | 711 | 384 | 10,052 |
| hpv_16/18_6_2 | 537,098 | 23,247 | 5,722 | 184 | 47 | 769,573 | 716 | 366 | 9,942 |
| hpv_16/18_6_3 | 447,937 | 21,502 | 5,253 | 224 | 55 | 662,959 | 676 | 358 | 9,660 |
| hpv_16/18_6_4 | 628,112 | 23,992 | 6,009 | 160 | 30 | 868,037 | 740 | 383 | 10,202 |
| hpv_16/18_6_5 | 538,473 | 22,242 | 5,538 | 199 | 38 | 760,894 | 701 | 375 | 9,943 |
| hpv_16/18_6_6 | 621,254 | 23,366 | 5,814 | 173 | 29 | 854,918 | 727 | 384 | 10,116 |
| hpv_16/18_6_7 | 627,277 | 23,841 | 6,021 | 162 | 31 | 865,691 | 738 | 382 | 10,192 |
| hpv_16/18_6_8 | 626,323 | 23,485 | 5,991 | 168 | 32 | 861,173 | 732 | 381 | 10,169 |
| hpv_16/18_6_9 | 621,919 | 23,030 | 5,823 | 176 | 29 | 852,216 | 724 | 384 | 10,130 |
| hpv_16/18_7_1 | 730,281 | 25,241 | 6,401 | 149 | 34 | 982,686 | 751 | 379 | 10,248 |
| hpv_16/18_7_10 | 722,096 | 24,659 | 6,217 | 144 | 22 | 968,682 | 756 | 391 | 10,327 |
| hpv_16/18_7_11 | 714,015 | 24,275 | 6,059 | 154 | 24 | 956,764 | 746 | 390 | 10,258 |
| hpv_16/18_7_12 | 624,335 | 22,526 | 5,587 | 193 | 31 | 849,598 | 707 | 382 | 9,996 |
| hpv_16/18_7_13 | 721,690 | 24,530 | 6,211 | 146 | 23 | 966,994 | 754 | 390 | 10,314 |
| hpv_16/18_7_14 | 716,098 | 24,167 | 6,079 | 153 | 23 | 957,764 | 747 | 391 | 10,273 |
| hpv_16/18_7_15 | 709,338 | 22,176 | 5,906 | 167 | 23 | 931,097 | 733 | 390 | 10,203 |
| **hpv_16/18_7_2** | **541,375** | **23,162** | **5,804** | **182** | **43** | **772,993** | **718** | **370** | **10,021** |
| hpv_16/18_7_3 | 728,002 | 25,142 | 6,373 | 142 | 26 | 979,417 | 758 | 387 | 10,333 |
| hpv_16/18_7_4 | 543,507 | 22,340 | 5,582 | 196 | 37 | 766,908 | 704 | 376 | 9,964 |
| hpv_16/18_7_5 | 727,812 | 25,087 | 6,362 | 141 | 27 | 978,687 | 759 | 387 | 10,325 |
| **hpv_16/18_7_6** | **726,372** | **25,004** | **6,327** | **140** | **24** | **976,416** | **759** | **389** | **10,343** |
| hpv_16/18_7_7 | 722,930 | 24,819 | 6,244 | 144 | 23 | 971,116 | 756 | 390 | 10,326 |
| hpv_16/18_7_8 | 627,843 | 23,497 | 5,870 | 167 | 28 | 862,811 | 733 | 385 | 10,159 |
| hpv_16/18_7_9 | 725,216 | 24,867 | 6,297 | 142 | 24 | 973,889 | 758 | 389 | 10,339 |
| hpv_16/18_8_1 | 823,866 | 25,738 | 6,521 | 133 | 23 | 1,081,243 | 767 | 390 | 10,378 |
| hpv_16/18_8_10 | 629,380 | 22,617 | 5,629 | 190 | 30 | 855,546 | 710 | 383 | 10,026 |
| hpv_16/18_8_11 | 538,793 | 22,051 | 5,446 | 206 | 37 | 759,301 | 694 | 376 | 9,886 |
| hpv_16/18_8_12 | 535,682 | 21,924 | 5,389 | 212 | 40 | 754,920 | 688 | 373 | 9,826 |
| hpv_16/18_8_13 | 813,138 | 25,164 | 6,341 | 136 | 18 | 1,064,774 | 764 | 395 | 10,388 |
| hpv_16/18_8_2 | 730,623 | 25,176 | 6,388 | 141 | 25 | 982,382 | 759 | 388 | 10,340 |
| **hpv_16/18_8_3** | **635,573** | **24,122** | **6,067** | **156** | **29** | **876,792** | **744** | **384** | **10,229** |
| hpv_16/18_8_4 | 546,381 | 22,375 | 5,599 | 195 | 36 | 770,134 | 705 | 377 | 9,975 |
| hpv_16/18_8_5 | 820,718 | 25,632 | 6,495 | 134 | 20 | 1,077,036 | 766 | 393 | 10,397 |
| hpv_16/18_8_6 | 631,788 | 23,558 | 5,900 | 166 | 28 | 867,372 | 734 | 386 | 10,172 |
| **hpv_16/18_8_7** | **820,633** | **25,616** | **6,487** | **131** | **19** | **1,076,796** | **769** | **394** | **10,408** |
| hpv_16/18_8_8 | 818,658 | 25,539 | 6,464 | 133 | 19 | 1,074,047 | 767 | 394 | 10,406 |
| hpv_16/18_8_9 | 813,921 | 25,423 | 6,420 | 136 | 19 | 1,068,146 | 764 | 394 | 10,386 |
| hpv_16/18_9_1 | 914,268 | 26,078 | 6,575 | 126 | 17 | 1,175,048 | 774 | 396 | 10,432 |
| hpv_16/18_9_2 | 822,817 | 25,651 | 6,502 | 133 | 19 | 1,079,329 | 767 | 394 | 10,403 |
| hpv_16/18_9_3 | 728,775 | 24,897 | 6,280 | 141 | 22 | 977,745 | 759 | 391 | 10,351 |
| hpv_16/18_9_4 | 634,039 | 23,589 | 5,908 | 165 | 27 | 869,930 | 735 | 386 | 10,176 |
| hpv_16/18_9_6 | 909,791 | 26,015 | 6,568 | 128 | 16 | 1,169,938 | 772 | 397 | 10,437 |
| hpv_16/18_9_7 | 816,543 | 25,455 | 6,438 | 135 | 19 | 1,071,094 | 765 | 395 | 10,397 |
| hpv_16/18_9_8 | 721,469 | 24,398 | 6,114 | 150 | 22 | 965,446 | 750 | 391 | 10,287 |
| hpv_16/18_9_9 | 632,246 | 22,650 | 5,645 | 189 | 30 | 858,743 | 711 | 384 | 10,025 |
| hpv_direct_colpo_1_1 | 99,312 | 14,558 | 2,433 | 548 | 238 | 244,889 | 352 | 175 | 5,356 |
| hpv_direct_colpo_1_2 | 98,844 | 7,700 | 2,288 | 508 | 184 | 175,849 | 392 | 230 | 6,380 |
| hpv_direct_colpo_1_3 | 98,071 | 5,765 | 1,883 | 558 | 174 | 155,720 | 342 | 239 | 6,043 |
| hpv_direct_colpo_1_4 | 96,870 | 4,673 | 1,539 | 631 | 191 | 143,604 | 269 | 222 | 5,036 |
| hpv_direct_colpo_1_5 | 95,361 | 3,516 | 1,142 | 702 | 225 | 130,523 | 198 | 189 | 3,746 |
| hpv_direct_colpo_2_1 | 196,298 | 18,834 | 3,678 | 378 | 108 | 384,635 | 522 | 305 | 8,219 |
| hpv_direct_colpo_2_2 | 194,852 | 17,816 | 3,398 | 415 | 118 | 373,010 | 485 | 295 | 7,685 |
| hpv_direct_colpo_2_3 | 195,743 | 11,689 | 3,296 | 401 | 108 | 312,634 | 499 | 305 | 8,012 |
| hpv_direct_colpo_2_4 | 194,328 | 10,779 | 3,125 | 415 | 105 | 302,122 | 485 | 308 | 7,875 |
| hpv_direct_colpo_2_5 | 192,711 | 10,205 | 2,870 | 428 | 109 | 294,758 | 472 | 304 | 7,595 |
| hpv_direct_colpo_2_6 | 193,438 | 8,666 | 2,577 | 496 | 126 | 280,103 | 404 | 288 | 6,941 |
| hpv_direct_colpo_2_7 | 191,840 | 8,166 | 2,391 | 497 | 121 | 273,499 | 403 | 292 | 6,878 |
| hpv_direct_colpo_2_8 | 189,740 | 7,792 | 2,222 | 505 | 123 | 267,663 | 395 | 290 | 6,728 |
| hpv_direct_colpo_3_1 | 295,482 | 38,229 | 5,167 | 272 | 82 | 677,773 | 628 | 331 | 9,192 |
| hpv_direct_colpo_3_10 | 288,103 | 20,789 | 3,966 | 336 | 72 | 495,991 | 564 | 341 | 8,692 |
| hpv_direct_colpo_3_11 | 285,146 | 20,470 | 3,862 | 348 | 75 | 489,841 | 552 | 338 | 8,584 |
| hpv_direct_colpo_3_12 | 289,615 | 13,961 | 3,701 | 360 | 78 | 429,228 | 540 | 335 | 8,459 |
| hpv_direct_colpo_3_13 | 287,525 | 13,641 | 3,582 | 359 | 72 | 423,939 | 541 | 341 | 8,475 |
| hpv_direct_colpo_3_14 | 284,570 | 13,317 | 3,479 | 371 | 75 | 417,740 | 529 | 338 | 8,367 |
| hpv_direct_colpo_3_15 | 283,127 | 12,371 | 3,282 | 389 | 80 | 406,840 | 511 | 333 | 8,148 |
| hpv_direct_colpo_3_16 | 278,830 | 12,133 | 3,223 | 401 | 85 | 400,161 | 499 | 328 | 8,054 |
| hpv_direct_colpo_3_17 | 285,108 | 10,557 | 2,812 | 464 | 101 | 390,677 | 436 | 312 | 7,243 |
| hpv_direct_colpo_3_18 | 282,163 | 10,257 | 2,733 | 471 | 101 | 384,735 | 429 | 313 | 7,208 |
| hpv_direct_colpo_3_19 | 277,870 | 10,019 | 2,676 | 483 | 106 | 378,064 | 417 | 307 | 7,113 |
| hpv_direct_colpo_3_2 | 294,067 | 37,319 | 4,998 | 285 | 79 | 667,259 | 615 | 334 | 9,083 |
| hpv_direct_colpo_3_20 | 279,153 | 8,507 | 2,078 | 569 | 140 | 364,228 | 331 | 273 | 5,729 |
| hpv_direct_colpo_3_21 | 274,871 | 8,281 | 2,035 | 576 | 143 | 357,680 | 324 | 271 | 5,684 |
| hpv_direct_colpo_3_3 | 292,450 | 36,751 | 4,745 | 298 | 82 | 659,964 | 602 | 331 | 8,821 |
| hpv_direct_colpo_3_4 | 291,663 | 34,906 | 4,471 | 331 | 79 | 640,720 | 569 | 334 | 8,709 |
| hpv_direct_colpo_3_5 | 289,560 | 34,533 | 4,304 | 338 | 80 | 634,892 | 562 | 333 | 8,570 |
| hpv_direct_colpo_3_6 | 285,371 | 33,158 | 3,906 | 409 | 100 | 616,947 | 491 | 313 | 7,753 |
| hpv_direct_colpo_3_7 | 292,975 | 22,568 | 4,472 | 299 | 73 | 518,651 | 601 | 340 | 9,098 |
| hpv_direct_colpo_3_8 | 291,377 | 22,075 | 4,287 | 298 | 68 | 512,129 | 602 | 345 | 9,068 |
| hpv_direct_colpo_3_9 | 289,274 | 21,707 | 4,122 | 305 | 68 | 506,341 | 595 | 345 | 8,948 |
| hpv_direct_colpo_4_1 | 389,476 | 40,500 | 5,572 | 232 | 52 | 794,475 | 668 | 361 | 9,645 |
| hpv_direct_colpo_4_10 | 375,967 | 23,401 | 4,370 | 288 | 54 | 609,981 | 612 | 359 | 9,192 |
| hpv_direct_colpo_4_11 | 387,635 | 18,099 | 4,167 | 327 | 70 | 568,623 | 573 | 344 | 8,729 |
| hpv_direct_colpo_4_12 | 386,467 | 17,622 | 4,155 | 327 | 67 | 562,687 | 573 | 346 | 8,745 |
| hpv_direct_colpo_4_13 | 384,135 | 17,022 | 4,032 | 326 | 62 | 554,358 | 574 | 351 | 8,757 |
| hpv_direct_colpo_4_14 | 383,656 | 16,522 | 4,056 | 331 | 63 | 548,878 | 569 | 350 | 8,719 |
| hpv_direct_colpo_4_15 | 378,445 | 15,512 | 3,825 | 342 | 62 | 533,568 | 558 | 351 | 8,631 |
| hpv_direct_colpo_4_16 | 374,152 | 15,283 | 3,782 | 350 | 64 | 526,983 | 550 | 349 | 8,585 |
| hpv_direct_colpo_4_2 | 387,384 | 40,188 | 5,453 | 231 | 46 | 789,261 | 669 | 367 | 9,664 |
| hpv_direct_colpo_4_3 | 384,425 | 39,859 | 5,350 | 242 | 49 | 783,014 | 658 | 364 | 9,561 |
| hpv_direct_colpo_4_4 | 382,983 | 38,916 | 5,155 | 260 | 53 | 772,143 | 640 | 360 | 9,358 |
| hpv_direct_colpo_4_5 | 376,225 | 36,232 | 4,551 | 320 | 65 | 738,545 | 580 | 348 | 8,831 |
| hpv_direct_colpo_4_6 | 389,107 | 27,299 | 5,023 | 243 | 53 | 662,096 | 657 | 360 | 9,534 |
| hpv_direct_colpo_4_7 | 384,830 | 24,466 | 4,706 | 267 | 49 | 629,495 | 633 | 364 | 9,399 |
| hpv_direct_colpo_4_8 | 381,878 | 24,163 | 4,627 | 274 | 49 | 623,505 | 626 | 365 | 9,356 |
| hpv_direct_colpo_4_9 | 377,577 | 23,927 | 4,569 | 285 | 54 | 616,845 | 615 | 360 | 9,269 |
| hpv_direct_colpo_5_1 | 488,885 | 53,346 | 6,333 | 172 | 43 | 1,022,346 | 728 | 371 | 10,030 |
| hpv_direct_colpo_5_2 | 484,611 | 50,525 | 6,030 | 194 | 37 | 989,862 | 706 | 376 | 9,937 |
| hpv_direct_colpo_5_3 | 485,783 | 46,440 | 6,154 | 186 | 38 | 950,186 | 714 | 375 | 10,000 |
| hpv_direct_colpo_5_4 | 478,422 | 42,053 | 5,696 | 213 | 36 | 898,948 | 687 | 377 | 9,814 |
| hpv_direct_colpo_6_1 | 587,079 | 57,482 | 6,783 | 142 | 36 | 1,161,904 | 758 | 377 | 10,244 |
| hpv_direct_colpo_6_10 | 569,277 | 51,828 | 6,089 | 187 | 30 | 1,087,560 | 713 | 383 | 9,996 |
| hpv_direct_colpo_6_11 | 580,511 | 50,661 | 6,527 | 158 | 28 | 1,087,120 | 742 | 385 | 10,216 |
| hpv_direct_colpo_6_12 | 575,843 | 48,058 | 6,287 | 172 | 27 | 1,056,419 | 728 | 386 | 10,123 |
| hpv_direct_colpo_6_2 | 494,459 | 54,281 | 6,325 | 167 | 45 | 1,037,271 | 733 | 368 | 10,014 |
| hpv_direct_colpo_6_3 | 409,494 | 49,382 | 5,866 | 207 | 52 | 903,311 | 693 | 361 | 9,744 |
| hpv_direct_colpo_6_4 | 583,574 | 56,409 | 6,650 | 141 | 28 | 1,147,664 | 759 | 385 | 10,275 |
| hpv_direct_colpo_6_5 | 498,161 | 51,493 | 6,194 | 180 | 35 | 1,013,089 | 720 | 378 | 10,033 |
| hpv_direct_colpo_6_6 | 577,884 | 54,908 | 6,457 | 154 | 27 | 1,126,968 | 746 | 387 | 10,200 |
| hpv_direct_colpo_6_7 | 583,099 | 55,912 | 6,683 | 144 | 29 | 1,142,215 | 756 | 384 | 10,265 |
| hpv_direct_colpo_6_8 | 583,023 | 54,812 | 6,674 | 151 | 31 | 1,131,145 | 749 | 383 | 10,238 |
| hpv_direct_colpo_6_9 | 579,439 | 53,783 | 6,493 | 158 | 27 | 1,117,264 | 742 | 386 | 10,200 |
| hpv_direct_colpo_7_1 | 682,747 | 59,931 | 7,079 | 132 | 33 | 1,282,060 | 768 | 380 | 10,311 |
| hpv_direct_colpo_7_10 | 675,616 | 58,592 | 6,898 | 125 | 20 | 1,261,535 | 775 | 393 | 10,403 |
| hpv_direct_colpo_7_11 | 668,286 | 57,705 | 6,711 | 135 | 21 | 1,245,337 | 765 | 392 | 10,338 |
| hpv_direct_colpo_7_12 | 582,841 | 52,793 | 6,254 | 173 | 28 | 1,110,773 | 727 | 385 | 10,090 |
| hpv_direct_colpo_7_13 | 675,521 | 58,200 | 6,909 | 127 | 20 | 1,257,523 | 773 | 393 | 10,392 |
| hpv_direct_colpo_7_14 | 670,616 | 57,358 | 6,761 | 134 | 20 | 1,244,191 | 766 | 393 | 10,351 |
| hpv_direct_colpo_7_15 | 668,011 | 52,087 | 6,597 | 149 | 21 | 1,188,883 | 751 | 392 | 10,274 |
| hpv_direct_colpo_7_2 | 499,121 | 53,901 | 6,470 | 165 | 41 | 1,038,130 | 735 | 372 | 10,089 |
| hpv_direct_colpo_7_3 | 680,657 | 59,712 | 7,049 | 124 | 24 | 1,277,777 | 776 | 389 | 10,406 |
| hpv_direct_colpo_7_4 | 503,025 | 51,745 | 6,263 | 177 | 34 | 1,020,473 | 723 | 379 | 10,050 |
| hpv_direct_colpo_7_5 | 680,540 | 59,590 | 7,043 | 123 | 25 | 1,276,435 | 777 | 389 | 10,397 |
| hpv_direct_colpo_7_6 | 679,248 | 59,414 | 7,004 | 121 | 22 | 1,273,383 | 779 | 391 | 10,419 |
| hpv_direct_colpo_7_7 | 676,106 | 59,031 | 6,908 | 124 | 20 | 1,266,420 | 776 | 393 | 10,413 |
| hpv_direct_colpo_7_8 | 584,304 | 55,281 | 6,546 | 148 | 25 | 1,137,111 | 752 | 388 | 10,237 |
| hpv_direct_colpo_7_9 | 678,376 | 59,058 | 6,988 | 123 | 22 | 1,268,958 | 777 | 391 | 10,412 |
| hpv_direct_colpo_8_1 | 774,733 | 61,716 | 7,230 | 115 | 21 | 1,391,896 | 785 | 392 | 10,449 |
| hpv_direct_colpo_8_10 | 587,707 | 53,044 | 6,324 | 171 | 27 | 1,118,149 | 729 | 386 | 10,114 |
| hpv_direct_colpo_8_11 | 498,756 | 51,177 | 6,101 | 187 | 34 | 1,010,527 | 713 | 379 | 9,973 |
| hpv_direct_colpo_8_12 | 495,883 | 50,918 | 6,033 | 193 | 37 | 1,005,065 | 707 | 376 | 9,915 |
| hpv_direct_colpo_8_13 | 765,033 | 60,417 | 7,046 | 116 | 16 | 1,369,206 | 784 | 397 | 10,461 |
| hpv_direct_colpo_8_2 | 683,222 | 59,820 | 7,078 | 122 | 23 | 1,281,427 | 778 | 390 | 10,416 |
| hpv_direct_colpo_8_3 | 590,814 | 56,766 | 6,747 | 137 | 27 | 1,158,473 | 763 | 386 | 10,306 |
| hpv_direct_colpo_8_4 | 505,815 | 51,862 | 6,292 | 176 | 34 | 1,024,438 | 724 | 379 | 10,055 |
| hpv_direct_colpo_8_5 | 771,780 | 61,475 | 7,200 | 114 | 18 | 1,386,528 | 785 | 396 | 10,471 |
| hpv_direct_colpo_8_6 | 588,124 | 55,454 | 6,594 | 147 | 25 | 1,142,668 | 753 | 388 | 10,254 |
| hpv_direct_colpo_8_7 | 771,703 | 61,453 | 7,195 | 112 | 17 | 1,386,231 | 788 | 396 | 10,482 |
| hpv_direct_colpo_8_8 | 769,872 | 61,294 | 7,167 | 113 | 16 | 1,382,809 | 787 | 397 | 10,481 |
| hpv_direct_colpo_8_9 | 765,386 | 61,004 | 7,108 | 117 | 16 | 1,375,429 | 783 | 397 | 10,466 |
| **hpv_direct_colpo_9_1** | **863,766** | **63,204** | **7,301** | **106** | **15** | **1,495,806** | **794** | **398** | **10,510** |
| hpv_direct_colpo_9_2 | 773,838 | 61,560 | 7,217 | 113 | 17 | 1,389,443 | 787 | 396 | 10,481 |
| hpv_direct_colpo_9_3 | 681,822 | 59,291 | 6,972 | 121 | 19 | 1,274,730 | 779 | 394 | 10,427 |
| hpv_direct_colpo_9_4 | 590,316 | 55,530 | 6,609 | 145 | 25 | 1,145,621 | 754 | 389 | 10,257 |
| hpv_direct_colpo_9_6 | 859,467 | 63,008 | 7,287 | 108 | 14 | 1,489,549 | 792 | 399 | 10,512 |
| hpv_direct_colpo_9_7 | 767,951 | 61,117 | 7,139 | 116 | 16 | 1,379,118 | 784 | 397 | 10,473 |
| hpv_direct_colpo_9_8 | 675,529 | 58,055 | 6,807 | 130 | 20 | 1,256,082 | 770 | 394 | 10,371 |
| hpv_direct_colpo_9_9 | 590,496 | 53,158 | 6,352 | 169 | 26 | 1,122,078 | 731 | 387 | 10,123 |
| no_screen | 0 | 0 | 0 | 900 | 413 | 0 | 0 | 0 | 0 |

# Model description

## Model purpose

Cervical screening can be used as a strategy to reduce both cervical cancer incidence and cervical cancer mortality.^1-3^ Cervical screening has been implemented in many different ways across the developed world. To assess the costs and effects of such screening programs one could monitor the existing programs or set up trials to evaluate different screening strategies. However, setting up large trials is expensive, needs a long follow-up time and might have ethical concerns. Also, the outcomes of such trials will be dependent on several factors, which might be different across countries, so the results might not be applicable to another country. Alternatively, modelling studies can be conducted to evaluate or predict cervical screening outcomes. MIcrosimulation SCreening Analysis (MISCAN) Sexually Transmitted Diseases Simulation (STDSIM) is a hybrid model. It contains a dynamic transmission component (STDSIM) that is able to estimate human papillomavirus (HPV) vaccination effects, and a microsimulation component (MISCAN) that is able to simulate a hypothetical population, including the development of cervical cancer. In these simulations, different screening strategies can be applied to quantify both the effects of screening in this population concomitantly with the effects of HPV vaccination.^4^ Because the population characteristics in the model and their background risk for cervical cancer can be tailored to those of a specific country, it is possible to make country-specific estimations of costs and effects for different screening strategies. In this project, the model was set to represent the situation in Ontario, Canada.

A hybrid model was used consisting of two, well-established individual based models for HPV transmission and vaccination, and cervical screening respectively. The STDSIM model describes the transmission of HPV, by subtype, through a dynamic network of sexual contacts, and will be used to determine the impact of current and future vaccination strategies and coverage levels for Ontario, Canada. In turn, age-, cohort- and type-specific HPV incidence reductions for each vaccination strategy will feed directly into MISCAN-cervix, in which the impact of HPV vaccination on cervical outcomes can be determined, and a wide range of comprehensive screening strategies can be evaluated. Both models are described in detail below. This combination of the two models coins the combined MISCAN -STDSIM -Cervix hybrid model. Note that the STDSIM part explicitly models inter-sex contacts and terminology of men/women is thus used here, while the MISCAN part models the disease progression of cervical cancer, applicable to any individual born with a cervix.

The main aims of MISCAN-STDSIM- Cervix are to quantify the effects of HPV-vaccination on cervical cancer epidemiology in the Ontario population and to evaluate the costs, harms and benefits of different potential screening strategies in this dynamic vaccination context.

## STDSIM model overview

STDSIM is an established stochastic microsimulation model of the transmission and control of human immunodeficiency virus (HIV), HPV, and other sexually transmitted infections (STIs).^5-8^ The model simulates the life course of individuals in a dynamic network of sexual contacts. Events like partnership formation or the acquisition of infections are the result of random processes, determined by probability distributions. Therefore, the results of the model are subject to real-world stochastic variation. A very important feature of STDSIM is that the transmission of different STIs (e.g. different HPV subtypes) and interventions (e.g. condom use, vaccinations, or treatments) can be simulated simultaneously. In addition, by explicitly simulating sexual network dynamics within an individual based model, the model allows for accurately incorporating herd immunity effects of prevention interventions such as vaccination directly.

The model consists of four modules: demography, sexual behavior, transmission and natural history, and interventions. The demography module implements the processes of birth, death, and migration. Processes for initiation and dissolution of sexual relationships, for mixing according to age preference, for sexual contacts within relationships and for once-off contacts in high-risk groups are defined in the sexual behavior module. In the transmission and natural history module, transmission probabilities per sexual contact are specified for all STIs in the model: HPV, HIV, chlamydia, gonorrhea, syphilis, chancroid, and HSV-2. Finally, the interventions module specifies the timing and effectiveness of (multiple) control measures in curbing transmission or enhancing survival. For the purpose of simulating the vaccine impact on HPV prevalence in Ontario, Canada, we will only simulate the four HPV types: HPV16, HPV18, HPVh5 (combination of types 31, 33, 45, 52, and 58), and HPVoHR (combination of all other high-risk types).

*Demography*

Demographic processes that result in a dynamic population of individuals in STDSIM comprise of: 1) birth; 2) mortality; and 3) migration (Figure S2). Births are assigned randomly to sexually active women from age 15 up to and including 49, where the probability of having a child depends on the age of the woman. At birth, the age at (non-HIV) death of each individual is drawn from pre-defined, sex-specific survival curves. Finally, the model also takes into account both directions of migration. Individuals can be removed from the population through age and sex-specific outmigration probabilities and clones of existing people are added to the population at age- and sex-specific rates to model immigration.


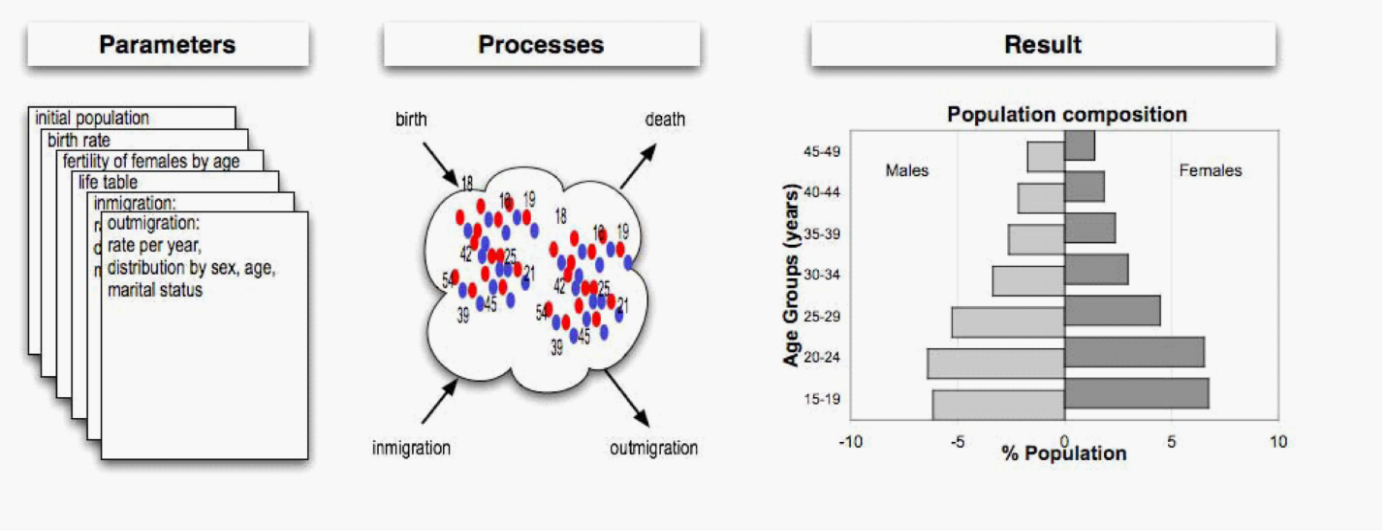


**Figure S2.** Demographic mechanisms in STDSIM

*Sexual behavior*

The model contains three types of sexual relationships: steady relationships, casual relationships, and one-off contacts. All relationships are formed between two people of opposite sex, and only penetrative vaginal sex acts are simulated in the model, we do not consider oral-genital or anal transmission of HPV. The formation of partnerships occurs according to a supply- and demand-based mechanism. People become available for a sexual relationship at an age of sexual debut, which is randomly drawn at birth from a uniform distribution. Each time the partnership status of a person changes (e.g. a partnership is formed or ended), a new duration until the person becomes available for a new relationship (time until availability) is drawn from a predefined exponential distribution with µ being the mean time until availability defined as:

µ= τ_s,r_/(r_s,a_ × *p*)

With: τ _s,r_ = time interval by person’s sex (s) and relationship status (r)

r_s,a_= specific partner change factor by sex (s) and age (a)

*p* = personal partner change factor

The personal partner change factor (*p*) reflects the heterogeneity in the tendency to form partnerships between individuals and is given by a gamma distribution with an average value (pm) of 1.0, and a situation specific shape parameter.

The duration of the availability period of an individual is given by an exponential distribution, with mean time to find (κ) defined as: δ/( r_s,a_ × *p*), where the δ is an average duration of the availability period. R_s,a_ and *p* are explained above. When a person is available for a new relationship, he/she can be selected by an individual of the opposite sex who has ended his/her availability period. If a person is not selected at the end of the availability period, he/she will select a partner from the pool of available persons of the opposite sex.

The type of relationship (steady or casual) that is formed when a partner is selected depends on the age of the male partner, and is defined as a probability of a steady relationship. The probability of a new relationship being a casual relationship is given by 1 – probability of a steady relationship. A relationship starts with a sexual contact. After each contact, the time until a new sexual contact within the relationship is drawn from an exponential distribution with a mean frequency of sexual contact depending on relationship type and the age of the male partner. Finally, the duration of a new relationship is drawn from an exponential distribution, where the average relationship duration is dependent on the relationship type.

Partner selection, which happens at the end of the availability duration, is guided through an age preference matrix, which defines the probability of selecting a partner from a certain age class. When there is no partner available in the preferred age class, immediate re-sampling is done of a new preferred age-class using the remaining age groups with a probability larger than 0.0. If no partner can be found in any of the age-classes, a new time to find is drawn from the above described equation. Probabilities in the age-preference matrix are chosen to have men prefer slightly younger women. The above described mechanisms of partnership formation result in a dynamic sexual network in the population (Figure S3), and can flexibly describe various mixing patterns. In general, the model will contain a heterogeneous mix of people with varying degrees in partner change rates, in which very active people (e.g. multiple and/or overlapping recent sexual partners) or very inactive (e.g. zero sexual partners for extended periods of time) can occur simultaneously.


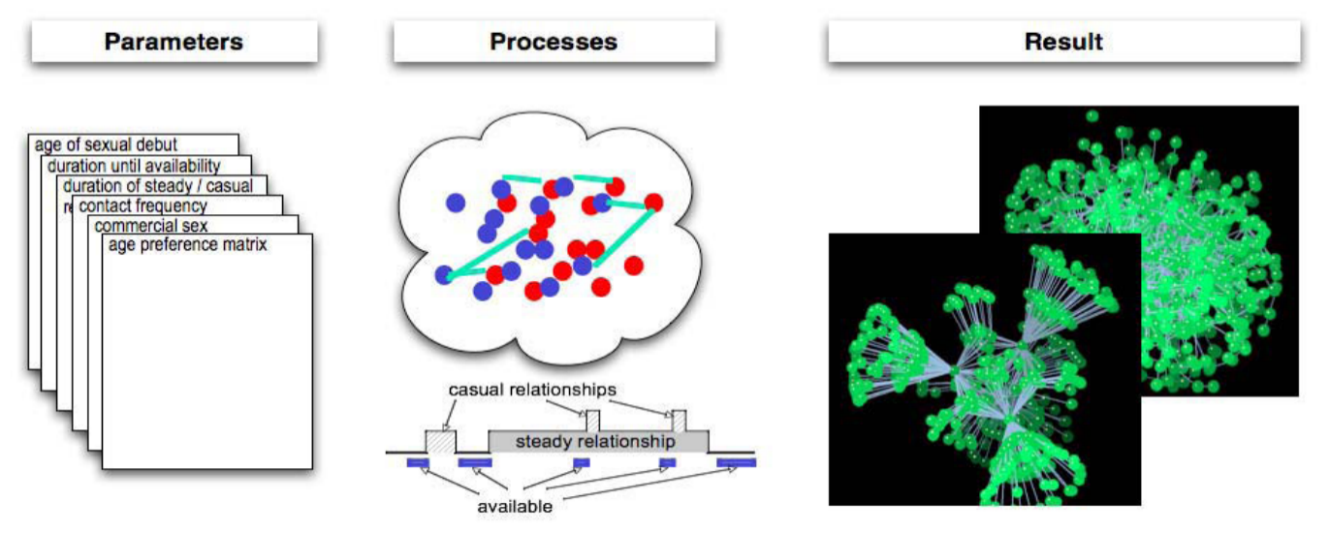


**Figure S3.** Mechanisms of sexual behavior in STDSIM create a dynamic sexual network

In the model, individuals can also have once-off contacts with individuals of the opposite sex (i.e. one-night stands or commercial sex), again through a supply- and demand-based mechanism. The mechanism was originally developed to describe commercial sex, and works as follows: A male’s frequency of visiting a female sex worker for a once-off contact is determined by defining frequency classes (e.g. 0, 1, and 12 times per year). For each class, the proportion of men with and without a steady relationship falling in that category can be specified. A personal sex-worker-visiting propensity (ranging from 0 to 1, assigned to each male at birth) determines which individual males are assigned to which frequency classes. At sexual debut and at each visit, the next visit is scheduled according to an exponential distribution with the mean duration until next visit based on the visit frequency of the individual.

The number of women participating in the high-risk network in the model results from the male demand. Women are recruited from sexually active females with a defined age range, and the number of available women for once-off contacts and their predefined number of contacts per week is checked each year and matched with the total male-driven demand for once-off contacts. If the number of women is too low, new women are recruited. If the number is too high, a random selection stops participating in the high-risk network. Males and females participating in the network are always part of the general population, which means that they are also part of the general sexual network and can form partnerships.

This mechanism can represent either commercial sex dynamics, high-risk once-off contacts in the general population, or both. The model allows for flexibly defining the number of contacts per male and female, and defining different relationships. For instance, when defining contact rates that are the same for men and women, the model reproduces once-off contact dynamics (i.e. one-night stands).

*Transmission and natural history*

The transmission and natural history module specifies the duration of the different disease stages, transmission rates, susceptibility, and natural immunity processes. Transmission in STDSIM is simulated as a per-act probability of transmission in a sero-discordant sex act, based on a variety of promoting and inhibiting factors. For instance, natural immunity due to clearing a previous HPV infection will reduce the susceptibility of the uninfected partner in a sero-discordant sex act. For HPV, we specify type-specific transmission probabilities, durations, and immunity development processes. We have previously quantified natural history parameters for HPV in The Netherlands^4, 9-11^ and the United States (US) (manuscripts in preparation). For the purpose of the current study, we assume natural history in Ontario to be the same as applied for the US.

*Interventions*

The interventions module flexibly handles a range of possible interventions that can be simulated simultaneously. It encompasses interventions that change people’s sexual behavior, such as delay of sexual debut, partner reduction, and abstinence. It also includes interventions that change the transmission probability during sex (such as condom use), and encompasses a wide range of possible vaccination and screening options. For instance, routine mass vaccination of (pre-)adolescent children can be simulated, with varying and age- or sex-specific participation rates, alongside opportunistic adult vaccination based on a negative HPV screen test result or high-risk behavior.

## STDSIM quantifications

For STDSIM, we have already calibrated the model to the US setting. We expect it will not be necessary to change any modelling assumptions, except for the use of Ontario-specific vaccine uptake data.

Our original HPV transmission model was first developed for the Netherlands, where we previously quantified and validated the sexual network extensively^11^. We calibrated per-act transmission probabilities, clearance rates, and natural immunity development to reproduce observed pre-vaccination HPV16 and HPV18 prevalence by age^9^. However, later studies showed that the data we used were likely an underestimation of actual HPV prevalence in The Netherlands^12, 13^. Therefore, we recalibrated our model to reproduce the US HPV prevalence levels as follows. First, we compared sexual behavior outputs of the model to data from the National Survey on Family Growth (NSFG)^14^ and National Health and Nutrition Examination Survey (NHANES)^15^ conducted by the Centers for Disease Control and Prevention in the US. We concluded that the network dynamics in our model relatively closely reproduced observed partner change rates in these data sources (Figure S4). The slight overestimation of the number of reported recent partners in women may be due to social desirability bias (i.e. the tendency to report desirable attributes), resulting in underreporting, especially among women. There are also clear discrepancies between numbers of lifetime partners between the two datasets for women, and we believed that, as an average of the total female population, NHANES data seems to be unrealistic with an average of more than 20 lifetime partners. As such, we were more comfortable with the data model closely matching the more realistic NSFG data on this indicator.

Second, we recalibrated natural history parameters to reproduce observed HPV prevalence levels in the US by repeatedly randomly sampling transmission probabilities, clearance rates, and natural immunity waning for HPV16, HPV18, HPVh5 and HPVoHR until 100 acceptable parameter combinations that closely reproduced observed HPV prevalence levels in the US^16^ were identified. By reproducing multiple sets of acceptable parameter combinations rather than a single ‘best fit’, our model incorporates parameter uncertainty in its predictions. The resulting predicted and observed age- and type-specific HPV prevalence levels are given in Figure S5.


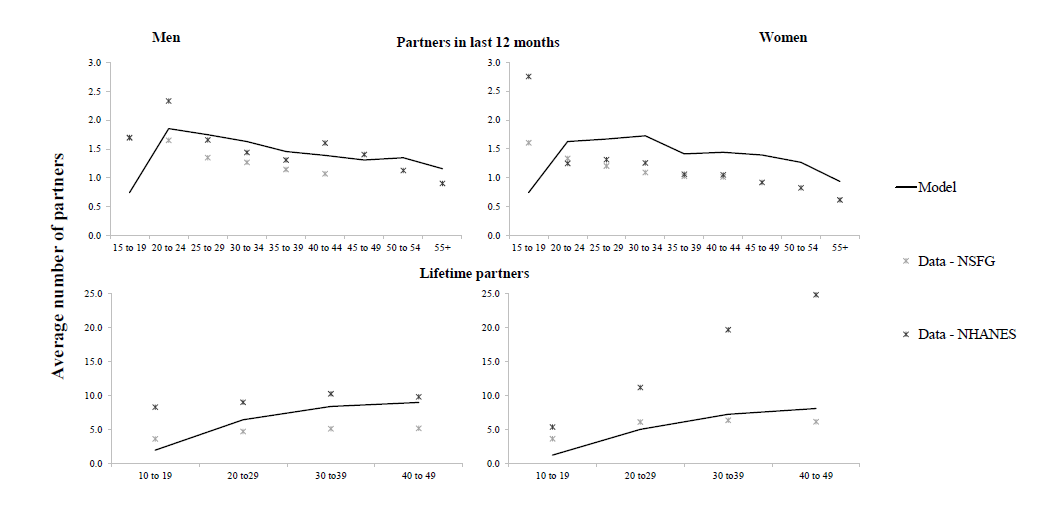
**Figure S4.** Comparison of the sexual behavior outputs of the STDSIM model to NHANES - National Health and Nutrition Examination Survey (2011-2012) and NSFG [- National Survey of Family Growth (2011-2012)](https://www.cdc.gov/nchs/nsfg/index.htm)


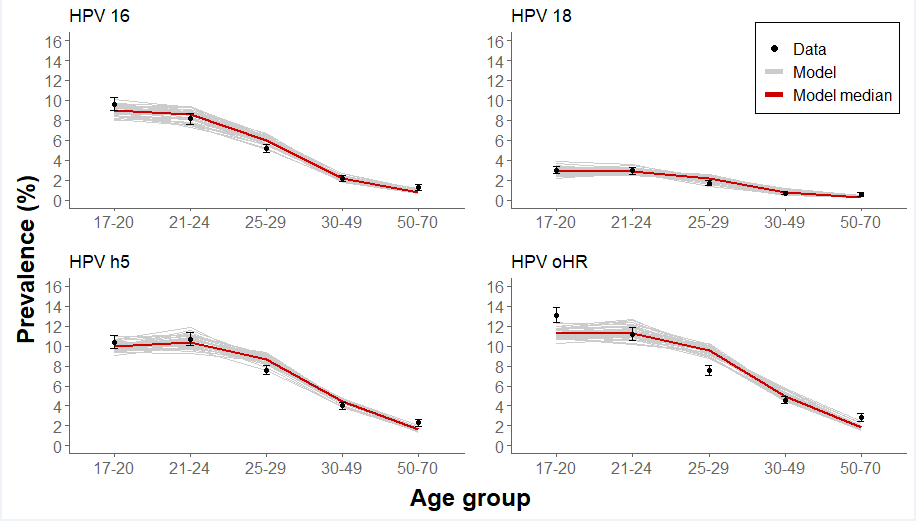


**Figure S5.** Predicted versus observed HPV prevalence levels in women in the US, pre vaccination.

## MISCAN model overview

The Microsimulation Screening Analysis (MISCAN) program was first developed in 1985 by the Department of Public Health of Erasmus MC University Medical Center in The Netherlands to evaluate the effects of screening on disease.^4^ Since then, the MISCAN program has been used to quantify the effects of screening for cancers of the breast, colon, cervix, prostate, and lung.^17-21^

MISCAN-Cervix, originally coded in Borland Delphi 7 and recently transferred to Python 3, is a stochastic, semi-Markov microsimulation model. In a microsimulation model, individuals are simulated one at a time instead of as proportions of a cohort. The advantage of this is that new events can be dependent on past events of that individual. The model is stochastic, which means that sequences of events are simulated by drawing from distributions of probabilities and durations instead of using fixed values. Therefore, the outcomes of the model are subject to random variation.

### Model description

Figure S6 shows the basic structure of MISCAN-Cervix. The program consists of three main parts:

1. Demography
2. Natural history
3. Screening

Input data for these parts is processed by the MISCAN program to generate individual life histories of individuals with a cervix within a population. The program simulates both a situation without screening and a situation with the selected screening strategy. The difference between the outcomes of those two scenarios is considered as the effect of screening. All three parts are described further in the upcoming sections.


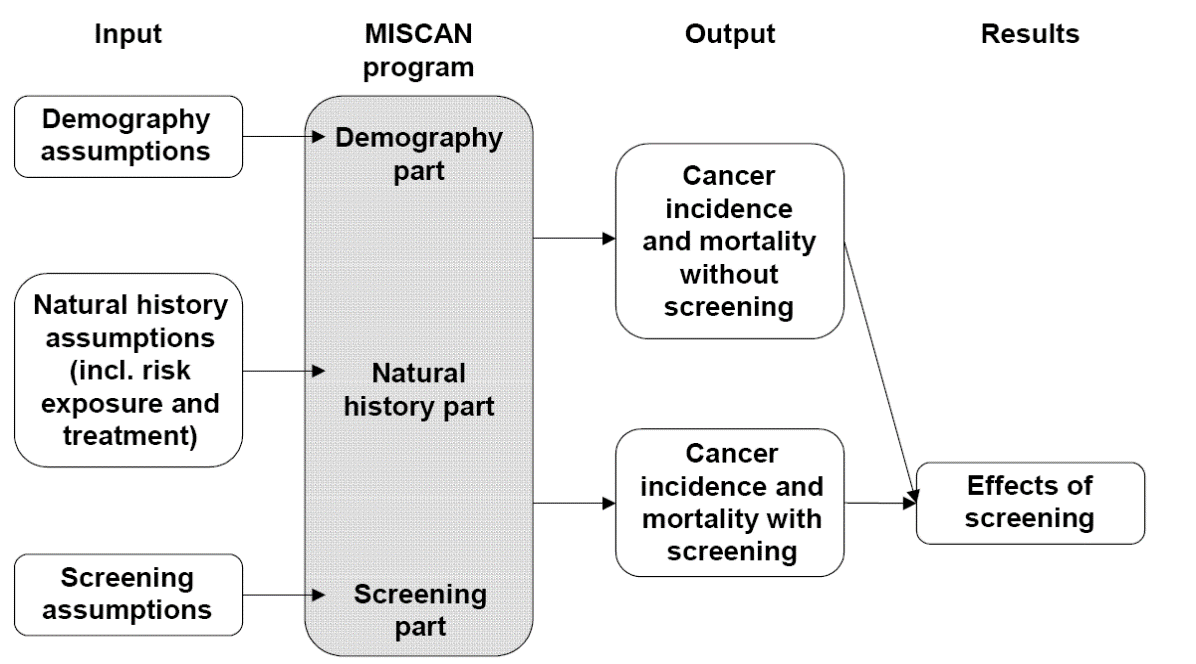


Figure S6. Basic structure of MISCAN-Cervix

### Demography

MISCAN-Cervix simulates a population of individuals born with a cervix. A specific life history is generated for each individual, including a date of birth and date of death from other causes than cervical cancer. Simulated individuals have a probability to have a hysterectomy for reasons other than cervical cancer, depending on the age and the birth year of the individual of interest. Modelled individuals cannot become older than 100 years.

### Demography assumptions

The following demography assumptions were made, which affect the characteristics of the population without the presence of cervical cancer:

- Multiple cohorts are simulated to describe the Ontario population. The simulated individuals are born in different years and will die from cervical cancer or from other causes at different moments in time.
- In the model, it is assumed that death from cervical cancer is independent from death from other causes. Whichever comes first determines the actual moment of death.
- Individuals who get a hysterectomy in the model have their cervix removed completely, can no longer develop HPV infections, precancerous lesions or cancers and no longer participate in screening.

### Natural history

Figure S7 shows the natural history structure of MISCAN-CERVIX. In the static MISCAN model, multiple health states with corresponding durations and transition probabilities are defined.

Each individual starts at the ‘normal’ state and has an individual background risk of acquiring one or multiple infections with a high-risk type of the human papillomavirus (hrHPV). This risk is determined by STDSIM outputs and is dependent on age, vaccination status, cohort and hrHPV type. The hrHPV types can cause cancer and are detectable by the hrHPV-test. The model distinguishes between four categories of hrHPV types, based on their likeliness to cause cancer and efficacy of different vaccines against these types:

1. HPV-16
2. HPV-18
3. High risk types that are covered in the nonavalent vaccine (HPV-31/33/45/52/58)
4. Other high risk HPV types (HPV-35/39/51/56/59/66/68)

An acquired hrHPV infection will most likely regress, but may progress to a pre-invasive cervical intraepithelial neoplasia (CIN) grade 1, which may progress sequentially to CIN grade 2 and 3. Regression probabilities of pre-invasive lesions are dependent on age and lesion grade. An individual might also develop CIN lesions without the presence of hrHPV, although hrHPV-negative lesions will never progress to cancer.^22^ An individual can have multiple lesions and hrHPV infections simultaneously, which may regress or progress independent of each other.^23^

A CIN3 lesion may progress to cervical cancer, which is modeled in five different stages according to the Fédération Internationale de Gynécologie et d'Obstétrique (FIGO) classification: FIGO stages 1A, 1B, 2, 3 and 4.

Cancers classified as FIGO stage 1B and higher may be detected clinically (i.e., because of symptoms and/or signs) before progressing to a higher stage. Screening is able to detect all types of pre-invasive and invasive lesions, with different type-specific sensitivities. Survival probabilities and durations until cervical cancer death depend on the stage and on the age at which the cancer is detected. A death is only counted as a cervical cancer death if the individual dies from cervical cancer before dying from other causes.

An individual might die of other causes than cervical cancer at any moment during their lifetime, irrespective of existing cervical (pre)cancers. Also, an individual might have a hysterectomy for other reasons than cervical cancer at any moment, removing all prevalent hrHPV infections and CIN lesions. Individuals who have had a hysterectomy will no longer be at risk for acquiring a cervical hrHPV infection or CIN lesion and will not be invited for screening any more.

**Figure S7** The natural history structure of MISCAN-Cervix

(hr)HPV = (high-risk) human papillomavirus; CIN = cervical intraepithelial neoplasia; FIGO = International Federation of Gynaecology and Obstetrics.

If an individual acquires an hrHPV infection, the prevalence of disease is added to the original life history of this individual generated in the demography part, resulting in a life history with disease, as shown in Figure S8. In the case summarized in Figure S8 below, the individual acquires two hrHPV infections. The first infection will progress to a CIN2 before the infection clears and the lesion regresses, while the second infection will progress to cervical cancer and cause a cervical cancer death. In the bottom line, the original life history is combined with the natural history of cervical cancer. Here we see that the moment of death from cervical cancer occurs before the moment of death from other causes, altering the original moment of death. The difference between the original moment of death from other causes and the moment of death from cervical cancer is the number of life years lost due to cervical cancer.

**Life history without cervical cancer (demography part)**

Birth

Death from other causes

**Development of first HPV infection**

CIN1

CIN2

HPV

Regression

**Development of second HPV infection**

Death from cervical cancer

Preclinical FIGO 1A

Clinical FIGO 1B

Preclinical FIGO 1B

CIN3

CIN2

CIN1

HPV

**Combined life history for cervical cancer**

CIN1

HPV

Death from cervical cancer

Preclinical FIGO 1A

Clinical FIGO 1B

Preclinical FIGO 1B

CIN3

CIN2

Birth

🡨 🡪 Life years lost

Figure S8. Life histories with and without disease

HPV = human papillomavirus; CIN = cervical intraepithelial neoplasia; FIGO = International Federation of Gynaecology and Obstetrics.

### Natural history assumptions

Many characteristics of the natural history of cervical cancer cannot be observed because the disease starts to develop unnoticed. Once a diagnosis is made, it is in most cases unethical not to intervene. Therefore, assumptions have to made about the natural history of cervical cancer. These assumptions are based on expert opinion or derived from observed data such as detection rates. For an overview of the natural history part of the model, please see the natural history section of the model overview.

**Human Papillomavirus**

- Each individual has an age-specific risk of acquiring hrHPV infections.
- An individual can acquire multiple hrHPV infections during their lifetime, and these hrHPV infections may be present at the same time. The progression of these lesions are modelled independently, there is no interaction.
- If vaccination is introduced, there will be an age-specific relative reduction of the age-specific risk of acquiring hrHPV infections, depending on the vaccination type and vaccination coverage.
- Most hrHPV infections will clear naturally before progressing to CIN.
- As described in the natural history section of the model overview, the model distinguishes four categories of hrHPV genotypes. The duration of hrHPV-infections and subsequent CIN lesions are assumed equal for all genotypes and independent of age. However, the progression probabilities from all pre-invasive health states are different between all genotypes and are dependent on age as well.
- If an individual has a hysterectomy because of cervical cancer or for other reasons than cervical cancer, all cervical hrHPV infections are considered removed as well. No new hrHPV cervical infections can be acquired.

**Cervical Intraepithelial Neoplasia (CIN)**

- Most CIN1 lesions will develop from an hrHPV infection
- Each individual has an age-specific risk of developing a CIN1 lesion in the absence of hrHPV.
- Progression probabilities for CIN lesions depend on lesion grade, age and hrHPV genotype. Most CIN1 lesions will clear before progression to CIN2. Those that progress to CIN2 will mostly clear before progression to CIN3. hrHPV-negative CIN3 will never progress to cancer.^22^
- If an individual has a hysterectomy because of cervical cancer or for other reasons than cervical cancer, all CIN lesions are considered removed as well. No new CIN lesions can be developed.

**Cervical cancer**

- Cervical cancer can develop only following a hrHPV-positive CIN3 lesion.
- After the detection of cervical cancer, the individual has a hysterectomy. Therefore, we do not assume any possibility of having a recurrent cervical cancer.
- Preclinical FIGO1A does not cause symptoms yet and will therefore never be clinically detected. Preclinical FIGO1B or higher stages can be detected clinically in the absence of screening or can progress to a higher cancer stage (Figure S7).
- Durations of the different cancer stages do not depend on age or genotype
- Once a lesion has become cancer, progression probabilities to higher cancer stages depend on age, but are equal across genotypes.
- Clinically detected cervical cancer can either be cured or cause cervical cancer death. The probability of dying from cervical cancer is dependent on the cancer stage and the age of the individual.
- If the individual is cured they will stay in the cancer state until death from other causes. If the individual is not cured, they will die of cervical cancer within a maximum of 10 years after diagnosis.

**Hysterectomy**

- Individuals who do not have cervical cancer have an age-specific probability of getting a hysterectomy for reasons other than cervical cancer.
- A hysterectomy is assumed to remove all prevalent hrHPV cervical infections and CIN lesions.
- Individuals who have had a hysterectomy will no longer acquire new hrHPV infections or develop new CIN lesions and will no longer be invited for screening tests

### Screening part

In the screening part of MISCAN-Cervix, screening strategies and screening behaviour are simulated. Individuals can be invited to participate in screening at specified ages. Depending on the test used and the highest prevalent lesion of an individual at the moment of the screening test, there is a probability of a positive test result. If the screening test is positive, an individual will be referred for either a triage test or a referral to colposcopy, depending on the applied strategy. When an individual is referred to colposcopy and they adhere to this referral, it is assumed that all prevalent CIN lesions will be diagnosed and successfully removed/treated. In practice, low grade pre-invasive lesions (e.g. CIN1) might not be treated directly, but as these individuals will be monitored regularly and treated if the lesion progresses, these low grade lesions are considered as removed in the model.

As screen-detected cancers tend to have a better stage-specific survival than clinically detected cancers, detection of cervical cancer by screening in the model may prevent death from cervical cancer. However, if the death from cervical cancer is not prevented, the duration from the moment of detection until the moment of death from cervical cancer will not be different from clinically detected cancers.

The effect of screening on the life history of an individual is shown in Figure S9. In this case, the individual attends a screening intervention, removing the prevalent CIN lesions. The second hrHPV infection will not lead to cancer anymore and therefore, the individual will die at the original moment of death from other causes. The difference between the moment he or she would have died from cervical cancer and the moment he or she will now die of other causes is the screen effect and can be quantified as the number of life years gained.

**Life history without cervical cancer**

Birth

Death from other causes

**Development of first HPV infection**

CIN1

CIN2

HPV

Regression

**Development of second HPV infection**

Death from cervical cancer

Preclinical FIGO 1A

Clinical FIGO 1B

Preclinical FIGO 1B

CIN3

CIN2

CIN1

HPV

**Combined life history for cervical cancer**

🡨 🡪 Life years gained

No CIN or cancer present

Death from other causes

CIN1

HPV

CIN3

CIN2

Birth

**Screening intervention**

Figure S9. Life history with screening

HPV = human papillomavirus; CIN = cervical intraepithelial neoplasia; FIGO = International Federation of Gynaecology and Obstetrics.

### Screening assumptions

Several assumptions have to be made regarding the performance of the screening tests, the consequences of colposcopy and the screening behaviour of the population. For an overview of the screening part of the model, please see the screening section of the model overview.

**Terminology**

- The screening strategy of an organised screening program determines between which ages individuals are invited for screening, with which interval they are invited, which primary test is performed and which triage tests are performed after a positive primary test.
- A primary test is the initial screening test an individual is invited to. Based on the result of this test, the individual will be referred to colposcopy or triage testing.
- The primary test can either be cytology (checking for abnormal cells), hrHPV-test (checking for the presence of hrHPV) or a co-test, which is a combination of both.
- A triage test is a screening test that is performed after an individual has had a positive primary screening test, but before the decision is made whether or not to refer them for a colposcopy (e.g. a cytology test after a positive primary hrHPV-test). The triage test can be performed either directly after the primary test, or after a waiting period of several months or years, depending on the screening strategy.
- A colposcopy is a diagnostic exam by a trained medical professional to determine the presence or absence of disease. This might include taking a biopsy.

**Performance of the screening tests**

- The probability of having a positive test result depends on the lesion grade and the hrHPV status of the individual for both cytology and the hrHPV-test.
- No differences in test characteristics are assumed for different hrHPV genotypes, both for cytology and the hrHPV-test.
- Systematic positive and systematic negative test results over time are possible for cytology for certain individuals, infections or lesions.

**Screening behaviour**

- Individuals invited to screening can either attend or not attend the primary screening test. The probability to attend is dependent on age. If an individual attends, he or she will do so exactly at the invited age.
- If an individual attends the primary test and is referred to triage testing or colposcopy, he or she might not adhere to this referral.
- If a scenario is simulated where the age ranges of the screening program are extended compared to the current target ages, individuals in those newly targeted age groups will attend with the same probability as the closest age group that is invited in the current screening program.

**Colposcopy**

- When an individual is referred to colposcopy, all prevalent CIN lesions will be diagnosed and removed/treated.
- Colposcopy is 100% accurate and will show the highest prevalent lesion.
- Individuals with a prevalent hrHPV infection but without a prevalent CIN will not be treated. The hrHPV infection may still progress to CIN after the colposcopy.
- Early detection of cervical cancer by screening in the model may prevent death from cervical cancer. However, if the death from cervical cancer is not prevented, the duration until death from cervical cancer will not be different from clinically detected cancers.

## Model output

After the simulations are complete, several types of outcomes can be reported by the program, such as:

### STDSIM outputs

1. HPV incidence by
   1. Type
   2. Age
   3. Year
   4. Vaccination status

### Demographic or epidemiological outputs

1. Total number of life-years lived
2. Cervical cancer incidence rates by age group
3. Cervical cancer mortality rates by age group
4. Cervical cancer incidence counts by age group
5. Cervical cancer mortality counts by age group
6. Number of cervical cancer deaths per 100 000 individuals simulated lifelong

### Screening outputs

1. Number of screen tests and triage tests by age group
2. Number of false positive referrals to colposcopy
3. Number of referrals to colposcopy by lesion grade and by age
4. Cervical cancer mortality reduction due to screening
5. Number of primary tests needed to prevent 1 cervical cancer incidence or death
6. Number of colposcopies needed to prevent 1 cervical cancer incidence or death
7. Life-years gained by screening

### Harms and Benefits of screening

1. Harms of screening
2. Life-years gained compared to a no screening scenario
3. Cancers prevented compared to a no screening scenario
4. Harms / cancer prevented

## MISCAN model parameters

Next to the assumptions on the structure of the model, calculations or assumptions have to be made to determine the exact values of certain model parameters to reflect a given population, in this case, for Ontario. In this section, we will describe the types of parameters that serve as inputs for the model and how the values of these parameters are determined. The parameters are categorised by the part of the model they belong to. Please see the model overview section (3) above for more information about the model parts. Some parameters were calibrated. Please see the calibration section (6) for more information about the calibration process. A summary table of all model parameters as well as respective data used to set or estimate them can be found in Section 8.

### Demographic part

The model simulates a sample population of people with a cervix, representing the current Ontario population. All individuals have a probability to die at each age as reported by the all-cause female mortality statistics for Ontario in 2010-2017 provided, which results in an average life expectancy of around 84 years. Ontario age-specific hysterectomy probabilities by age were also obtained through Ontario Health Insurance Plan (OHIP) data for the years 2008-2018.

### Natural history part

*Background risk*

The age-specific background risk for acquiring an hrHPV infection will be calibrated to the hrHPV-prevalence as observed in the Montreal^24^ and British Columbia^25^ population, since detailed Ontario data was not available (see Appendix B). The age-specific distribution of hrHPV infections over the four categories of genotypes will be based on Wheeler et al. 2014.^26, 27^

*Progression probabilities of pre-invasive lesions*

The age-specific probabilities that an hrHPV infection will progress to CIN1, CIN2, CIN3 or cancer are estimated during the calibration process using mainly detection rates of the current Ontario screening program as a calibration target (see calibration section).

*Probabilities of clinical detection of cancer*

The age-specific probability that a FIGO1B, FIGO2 or FIGO3 cancer will be clinically detected before it progresses to a higher cancer stage is obtained through the calibration process where the cancer stage distribution (provided by Ontario Health) was the main calibration target. FIGO1A is assumed not to give symptoms, so it will not be clinically detected, whilst FIGO4 cannot progress to a higher cancer stage, so will always be clinically detected at that stage.

*Duration of health states*

Most health states in the model have a duration, before transition to a next health state, that is drawn from a Weibull distribution. Most of these distributions have a Weibull shape parameter of 1, making them an exponential distribution (Table S5). The duration of clinical cervical cancer (states 34-37) and death from cervical cancer, if an individual is not cured, is assumed to be less than ten years and based on stage-specific survival data from the Ontario Cancer Registry (OCR).

**Table S5.** Durations of health states

| Transition number* | From state* | To state* | Mean duration (years) | Weibull shape | Source |
| --- | --- | --- | --- | --- | --- |
| 1 | 4. HPV-OHR | 1. Normal | 1 | 1 | ^28, 29^ |
| 2 | 4. HPV-OHR | 9. HPV-OHR CIN1 | 1 | 1 | ^28, 29^ |
| 3 | 5. HPV-9V | 1. Normal | 1 | 1 | ^28, 29^ |
| 4 | 5. HPV-9V | 10. HPV-9V CIN1 | 1 | 1 | ^28, 29^ |
| 5 | 6. HPV-18 | 1. Normal | 1 | 1 | ^28, 29^ |
| 6 | 6. HPV-18 | 11. HPV-18 CIN1 | 1 | 1 | ^28, 29^ |
| 7 | 7. HPV-16 | 1. Normal | 1 | 1 | ^28, 29^ |
| 8 | 7. HPV-16 | 12. HPV-16 CIN1 | 1 | 1 | ^28, 29^ |
| 9 | 8. NoHPV CIN1 | 1. Normal | 1.5 | 1 | ^30^ |
| 10 | 8. NoHPV CIN1 | 13. NoHPV CIN2 | 1.5 | 1 | ^30^ |
| 11 | 9. HPV-OHR CIN1 | 1. Normal | 1.5 | 1 | ^30^ |
| 12 | 9. HPV-OHR CIN1 | 14. HPV-OHR CIN2 | 1.5 | 1 | ^30^ |
| 13 | 10. HPV-9V CIN1 | 1. Normal | 1.5 | 1 | ^30^ |
| 14 | 10. HPV-9V CIN1 | 15. HPV-9V CIN2 | 1.5 | 1 | ^30^ |
| 15 | 11. HPV-18 CIN1 | 1. Normal | 1.5 | 1 | ^30^ |
| 16 | 11. HPV-18 CIN1 | 16. HPV-18 CIN2 | 1.5 | 1 | ^30^ |
| 17 | 12. HPV-16 CIN1 | 1. Normal | 1.5 | 1 | ^30^ |
| 18 | 12. HPV-16 CIN1 | 17. HPV-16 CIN2 | 1.5 | 1 | ^30^ |
| 19 | 13. NoHPV CIN2 | 1. Normal | 2 | 1 | ^30, 31^ |
| 20 | 13. NoHPV CIN2 | 23. NoHPV CIN3 | 2 | 1 | ^30, 31^ |
| 21 | 14. HPV-OHR CIN2 | 1. Normal | 2 | 1 | ^30, 31^ |
| 22 | 14. HPV-OHR CIN2 | 24. HPV-OHR CIN3 | 2 | 1 | ^30, 31^ |
| 23 | 15. HPV-9V CIN2 | 1. Normal | 2 | 1 | ^30, 31^ |
| 24 | 15. HPV-9V CIN2 | 25. HPV-9V CIN3 | 2 | 1 | ^30, 31^ |
| 25 | 16. HPV-18 CIN2 | 1. Normal | 2 | 1 | ^30, 31^ |
| 26 | 16. HPV-18 CIN2 | 26. HPV-18 CIN3 | 2 | 1 | ^30, 31^ |
| 27 | 17. HPV-16 CIN2 | 1. Normal | 2 | 1 | ^30, 31^ |
| 28 | 17. HPV-16 CIN2 | 27. HPV-16 CIN3 | 2 | 1 | ^30, 31^ |
| 29 | 23. NoHPV CIN3 | 1. Normal | 5.7 | 0.84 | Calibration |
| 30 | 24. HPV-OHR CIN3 | 1. Normal | 5.7 | 0.84 | Calibration |
| 31 | 24. HPV-OHR CIN3 | 28. FIGO1A | 14.3 | 0.84 | Calibration |
| 32 | 25. HPV-9V CIN3 | 1. Normal | 5.7 | 0.84 | Calibration |
| 33 | 25. HPV-9V CIN3 | 28. FIGO1A | 14.3 | 0.84 | Calibration |
| 34 | 26. HPV-18 CIN3 | 1. Normal | 5.7 | 0.84 | Calibration |
| 35 | 26. HPV-18 CIN3 | 28. FIGO1A | 14.3 | 0.84 | Calibration |
| 36 | 27. HPV-16 CIN3 | 1. Normal | 5.7 | 0.84 | Calibration |
| 37 | 27. HPV-16 CIN3 | 28. FIGO1A | 14.3 | 0.84 | Calibration |
| 38 | 28. Preclinical FIGO1A | 29. Preclinical FIGO1B | 4 | 1 | Calibration |
| 39 | 29. Preclinical FIGO1B | 34. Clinical FIGO1B | 2.2 | 1 | Calibration |
| 40 | 29. Preclinical FIGO1B | 30. Preclinical FIGO2 | 2.2 | 1 | Calibration |
| 41 | 30. Preclinical FIGO2 | 35. Clinical FIGO2 | 1.7 | 1 | Calibration |
| 42 | 30. Preclinical FIGO2 | 31. Preclinical FIGO3 | 1.7 | 1 | Calibration |
| 43 | 31. Preclinical FIGO3 | 36. Clinical FIGO3 | 1.7 | 1 | Calibration |
| 44 | 31. Preclinical FIGO3 | 32. Preclinical FIGO4 | 1.7 | 1 | Calibration |
| 45 | 32. Preclinical FIGO4 | 37. Clinical FIGO4 | 0.7 | 1 | Calibration |

* All possible states and transitions are graphically presented in Figure S7.

HPV=human papillomavirus OHR=Other high risk types 9V=HPV genotypes covered by the nonavalent vaccine, excluding HPV 16 and HPV18 CIN=Cervical intraepithelial neoplasia FIGO=Fédération Internationale de Gynécologie et d'Obstétrique..

### Screening part

*Current Program: The Ontario Cervical Screening Program (OCSP)*

For calibrating the model, the OCSP’s current state recommendations were used. This organized program was set up in 2000 and initially recommended annual cytology (Pap test) screening for people at average risk. From 2011, the screening interval was adjusted to 3-yearly cytology tests for people at average risk. In the current program, screening starts at age 21 and participants are invited every 3 years for cytology until the age of 69. Individuals with a high-grade (high grade squamous intraepithelial lesion [HSIL], atypical squamous cells cannot exclude HSIL [ASC-H], atypical glandular cells [AGC], or adenocarcinoma in situ [AIS]) cytology result are referred for colposcopy, while individuals with a lower grade positive cytology result were invited to a follow-up scheme with two repeat tests after 6 month intervals. If any of these repeat tests showed abnormal results they would also be referred to colposcopy. The program is described in more detail in Figure S10.


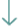

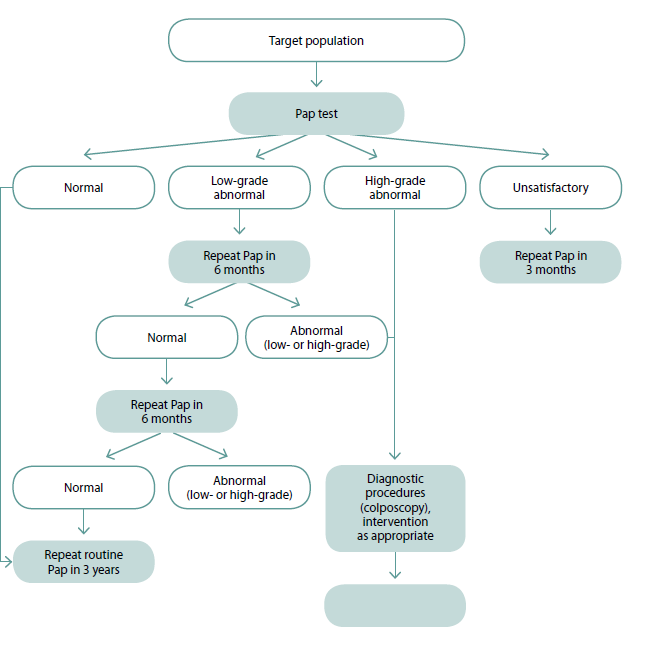


**Figure S10.** Triage in the current cytology program.

*Screening behaviour*

The age-specific probability to attend primary screening is based on observations in the current cytology screening program. The simulated probability to attend a primary cytology test was calculated using the observed proportion of individuals eligible for screening between 2008 and 2018 who had completed at least one cytology test in the previous 42 months. A certain proportion of the population is assumed to never attend the screening program and the observed attendance rates are adjusted for this. Next, the average attendance of the following 3 years is taken to determine the simulated participation rate of a certain year. The participation rate is then adjusted for yearly (pre 2011) and 3-yearly (2012 onwards) screening intervals as opposed to the 3,5 year window in data collection given the OCSP’s switch in recommended screening interval in 2011. For illustration of this calculation, the age-averaged attendance and participation rates for different calendar years are shown in Table S6. Note that in the model these values are both age and calendar year dependent. For the calendar years before 2008, the attendance is assumed to be equal to the 2008-2010 average The adherence to repeat tests and colposcopies is assumed to be constant over time and is defined as the average adherence of the specific surveillance measure from 2008 through to 2018. While the observed data is not truly constant over time, it remained at a relatively similar level to support this assumption.

**Table S6.** Modelled screening behaviour

| Screening behaviour | Cytology-based  screening program |
| --- | --- |
| Percentage never-attenders | 13.9% |
| *Average participation 2008-2011* |  |
| - observed attendance | 66.4% |
| - adjusted for never attenders | 77.2% |
| - adjusted for yearly interval | 39.5% |
| *Average participation 2012-2014* |  |
| - observed | 64.3% |
| - adjusted for never attenders | 74.6% |
| - adjusted for 3-yearly interval | 67.1% |
| *Average participation 2015-2018* |  |
| - observed attendance | 60.2% |
| - adjusted for never attenders | 69.9% |
| - adjusted for 3-yearly interval | 64.2% |
|  |  |
| *Adherence to repeat testing* |  |
| - 6 months after primary test | 79.6% |
| - 12 months after primary test | 66.1% |
| *Adherence to a referral for colposcopy after a* |  |
| - direct referral | 83.1% |
| - referral at 6 months after primary test (ASCUS/LSIL) | 83.1% |
| - referral at 18 months after primary test | 83.1% |

*Test characteristics*

The test characteristics in MISCAN-Cervix are presented in Table S7. The probabilities of a cytology test being positive are already accurately calibrated using Dutch detection rates and interval cancers. Test characteristics for the hrHPV-test are based on literature. The sensitivity of the hrHPV test for hrHPV-positive individuals with a ≥CIN2 lesion was found to be 94% in the POBASCAM study.^32^ The probability of a positive hrHPV-test in hrHPV-positive individuals with no CIN and hrHPV-positive individuals with a CIN 1 is based on a study of Rebolj and colleagues using data from the Danish Horizon study.^33^ The study presented concordance between the Hybrid Capture 2, cobas, CLART and Aptima hrHPV tests. We assumed that any prevalent hrHPV infection would be picked up by at least one of the four assays and defined the probability of a positive test result as the proportion of those total hrHPV infections that tested positive on the cobas hrHPV test.

**Table S7.** Test characteristics of the cytology test and the hrHPV-test by disease status

| Test result and disease status | Probability of a positive test result |
| --- | --- |
| *Abnormal cytology in case of no prevalent hrHPV infection** |  |
| - No CIN present | 0.60% |
| - CIN1 | 41.24% |
| - CIN2 | 42.25% |
| - CIN3 | 85.80% |
| - Cervical cancer | 85.09% |
| *Abnormal cytology in case of ≥1 prevalent hrHPV infection** |  |
| - No CIN present | 17.08% |
| - CIN1 | 41.24% |
| - CIN2 | 42.25% |
| - CIN3 | 85.80% |
| - Cervical cancer | 85.09% |
| *High-grade (HSIL, ASC-H, AGC or AIS) cytology in case of no prevalent hrHPV infection** |  |
| - No CIN present | 0.04% |
| - CIN1 | 2.98% |
| - CIN2 | 12.19% |
| - CIN3 | 39.47% |
| - Cervical cancer | 69.79% |
| *High-grade (HSIL, ASC-H, AGC or AIS) cytology in case of ≥1 prevalent hrHPV infection** |  |
| - No CIN present | 0.00% |
| - CIN1 | 2.98% |
| - CIN2 | 12.19% |
| - CIN3 | 39.47% |
| - Cervical cancer | 69.79% |
| *Positive hrHPV-test, in case of no prevalent hrHPV infection* | 0% |
| *Positive hrHPV-test, in case of ≥1 prevalent hrHPV infection* |  |
| - No CIN present | 69% |
| - CIN1 | 72% |
| - CIN2 | 94% |
| - CIN3 | 94% |
| - Cervical cancer | 94% |

* Probability to test positive the first time an individual with this lesion present attends screening. 12% of the CIN lesions will be missed systematically over time.

hrHPV = high-risk human papillomavirus; CIN = cervical intraepithelial neoplasia; HSIL = High-grade squamous intraepithelial lesion; ASC-H = atypical squamous cells cannot exclude HSIL; AGC = atypical glandular cells , AIS = adenocarcinoma in situ.

## Model calibration

The values of some model parameters in the parameter overview could either be based on observed data or on available literature. However, some other model parameters could not be derived from observational data (e.g., the age-specific background risk for acquiring an hrHPV-infection). Therefore, these parameter values needed to be calibrated.

In the calibration of MISCAN-Cervix, a population is simulated for which high quality observational data is available over a time span of multiple years, including the screening behaviour of that population. In this case we used the Ontario population with a cervix in 2010-2017 and simulated their screening behaviour in the cytology program. It should be noted that the Ontario data for screening participation, incidence and mortality uses the female registered population as denominator. From around 2017, Ontario people can change the sex on their health cards and as such this female registered population no longer directly reflects everyone with a cervix. This introduces a slight bias into the model, but is expected to have negligible effects on model outputs.

The parameters that could not be derived from observational data or literature are estimated based on expert opinion or studies on similar parameters. The model runs with this set of parameter values after which the outputs of the model are compared with the observed data of that population on:

- Cervical cancer incidence by age
- Cervical cancer stage distribution by age
- hrHPV prevalence by age cytology result, lesion grade and genotype
- Detection rates.

Based on this comparison, the model inputs are adjusted using the calibration algorithm. With these new inputs the model runs again where the calibration cycle starts again (Figure S11).^34^ This cycle is repeated until the outputs of the model reflect the observed data well.


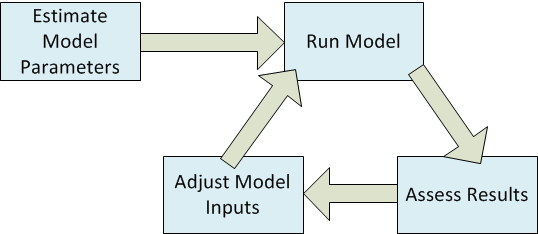


Figure S11. Calibration cycle

### Stepwise process

Due to the large number of parameters that need to be calibrated, we used a three-step approach.

*Step 1*

First, we isolated the infections that would progress to cancer, by setting all progression probabilities at 1. Then the parameters that are related to the development of cancer are calibrated on the provided Ontario cancer incidence, stage distribution and cancer detection rates. These durations and probabilities that are calibrated in step 1 are represented in Figure S12 by yellow ellipses and green ellipses respectively.

- Duration of progressive CIN3, including the shape of the corresponding Weibull distribution
- Duration of FIGO1A, FIGO1B, FIGO2, FIGO3 and FIGO4
- The probability that a cancer is clinically detected, before progressing to a higher stage, when in stage FIGO1B, FIGO2 or FIGO3. Subsequently, the probability that a cancer grows into a higher stage without getting detected is one minus this previous probability. Please note that FIGO1A cancers are assumed not to give symptoms yet and can only be screen-detected, while FIGO4 cancers already reached the highest stage and can only transition to the clinical state from there.
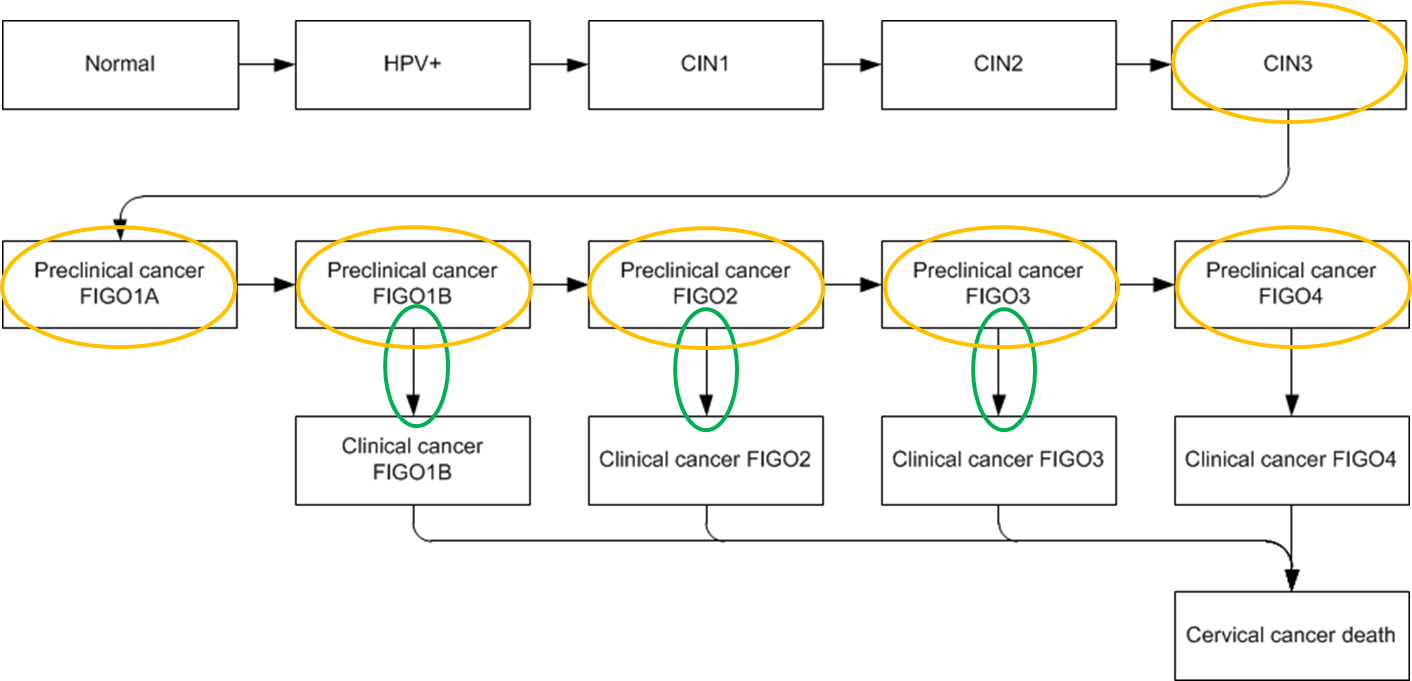


Figure S12. Calibrated durations and probabilities during step 1 of the calibration process

HPV = human papillomavirus; CIN = cervical intraepithelial neoplasia; FIGO = International Federation of Gynaecology and Obstetrics.

*Step 2*

In the second step, we introduce clearance of hrHPV and regression of CIN lesions to the model to calibrate the parameters that are related to the development of precancerous lesions (Figure S13).

- Duration of regressing CIN3 (yellow ellipse)
- Hazard rate for an hrHPV infection
- Hazard rate for the development of a CIN1 in absence of hrHPV
- The age-specific probability of progression of hrHPV/CIN1/CIN2/CIN3 to a higher stage


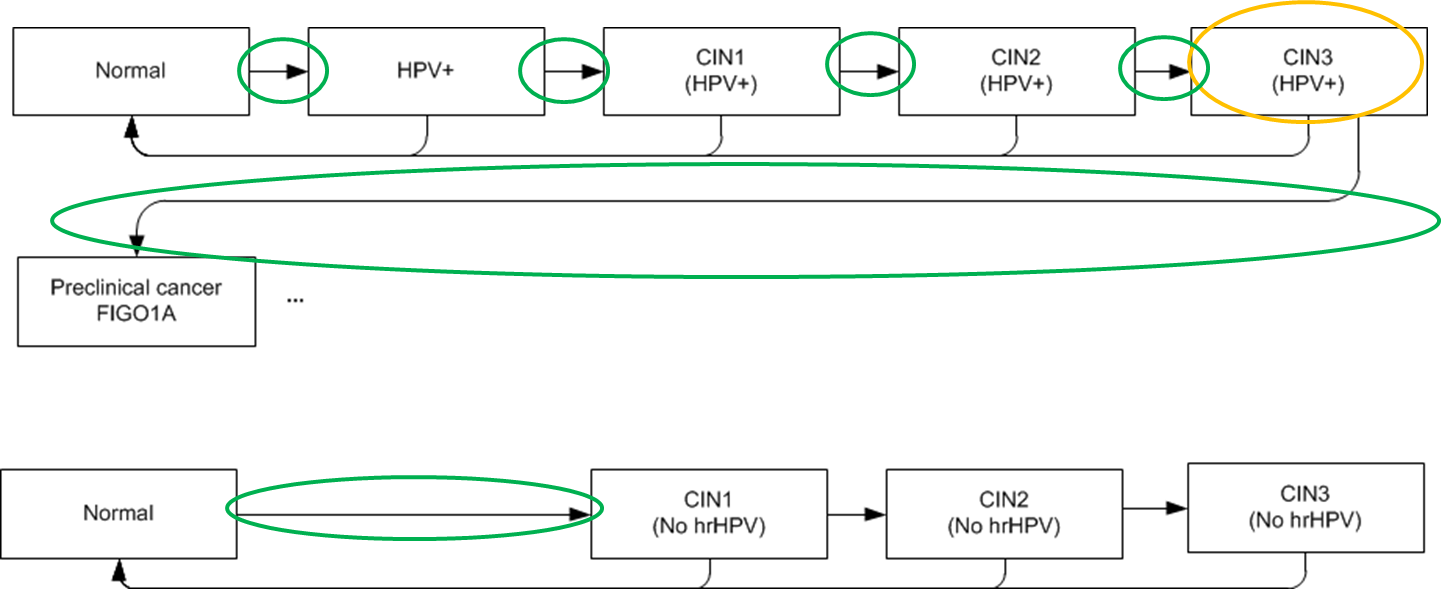


Figure S13. Calibrated durations and probabilities during step 2 of the calibration process

HPV = human papillomavirus; CIN = cervical intraepithelial neoplasia; FIGO = International Federation of Gynaecology and Obstetrics.

*Step 3*

In the third and last step of the calibration process, the progression probabilities of hrHPV-infections and CIN lesions will be made type-specific to create the 5 disease pathways of the model. The reason that these probabilities need to be type-specific is that some types have been found to be more carcinogenic and therefore have a higher probability of progressing than others. As a starting point, all age-specific progression probabilities are assumed to be equal to those in the second step of the calibration, but a type-specific and disease stage-specific multiplication factor will be applied and adjusted by the calibration algorithm to obtain different progression probabilities by hrHPV-type, indicated by the green ellipses in Figure S14.


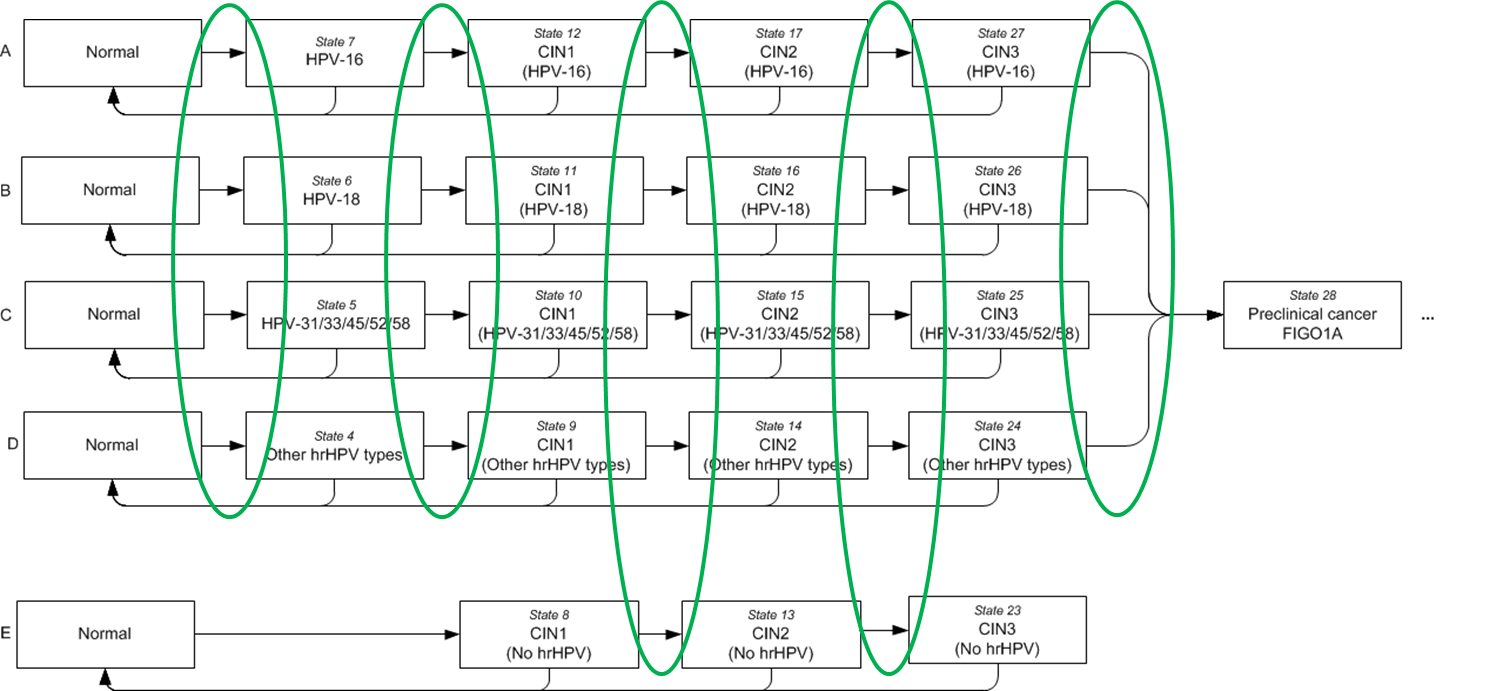


**Figure S14.** Calibrated probabilities during step 3 of the calibration process

HPV = human papillomavirus; CIN = cervical intraepithelial neoplasia; FIGO = International Federation of Gynaecology and Obstetrics.

# Calibration results

Model fits against calibration targets for various outcomes are shown in figures 1 to 7. The methodology behind the calibration process can be found in more detail in the previous D6 Model Description. In these figures, the black dots represent observational target data. The red line shows the output of the model after recalibration towards the 7 different observed Ontario targets. The orange line shows the fit before calibration using the existing Dutch model parameters.

The first target of cervical cancer incidence is one of the most important ones for modelling screening effects. This considers both screen-detected cancers and clinically detected cancers unrelated to screening. It is calibrated by changing onset of high-risk HPV infections, regression rates of precancerous lesions and clinical transition rates for different cancer stages. Figure S15 shows that the incidence rate fits the observed data well (i.e. within the observed confidence intervals), except for age group 60-79 in which the simulated incidence rates are a bit too low. It seems the dip in cancer incidence at ages around the termination of screening is more pronounced in the model than in real observed data


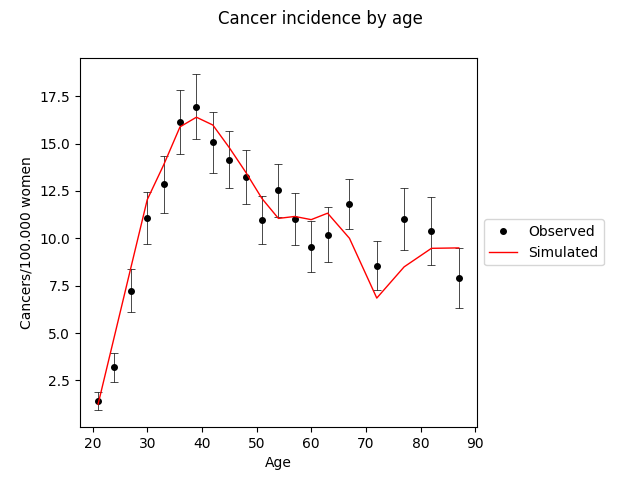


**Figure S15.** Cervical cancer incidence per 100,000 individuals in Ontario, Canada, model predictions compared to observational data. Solid red line shows the calibrated cervical cancer incidence, and the orange line shows the incidence using starting values of calibration.

Next, the modelled HPV prevalence level was calibrated by adjusting the age-specific risks of infection and the age-specific probabilities of clearance of the infection or developed lesion.
Unfortunately, no recent accurate data was found to represent Ontario HPV prevalence. The HPV prevalence target thus uses Montreal CCCaST ^24^ prevalence data for older ages 30-69 which is complemented with British Columbia ^25^ data for the younger age groups. The model fit on age-specific high-risk HPV prevalence is presented in figure S16.


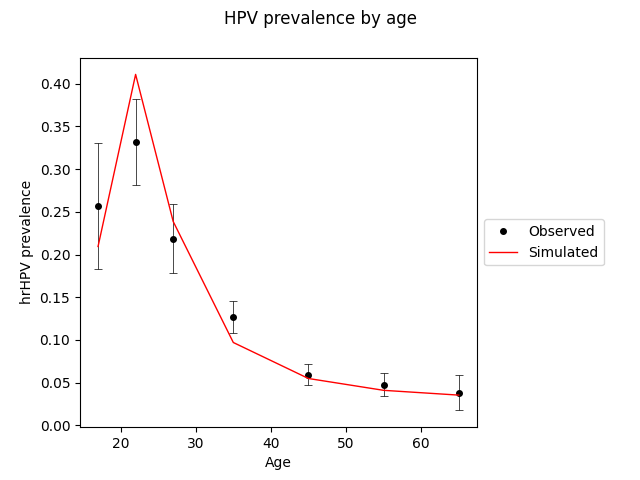


**Figure S16.** High-risk HPV prevalence by age.

The cervical cancer stage distributions (i.e. proportion of cancer within a certain cancer stage) are modelled explicitly and will largely determine the cervical cancer mortality. The modelled stage distribution before and after calibration is presented in figure S17. The FIGO1A stage is assumed to only be detected by screening and as such the modelled FIGO1A detection rates are zero from age 70, whereas there are small proportions of FIGO1A reported in the observed data. Apart from this, the stage distribution mostly fits with the confidence interval of the observed data.


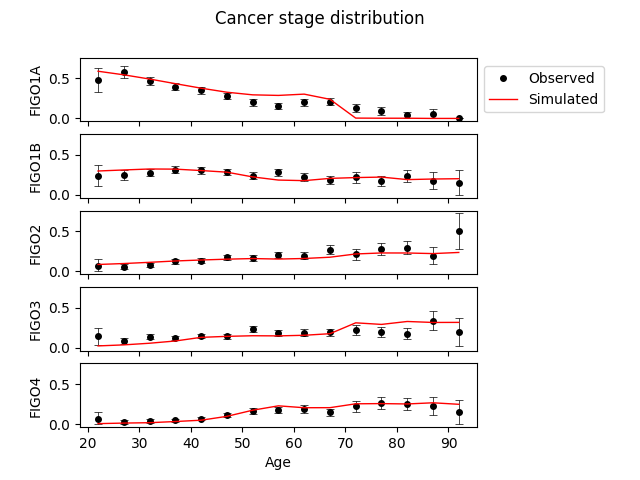


**Figure S17.** Stage distribution for different cancer stages by age.

The HPV type distributions within detected CIN lesions are modelled explicitly, using four different groups. HPV-16 and HPV-18 both have their own group. The next group, HPV-9v consists of the other carcinogenic types covered by the nonavalent vaccine: 31, 33, 45, 52 and 58. Finally, the HPV-ohr denomination comprises all ‘other high-risk’ HPV types: 35, 39, 51, 56, 59, 66 and 68. The fit of these groups to data observed in New Mexico ^16^ is displayed below in figure S18 for different levels of CIN lesions. New Mexico data was chosen, because there was no Canadian source with sufficient information on HPV types The figure shows that the model correctly replicates the observed percentages of the specific HPV types in the different CIN stages.


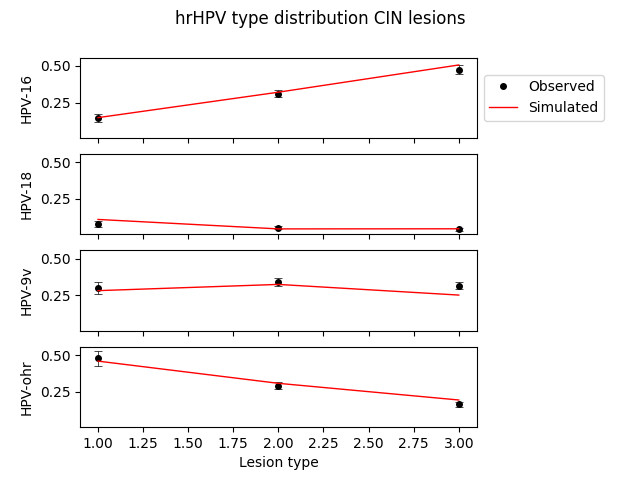


**Figure S18.** Relative high-risk HPV type distribution in different CIN grades.

Next, the model was also calibrated to fit CIN3 detection rates by low-grade or high-grade cytology results, currently observed in Ontario. The model generally follows the data, but finds slightly more CIN3 lesions as compared to the observed data, especially in young 21-24 year old. This is probably to be able to reach the high peak of cancers around age 30-40 considering an assumed mean CIN3 dwell time of around 14 years. An interesting observation is that about equally many CIN3 lesions are found through low-grade results as after high-grade cytology.


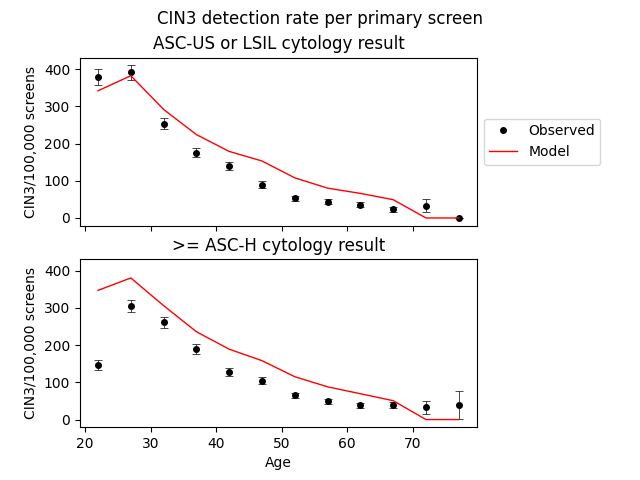


**Figure S19.** CIN3 detection rates by age for both low and high grade cytology results.

Additionally, the detection rate drops to zero in the model from age 70 onwards, because screening is assumed to be stopped at this age. In reality the program recommends an end age of 70 only if the person has a history of three subsequent negative pap smears. Otherwise screening is continued and thus a non-zero number of CIN’s are found for these ages over 70, unlike in the model.

Next, the cancer detection rate through either low or high grade cytology results is examined. The model overshoots the cancer detection from both low and high grade pap results, but the difference is more pronounced for high-grade cytology results. Since cancers detected from low grade smears are less common they have a wider confidence interval and allow for a slightly larger overestimation.

**
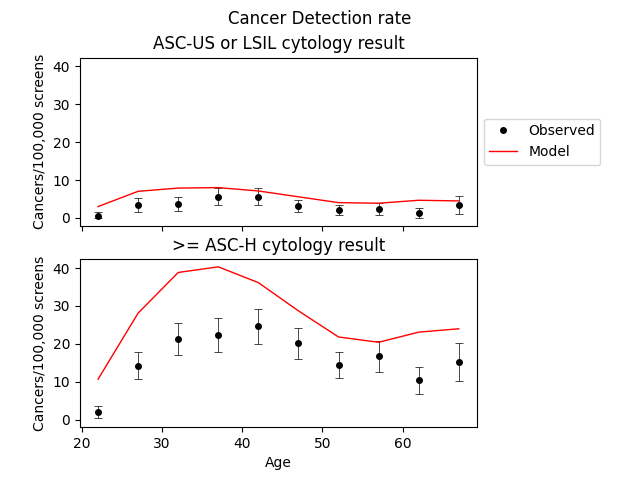
**

**Figure S20.** Cancer detection rates by age for both low and high grade cytology results.

The cancer detection rate is very strongly related to the cancer incidence level. Any attempt of reducing this detection rate would result in lower incidence levels, and incidence was given higher priority in the calibration process.

One explanation could be that the way the observed data is collected does not perfectly correspond with the way cancer detection is formulated in the model. For example, in the collected Ontario target data, individuals with a previous positive cytology test were excluded as well as undecided, missing and unsatisfactory Pap results. Furthermore the follow-up time was only one year from cytology to colposcopy and another year from colposcopy to cancer diagnosis. It is possible some cancers were missed in this data collection method for the detection metric, while these cases would still be counted in the incidence measure. In the literature, reported detection rates have proven to differ substantially between different contexts, for example in different European countries ^35^. This could also suggest a large uncertainty in data collection methods. As such, this target measure was decided to be left in a suboptimal fit. Do note that the calibrated model follows the shape of the observed data very nicely, suggesting a constant bias factor in the model formulation or data collection could explain the difference.

Finally, the model was validated on mortality (Figure S21). This measure was not used to fit the unobserved parameters, but provides an estimate of how well the model replicates observed cervical cancer deaths. It can be seen that the model slightly overestimates mortality for ages 50-70. This could be caused by limited data on cervical cancer survival, especially for follow up times exceeding five years. All three sources of data provided by Ontario for incidence, survival and mortality considered a specific range of 8 years 2010 up to 2017. However, due to the required follow-up time of 5 years, survival data is only available for the cancers found in year 2010 up to 2012. The uncertainty within the input survival data is estimated through 95% confidence bounds as displayed in the figure. The large amount of overlap between the survival input confidence bounds and the target mortality confidence bounds provides a sufficient level of trust in the model predictions.


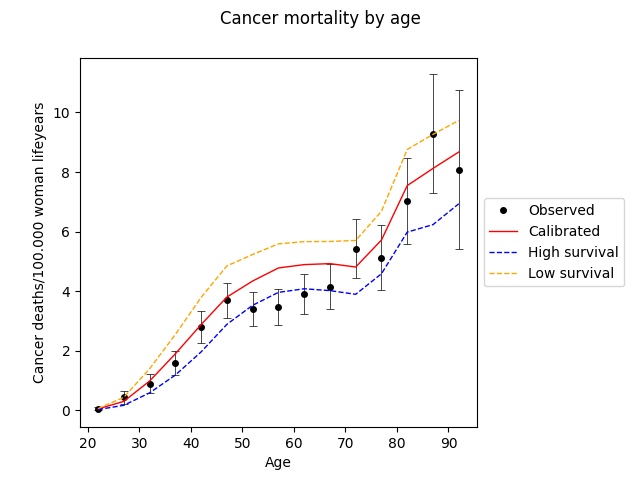


**Figure S21.** Cancer mortality by age. The dashed lines represent 95% confidence intervals on the input parameters for age and stage specific survival rates.

# Summary tables of model data and assumptions.

| **Demographic data** | **Details** | **Source** |
| --- | --- | --- |
| Population size | By age, 2011 | Statistics Canada |
| All-cause mortality | By age, 2010-2017 | Statistics Canada |
| Hysterectomy rate | By year 2008-2018 and age group | OHIP & Statistics Canada |

**Table S8. Demographic data sources**

**Table S9. Observed target data**

| **#** | **Calibration Target** | **Details** | **Source** |
| --- | --- | --- | --- |
| 1 | Cervical cancer incidence | By age, 2010-2017 | Statistics Canada |
| 2 | Cervical cancer mortality | By age, 2010-2017, used for validation | Statistics Canada |
| 3 | hrHPV prevalence | By age group | CCCaST and BC data (see section 8.) |
| 4 | Stage distribution | By age group, 2010-2017 | Ontario Cancer Registry (shared by Ontario Health) |
| 5 | Survival | By age group, 2010-2017 | Ontario Cancer Registry (shared by Ontario Health) |
| 6 | Detection rates from cytology | By year (2008-2017), by age group and by cytology outcome | CytoBase, OHIP, OCR, RPDB* (shared by Ontario Health) |
| 7 | Cytology outcomes (high/low) | By year (2008-2018) and age group | CytoBase, OHIP, OCR, RPDB* (shared by Ontario Health) |

**Table S10. Natural history assumptions for hrHPV, pre-cancer and cervical cancer development**

| **Assumption** | **Details** | **Source** |
| --- | --- | --- |
| Maximum infections = 50 | Over 50 lesions in an individual no longer represents the average risk population. Limited for efficiency. | Modelling choice. |
| hrHPV onset | By age and type (16, 18, 9v and other high risk). | Calibrated using (1) and (3). |
| Non-hrHPV CIN1 onset | Age-specific. | Calibrated using (6) and (7). |
| Duration hrHPV infection** | Weibull(1, 1), equal across HPV types | Literature.^28, 29^ |
| Duration CIN1 lesion | Weibull(1.5, 1) , equal across HPV types | Literature.^30, 31^ |
| Duration CIN2 lesion | Weibull(2, 1) , equal across HPV types | Literature.^31^ |
| Duration CIN3 lesion | Regression(Weibull(5.7, 0.84)), progression (Weibull(14.3, 0.84)), equal across HPV types | Dutch calibration using interval cancer data. |
| Cancer durations | See Table S3 for details | Calibrated using (1), (4) and (6). |
| Regression probabilities hrHPV positive (HPV, CIN1, CIN2, CIN3) | Type- and age-specific. | Calibrated using (3), (6) and (7). |
| Regression probabilities HPV-negative lesions (CIN1, CIN2) | Age-specific. | Calibrated using (6) and (7). |
| Clinical transition probabilities (FIGO1B, FIGO2, FIGO3) | Age-specific. | Calibrated using (1) (4) and (6). |
| Probability of cancer-specific death after detection | Age-specific, Stage-specific | Calculated from (5) |
| Time to cancer-specific death since detection | Stage-specific | Calculated from (5) |

**Table S11. Screening assumptions**

| **Assumption** | **Details** | **Source** |
| --- | --- | --- |
| Test characteristics cytology | See Table S5. | Dutch calibration using interval cancer data. |
| Test characteristics HPV | See Table S5. | Literature^32, 33^ |
| Screening strategy | Annual screening (2000-2011), 3-yearly screening (2012-present) | Ontario Cancer Screening Report 2020 (shared by Ontario Health) |
| Percentage never attenders | 13.9% in cohort tracked between 2004-2018 | CytoBase, OHIP, OCR, RPDB (shared by Ontario Health) |
| Screening participation | Calculated based on never attenders and observed up-to-date rates, see Table S4. | CytoBase, OHIP, OCR, RPDB (shared by Ontario Health) |
| Program adherence (colpo and repeat tests) | See Table S4. | CytoBase, OHIP, OCR, RPDB (shared by Ontario Health) |

**Table S12. Vaccination assumptions**

| **Assumption** | **Details** | **Source** |
| --- | --- | --- |
| Vaccine type | Quadrivalent and Nonavalent (from 2016-2017) | Immunization Coverage Report (Public Health Ontario) |
| Vaccine age | 12 and 13 (before 2017-2018) | Immunization Coverage Report (Public Health Ontario) |
| Vaccine coverage | Boys included from 2016-2017 onwards | Immunization Coverage Report (Public Health Ontario) |
| Vaccine effectiveness | 95%, lifelong protection | Literature^36^ |
| Cross protection | 35% for Nonavalent HPV types, using the quadrivalent vaccine | Literature^37^ |

# HPV prevalence sources

For model calibration it is vital that a valid pre-vaccination hrHPV prevalence is determined. While an Ontario reference does exist^38^, it is relatively dated and has a very limited sample size of only a few hundred individuals. Several alternative sources are available and presented in Figure S22 below. All of them use the HC-2 HPV tests.


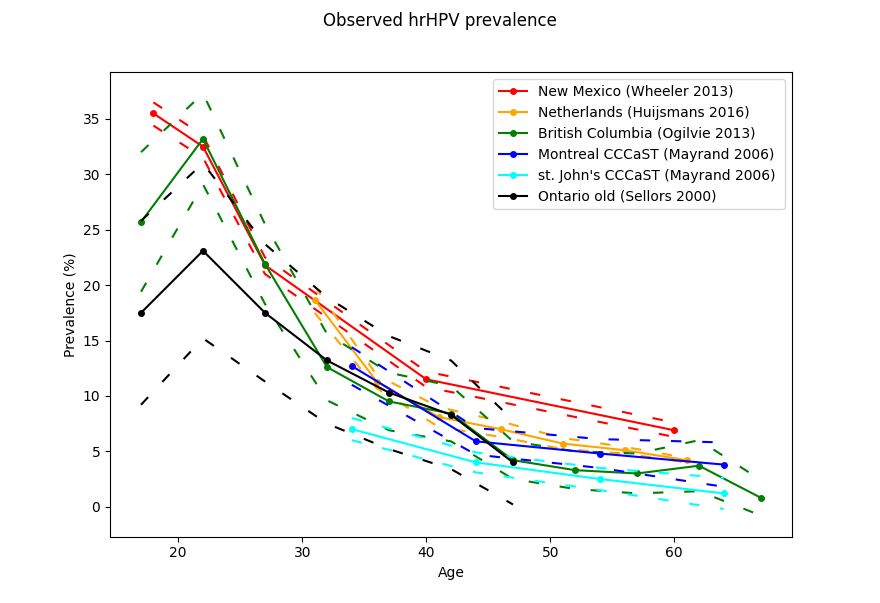


**Figure S22**. HPV prelance found in different settings including New Mexico^16^ (NM), The Netherlands^39^, British Columbia^25^ (BC), Montreal^24^, St. John’s^24^ and Ontario^38^. The dashed lines represent 95% confidence intervals.

It seems that 3 different data sources agree for higher ages, namely BC, Montreal and the older Ontario levels. Out of these, the Montreal CCCaST study seems most generalizable to the Ontario population, because of its geographical proximity and cultural similarity. Additionally, the Montreal CCCaST findings were most accurate in terms of sample size out of the three agreeing data sources. Unfortunately, these accurate findings are only limited to ages 30 and up. For the prevalence in younger ages a different source will need to be used. For this the BC prevalence rate seems the best candidate since it matches the NM prevalence for ages 20-30, but also follows the same drop in individuals younger than age 20 as seen in the older Ontario study.

# HPV-FRAME checklist

The checklist below is based on Canfell et al.(34) who created a quality-based framework for reporting on HPV modelling studies assessing epidemiological and economic policy questions.

|  | **Reported?**  **(Y/N)** | **Reported by age? (Y/N)** | **Report by sex (F-only, M-only or both)?** | **Comments** |
| --- | --- | --- | --- | --- |
| **Inputs** |  |  |  |  |
| Target population for intervention | Y | Y | Y | Methods |
| Sexual behaviour | Y | Y | Y | Supporting information 7.2 and reference to Matthijsse et al.(11) |
| Cohort examined for evaluation/time horizon | Y | Y | F-only | Ten most recently vaccine-eligible cohorts (1998-2007) as described in the Methods. |
| Quality of life assumptions | N/A | N/A | N/A | No QALYs used for harms-benefits analysis |
| Calibration | Y | Y | F-Only | Supporting information 7.6 |
| Validation (where possible) | N | N | N |  |
| Costs | N/A | N/A | N/A | No costs used in harms-benefits analysis |
| Vaccine uptake | N | N | N | Reference to Immunization Coverage Reports in Methods |
| Vaccine efficacy | Y | N | N | Reported in methods (95% lifelong). The same efficacy is assumed for males and females. |
| Vaccine cross-protection | Y | Does not vary with age | N | Model assumes protection for the targeted HPV types only as described in the Methods. |
| Duration vaccine protection and waning | Y | Does not vary with age | N | Lifelong protection as described in the Methods. This applies to both males and females. |
| Vaccine and delivery costs | N/A | N/A | N/A | No cost-effectiveness analysis included. |
| Pre-vaccination disease burden (including population attributable fractions for HPV) | Y | Y | F-Only | Type-specific HPV prevalence in STDSIM in Supplemental Material figure S5. Cervical cancer incidence and mortality rates in Supplemental Material Section 8. |
| Duration of natural immunity | Y | N | N | Mentioned in Additional file 1 on page 2 that this is described in Matthijse et al.(18) |
| Routine screening behaviour (routine and follow-up and test-of-cure) | Y | Y | N | Supporting Information 7.5.3 |
| Screening test(s) and colposcopy accuracies | Y | Does not vary with age | F-Only | Supportin Information Table S5 |
| Abnormal test management (primary and triage) | Y | Y | F-Only | Supporting Information Sections 2 and 3 |
| Diagnostic follow-up of abnormal tests | Y | Does not vary with age | F-Only | Supporting Information Section 3 |
| Management by disease grade (confirmed disease) | Y | Does not vary with age | F-Only | Supporting Information 7.3.4 |
| Sources of information for screening structure and parameterization | Y | N/A | F-Only | Described in Methods and Supporting Information Section 9 |
| HPV type incidence, clearance and progression rates | N | N | N | Calibrated |
| Herd effect | N | N | N | The effects of herd-immunity are taken into account by using the dynamic STDSIM model, but are not explicitly presented. |
| Association between vaccination and screening uptake | N | N/A | N/A |  |
| Fixed – variable costs[∗](https://www.sciencedirect.com/science/article/pii/S2405852119300230?via%3Dihub#tblA10fnlowast) | N/A | N/A | N/A | No cost-effectiveness analysis included. |
| **Outputs** |  |  |  |  |
| Cancer incidence, mortality, life years, QALYs/DALYs (as appropriate) | Y | N | F-Only | Full output tables in Supporting Information sections 4 and 5. Cancers and cancer deaths, rather than incidence and mortality for cohort-focused optimization |
| HPV prevalence, pre-intervention | Y | Y | F-Only | Sources in Supporting Information section 10, model calibrated prevalence in Supporting Information section 8. |
| CIN2 detected | Y | N | F-Only | Supporting Information section 5 |
| Sensitivity analysis on key inputs | Y | N | F-Only | See manuscript Table 2 and Supporting Information Table S1 |
| Incremental cost-effectiveness ratios and costs saved | N | N | N | No cost-effectiveness analysis included, but incremental harms-benefits ratio is reported |
| Absolute reductions in HPV infections, and/or warts, post-vaccination | N | N | N | Not the focus of this study. |
| Absolute reductions in CIN2+ post-vaccination | N | N | N | Not the focus of this study. |
| Absolute reductions in invasive cancer (cervical and other HPV cancers, as relevant) post-vaccination | N | N | N | Not the focus of this study. |

Abbreviations:

HPV = Human papillomavirus; QALYs = quality adjusted lifeyears; DALYs = disability adjusted lifeyears; CIN = cervical intraepithelial neoplasia.

# Extra sensitivity analysis with 4 year screen interval

**Unstratified Subgroup**

| Optimal |  |  |  |  |  |  |  |  |
| --- | --- | --- | --- | --- | --- | --- | --- | --- |
| 7-2 | 25 | 30 | 35 | 40 | 45 | 50 | 55 |  |
| Alternatives |  |  |  |  |  |  |  |  |
| 7-13 | 25 | 29 | 33 | 38 | 43 | 48 | 53 |  |
| 8-13 | 25 | 29 | 33 | 37 | 42 | 47 | 52 | 57 |

| **Strategy** | **Total screens** | **Colposcopy referrals** | **Harms** | **Cancers** | **Cancers Prevented** | **IHBR** | **AHBR** |
| --- | --- | --- | --- | --- | --- | --- | --- |
| No screening | 0 | 0 | 0 | 586.9 | 0 | NA | NA |
| hpv_16/18_1_2 | 104,081 | 2,751 | 131,595 | 348.6 | 238.3 | 552 | 552 |
| hpv_16/18_2_3 | 204,073 | 4,110 | 245,168 | 280.8 | 306.2 | 1,674 | 801 |
| hpv_16/18_2_1 | 209,917 | 6,059 | 270,511 | 265.8 | 321.2 | 1,690 | 842 |
| hpv_16/18_3_8 | 307,608 | 7,033 | 377,937 | 214.6 | 372.4 | 2,098 | 1,015 |
| hpv_16/18_4_6 | 409,399 | 8,487 | 494,270 | 179.5 | 407.4 | 3,318 | 1,213 |
| **hpv_16/18_7_2** | **537,932** | **16,765** | **705,586** | **129.8** | **457.1** | **4,251** | **1,544** |
| *hpv_16/18_7_13* | *545,710* | *18,066* | *726,366* | *134* | *453* | *Dom.* | *1,603* |
| hpv_16/18_8_3 | 632,572 | 17,521 | 807,786 | 111.4 | 475.5 | 5,568 | 1,699 |
| *hpv_16/18/9v_7_13* | *530,815* | *28,961* | *820,428* | *127* | *460* | *Dom.* | *1,784* |
| *hpv_16/18_8_13* | *640,952* | *18,973* | *830,686* | *113* | *474* | *Dom.* | *1,753* |
| hpv_16/18_7_6 | 724,132 | 18,243 | 906,565 | 100.7 | 486.2 | 9,204 | 1,865 |
| *hpv_16/18/9v_8_13* | *625,316* | *30,455* | *929,869* | *106* | *481* | *Dom.* | *1933* |
| hpv_16/18/9v_7_6 | 709,734 | 28,646 | 996,198 | 93 | 493.9 | 11,624 | 2,017 |
| *hpv_direct_colpo_7_13* | *501,614* | *51,052* | *1,012,138* | *119* | *468* | *Dom.* | *2,163* |
| hpv_16/18/9v_8_7 | 803,694 | 29,439 | 1,098,087 | 86.4 | 500.6 | 15,372 | 2,194 |
| *hpv_direct_colpo_8_13* | *593,977* | *54,231* | *1,136,289* | *96* | *491* | *Dom.* | *2,314* |
| hpv_direct_colpo_8_7 | 771,891 | 53,382 | 1,305,709 | 75.6 | 511.4 | 19,221 | 2,553 |
| hpv_direct_colpo_9_1 | 863,985 | 55,025 | 1,414,235 | 71.1 | 515.8 | 24,544 | 2,742 |

All alternative strategies were dominated by the existing efficiency frontier and the optimal strategy remains unchanged.

**Unvaccinated Subgroup**

| Optimal |  |  |  |  |  |  |  |  |  |
| --- | --- | --- | --- | --- | --- | --- | --- | --- | --- |
| 8-3 | 25 | 30 | 35 | 40 | 45 | 50 | 55 | 60 |  |
| Alternatives |  |  |  |  |  |  |  |  |  |
| 9-10 | 25 | 29 | 33 | 37 | 41 | 46 | 51 | 56 | 61 |
| 8-13 | 25 | 29 | 33 | 37 | 42 | 47 | 52 | 57 |  |

| **Strategy** | **Total screens** | **Colposcopy referrals** | **Harms** | **Cancers** | **Cancers Prevented** | **IHBR** | **AHBR** |
| --- | --- | --- | --- | --- | --- | --- | --- |
| no_screen | 0 | 0 | 0 | 900 | 0 | NA | NA |
| hpv_16/18_1_2 | 104,422 | 3,793 | 142,356 | 520.2 | 379.7 | 375 | 375 |
| hpv_16/18_2_3 | 204,489 | 5,529 | 259,774 | 417.7 | 482.3 | 1,145 | 539 |
| hpv_16/18_2_1 | 210,708 | 8,358 | 294,291 | 391.2 | 508.8 | 1,305 | 578 |
| hpv_16/18_3_8 | 308,467 | 9,587 | 404,339 | 315.5 | 584.4 | 1,454 | 692 |
| hpv_16/18_4_6 | 410,486 | 11,515 | 525,637 | 261.5 | 638.5 | 2,244 | 823 |
| hpv_16/18_7_2 | 541,375 | 23,162 | 772,993 | 181.7 | 718.3 | 3,101 | 1076 |
| **hpv_16/18_8_3** | **635,573** | **24,122** | **876,792** | **155.6** | **744.4** | **3,977** | **1178** |
| *hpv_16/18_8_13* | *644,510* | *26,252* | *907,030* | *157* | *743* | *Dom.* | *1221* |
| hpv_16/18_7_6 | 726,372 | 25,004 | 976,416 | 140.5 | 759.5 | 6,586 | 1286 |
| *hpv_16/18_9_10* | *740,339* | *27,209* | *1,012,432* | *136* | *764* | *8,003* | *1325* |
| *hpv_16/18/9v_8_13* | *624,975* | *40,414* | *1,029,112* | *148* | *752* | *Dom.* | *1369* |
| hpv_16/18_8_7 | 820,633 | 25,616 | 1076,796 | 131.1 | 768.9 | 10,648 | 1400 |
| *hpv_16/18/9v_9_10* | *720,109* | *41,878* | *1,138,889* | *127* | *773* | *15,145*  *(Ext. dom.)* | *1473* |
| hpv_16/18/9v_8_7 | 802,315 | 38,700 | 1189,315 | 121.8 | 778.2 | 12,099 | 1528 |
| *hpv_direct_colpo_8_13* | *594,923* | *62,907* | *1,223,993* | *139* | *761* | *Dom.* | *1608* |
| hpv_16/18/9v_9_1 | 895,766 | 39,327 | 1289,036 | 116.6 | 783.4 | 19,166 | 1645 |
| *hpv_direct_colpo_9_10* | *688,189* | *65,846* | *1,346,645* | *117* | *783* | *Dom.* | *1720* |
| hpv_direct_colpo_9_1 | 863,766 | 63,204 | 1495,806 | 106.1 | 793.9 | 19,737 | 1884 |

Most alternative strategies were dominated or extendedly dominated by the existing frontier. Only strategy 9_10 with HPV16/18 genotyping was found to appear on the efficiency frontier, but with an incremental harms benefits ratio much higher than the acceptability threshold of 4,721. The resulting optimal strategy remains unchanged by the addition of a 4 year interval.

# References

1. Andrae B, Kemetli L, Sparen P, Silfverdal L, Strander B, Ryd W, et al. Screening-preventable cervical cancer risks: Evidence from a nationwide audit in Sweden. Journal of the National Cancer Institute. 2008 May;100(9):622-9.

2. Landy R, Pesola F, Castanon A, Sasieni P. Impact of cervical screening on cervical cancer mortality: estimation using stage-specific results from a nested case-control study. Br J Cancer. 2016 Oct 25;115(9):1140-6.

3. Lönnberg S, Nieminen P, Luostarinen T, Anttila A. Mortality audit of the Finnish cervical cancer screening program. Int J Cancer. 2013;132(9):2134-40.

4. Habbema JD, van Oortmarssen GJ, Lubbe JT, van der Maas PJ. The MISCAN simulation program for the evaluation of screening for disease. Comput Methods Programs Biomed. 1985 May;20(1):79-93.

5. Hontelez JA, de Vlas SJ, Tanser F, Bakker R, Bärnighausen T, Newell ML, et al. The impact of the new WHO antiretroviral treatment guidelines on HIV epidemic dynamics and cost in South Africa. PLoS One. 2011;6(7):e21919.

6. Orroth KK, Freeman EE, Bakker R, Buvé A, Glynn JR, Boily MC, et al. Understanding the differences between contrasting HIV epidemics in east and west Africa: results from a simulation model of the Four Cities Study. Sex Transm Infect. 2007 Aug;83 Suppl 1:i5-16.

7. Korenromp E, Vliet C, Bakker R, de Vlas S, Dik J, Habbema F. HIV spread and partnership reduction for different patterns of sexual behaviour - A study with the microsimulation model STDSIM. Mathematical Population Studies. 2000;8:135-73.

8. Van der Ploeg CPB, Van Vliet C, De Vlas SJ, Ndinya-Achola JO, Fransen L, Van Oortmarssen GJ, et al. STDSIM: A Microsimulation Model for Decision Support in STD Control. Interfaces. 1998;28(3):84-100.

9. Matthijsse SM, Hontelez JA, Naber SK, Rozemeijer K, de Kok IM, Bakker R, et al. Public Health Benefits of Routine Human Papillomavirus Vaccination for Adults in the Netherlands: A Mathematical Modeling Study. J Infect Dis. 2016 Sep 15;214(6):854-61.

10. Matthijsse SM, Hontelez JAC, Naber SK, van Rosmalen J, Rozemeijer K, Penning C, et al. The estimated impact of natural immunity on the effectiveness of human papillomavirus vaccination. Vaccine. 2015 Oct 5;33(41):5357-64.

11. Matthijsse SM, van Rosmalen J, Hontelez JA, Bakker R, de Kok IM, van Ballegooijen M, et al. The role of acquired immunity in the spread of human papillomavirus (HPV): explorations with a microsimulation model. PLoS One. 2015;10(2):e0116618.

12. Bulkmans NW, Rozendaal L, Snijders PJ, Voorhorst FJ, Boeke AJ, Zandwijken GR, et al. POBASCAM, a population-based randomized controlled trial for implementation of high-risk HPV testing in cervical screening: design, methods and baseline data of 44,102 women. Int J Cancer. 2004 May 20;110(1):94-101.

13. Huijsmans CJJ, Geurts-Giele WR, Leeijen C, Hazenberg E, Mulder D, Van der Linden JC. Cervical cancer screening in the Netherlands: determination of HPV prevalence using three different systems. Presentation at the European Meeting on Molecular Diagnostics; 2015; 2015.

14. Prevention CfDCa. National Survey of Family Growth (NSFG); 2010-2011.

15. Prevention CfDCa. National Health and Nutrition Examination Survey (NHANES); 2011-2012.

16. Wheeler CM, Hunt WC, Cuzick J, Langsfeld E, Pearse A, Montoya GD, et al. A population-based study of human papillomavirus genotype prevalence in the United States: baseline measures prior to mass human papillomavirus vaccination. Int J Cancer. 2013 Jan 1;132(1):198-207.

17. van der Maas PJ, de Koning HJ, van Ineveld BM, van Oortmarssen GJ, Habbema JD, Lubbe KT, et al. The cost-effectiveness of breast cancer screening. Int J Cancer. 1989 Jun 15;43(6):1055-60.

18. Loeve F, Boer R, van Oortmarssen GJ, van Ballegooijen M, Habbema JD. The MISCAN-COLON simulation model for the evaluation of colorectal cancer screening. Comput Biomed Res. 1999 Feb;32(1):13-33.

19. van den Akker-van Marle ME, van Ballegooijen M, van Oortmarssen GJ, Boer R, Habbema JD. Cost-effectiveness of cervical cancer screening: comparison of screening policies. J Natl Cancer Inst. 2002 Feb 6;94(3):193-204.

20. Draisma G, Etzioni R, Tsodikov A, Mariotto A, Wever E, Gulati R, et al. Lead time and overdiagnosis in prostate-specific antigen screening: importance of methods and context. J Natl Cancer Inst. 2009 Mar 18;101(6):374-83.

21. Ten Haaf K, de Koning HJ. Should Never-Smokers at Increased Risk for Lung Cancer Be Screened? J Thorac Oncol. 2015 Sep;10(9):1285-91.

22. Walboomers JM, Jacobs MV, Manos MM, Bosch FX, Kummer JA, Shah KV, et al. Human papillomavirus is a necessary cause of invasive cervical cancer worldwide. J Pathol. 1999 Sep;189(1):12-9.

23. Quint W, Jenkins D, Molijn A, Struijk L, van de Sandt M, Doorbar J, et al. One virus, one lesion--individual components of CIN lesions contain a specific HPV type. J Pathol. 2012 May;227(1):62-71.

24. Mayrand MH, Duarte-Franco E, Coutlée F, Rodrigues I, Walter SD, Ratnam S, et al. Randomized controlled trial of human papillomavirus testing versus Pap cytology in the primary screening for cervical cancer precursors: design, methods and preliminary accrual results of the Canadian cervical cancer screening trial (CCCaST). Int J Cancer. 2006 Aug 1;119(3):615-23.

25. Ogilvie GS, Cook DA, Taylor DL, Rank C, Kan L, Yu A, et al. Population-based evaluation of type-specific HPV prevalence among women in British Columbia, Canada. Vaccine. 2013 Feb 4;31(7):1129-33.

26. Wheeler CM, Hunt WC, Cuzick J, Langsfeld E, Robertson M, Castle PE, et al. The influence of type-specific human papillomavirus infections on the detection of cervical precancer and cancer: A population-based study of opportunistic cervical screening in the United States. Int J Cancer. 2014 Aug 1;135(3):624-34.

27. Coupe VM, Berkhof J, Bulkmans NW, Snijders PJ, Meijer CJ. Age-dependent prevalence of 14 high-risk HPV types in the Netherlands: implications for prophylactic vaccination and screening. Br J Cancer. 2008 Feb 12;98(3):646-51.

28. Berkhof J, de Bruijne MC, Zielinski GD, Meijer CJ. Natural history and screening model for high-risk human papillomavirus infection, neoplasia and cervical cancer in the Netherlands. Int J Cancer. 2005 Jun 10;115(2):268-75.

29. Schiffman M, Castle PE, Jeronimo J, Rodriguez AC, Wacholder S. Human papillomavirus and cervical cancer. Lancet. 2007 Sep 8;370(9590):890-907.

30. Moscicki AB, Shiboski S, Hills NK, Powell KJ, Jay N, Hanson EN, et al. Regression of low-grade squamous intra-epithelial lesions in young women. Lancet. 2004 Nov 6-12;364(9446):1678-83.

31. Moscicki AB, Ma Y, Wibbelsman C, Darragh TM, Powers A, Farhat S, et al. Rate of and risks for regression of cervical intraepithelial neoplasia 2 in adolescents and young women. Obstet Gynecol. 2010 Dec;116(6):1373-80.

32. Bulk S, Bulkmans NW, Berkhof J, Rozendaal L, Boeke AJ, Verheijen RH, et al. Risk of high-grade cervical intra-epithelial neoplasia based on cytology and high-risk HPV testing at baseline and at 6-months. Int J Cancer. 2007 Jul 15;121(2):361-7.

33. Rebolj M, Bonde J, Preisler S, Ejegod D, Rygaard C, Lynge E. Differential Detection of Human Papillomavirus Genotypes and Cervical Intraepithelial Neoplasia by Four Commercial Assays. J Clin Microbiol. 2016 Nov;54(11):2669-75.

34. Neddermeijer H.G. vOGJ, Piersma N., Dekker R., Habbema J.D.F. Adaptive extensions of the Nelder and Mead simplex method for optimization of stochastic simulation models: Erasmus University Rotterdam, Econometric Institute; 2000.

35. Ponti A. AA, Ronco G., Senore C. Cancer Screening in the European Union. Report on the implementation of the Council Recommendation on cancer screening. Brussels: European Commission; 2017.

36. Arbyn M, Xu L. Efficacy and safety of prophylactic HPV vaccines. A Cochrane review of randomized trials. Expert Review of Vaccines. 2018 2018/12/02;17(12):1085-91.

37. Skinner SR, Apter D, De Carvalho N, Harper DM, Konno R, Paavonen J, et al. Human papillomavirus (HPV)-16/18 AS04-adjuvanted vaccine for the prevention of cervical cancer and HPV-related diseases. Expert Review of Vaccines. 2016 2016/03/03;15(3):367-87.

38. Sellors JW, Mahony JB, Kaczorowski J, Lytwyn A, Bangura H, Chong S, et al. Prevalence and predictors of human papillomavirus infection in women in Ontario, Canada. Survey of HPV in Ontario Women (SHOW) Group. Cmaj. 2000 Sep 5;163(5):503-8.

39. Huijsmans CJ, Geurts-Giele WR, Leeijen C, Hazenberg HL, van Beek J, de Wild C, et al. HPV Prevalence in the Dutch cervical cancer screening population (DuSC study): HPV testing using automated HC2, cobas and Aptima workflows. BMC Cancer. 2016 Nov 28;16(1):922.
